# Enhancing mitosis quantification and detection in meningiomas with computational digital pathology

**DOI:** 10.1186/s40478-023-01707-6

**Published:** 2024-01-11

**Authors:** Hongyan Gu, Chunxu Yang, Issa Al-kharouf, Shino Magaki, Nelli Lakis, Christopher Kazu Williams, Sallam Mohammad Alrosan, Ellie Kate Onstott, Wenzhong Yan, Negar Khanlou, Inma Cobos, Xinhai Robert Zhang, Neda Zarrin-Khameh, Harry V. Vinters, Xiang Anthony Chen, Mohammad Haeri

**Affiliations:** 1 Electrical and Computer Engineering, University of California, Los Angeles, Los Angeles, CA 90095 USA; 2 Pathology and Laboratory Medicine, The University of Kansas Medical Center, Kansas City, KS 66160 USA; 3 Pathology and Laboratory Medicine, UCLA David Geffen School of Medicine, Los Angeles, CA 90095 USA; 4 Department of Pathology, Stanford Medical School, Stanford, CA 94305 USA; 5 McGovern Medical School, UT Health at Houston, Houston, TX 77030 USA; 6 Baylor College of Medicine, Houston, TX 77030 USA

**Keywords:** Mitosis, Meningioma, Depth-first search, Pathologist group decision, Digital pathology

## Abstract

Mitosis is a critical criterion for meningioma grading. However, pathologists’ assessment of mitoses is subject to significant inter-observer variation due to challenges in locating mitosis hotspots and accurately detecting mitotic figures. To address this issue, we leverage digital pathology and propose a computational strategy to enhance pathologists’ mitosis assessment. The strategy has two components: (1) A depth-first search algorithm that quantifies the mathematically maximum mitotic count in 10 consecutive high-power fields, which can enhance the preciseness, especially in cases with borderline mitotic count. (2) Implementing a collaborative sphere to group a set of pathologists to detect mitoses under each high-power field, which can mitigate subjective random errors in mitosis detection originating from individual detection errors. By depth-first search algorithm (1) , we analyzed 19 meningioma slides and discovered that the proposed algorithm upgraded two borderline cases verified at consensus conferences. This improvement is attributed to the algorithm’s ability to quantify the mitotic count more comprehensively compared to other conventional methods of counting mitoses. In implementing a collaborative sphere (2) , we evaluated the correctness of mitosis detection from grouped pathologists and/or pathology residents, where each member of the group annotated a set of 48 high-power field images for mitotic figures independently. We report that groups with sizes of three can achieve an average precision of 0.897 and sensitivity of 0.699 in mitosis detection, which is higher than an average pathologist in this study (precision: 0.750, sensitivity: 0.667). The proposed computational strategy can be integrated with artificial intelligence workflow, which envisions the future of achieving a rapid and robust mitosis assessment by interactive assisting algorithms that can ultimately benefit patient management.

## Introduction

Meningioma is the most common primary brain tumor, accounting for approximately 40% of central nervous system (CNS) neoplasms in the United States [1]. According to the 2021 WHO classification of tumors of the CNS, (WHO CNS 5 Blue Book), the mitotic count (MC), calculated in 10 consecutive high-power fields (HPFs, 1HPF=0.16$mm^2$) from areas of the highest mitotic activity on H&E slides, is one critical criterion for meningioma grading [2]. Nonetheless, pathologists’ evaluation of MC varies due to obstacles in pinpointing hotspot areas, first, and detecting mitosis events within these areas [3–5]. Therefore, it is essential to create a more robust solution to ensure a reliable MC assessment for grading of meningiomas, which is important in clinical management and prognosis [6–8].

Besides histologic examination of the H&E slides, immunohistochemistry (IHC) staining has been used to assist with more accurate MC. For example, a proliferation index, derived from Ki-67 IHC, has been employed as a tool to correlate with the mitotic activity [9–11]. However, the Ki-67 proliferation index cannot fully replace the mitotic count, likely due to variation in staining among institutions, subjectivity of percentage assessment, and the absence of a clear threshold cutoff [12]. More recently, other IHCs such as Phosphohistone-H3 (PHH3) immunostains – effective in detecting both G2 and mitosis phases [13] – proved to be a reliable indicator for mitosis reading [14–17]. However, the PHH3 is not an acceptable criterion in the WHO criteria and thus, is mainly used in research studies.

It is noteworthy that the WHO CNS 5 Blue Book has incorporated molecular alterations as an alternative pathway for diagnosing grade 3 meningiomas, described as harboring a *TERT* promoter mutation and/or homozygous loss of *CDKN2A/B* [2]. Recent works on methylation profiling can also classify grade 3 meningiomas [18]. However, there are no clear molecular criteria for the majority of WHO 1 and 2 meningiomas, which constitute over 97% of meningiomas [1]. While the current WHO CNS guideline may not be the best predictor of tumor outcome, it is still not clear whether DNA copy number analysis, methylation profiling, or other factors are the better predictor of tumor recurrence and aggressive behavior [19–22]. Further investigation in methylation profiling and other molecular alterations along with clinical studies are still required to correlate with new approaches of tumor grading and classification.

As of 2023, histologic examination and evaluation of mitotic activity by pathologists still remain an important element for meningioma grading, considering its cost effectivity. However, this process is prone to a high level of variation because of two factors: (1) lack of standardized protocols for selecting 10 consecutive HPFs, resulting in differences in evaluated areas [3, 23]; and (2) inconsistencies in detecting mitosis within the selected 10 HPFs, stemming from controversy in mitosis verification, the small size, often low prevalence in grade 1 and most of grade 2 tumors, and heterogeneous distribution of mitotic figures [4, 5, 24].

The recent advancements in digital pathology and artificial intelligence (AI) technologies have shown promise in assisting pathologists in mitosis examination: A study of Aubreville et al. demonstrated that AI could outperform pathologists in localizing mitotic hotspots from digitized pathology slides [23]. AI-assisted systems have been developed to calculate and recommend hotspots equivalent to 10 consecutive HPFs for pathologists [25, 26]. The use of AI can save pathologists’ effort in searching for mitoses under high magnification, leading to improved sensitivities [25, 27], agreement rates [28], and confidence [26] in identifying mitotic figures. However, the current AI approaches have two major limitations:

1. AI-assisted systems typically calculate mitosis hotspots as circle [29], square [25] or rectangular [26, 27, 30, 31] regions, which may not completely align with the actual distribution of mitosis in tumors. As a result, the number of mitoses included in these recommended hotspot areas might be fewer, as the morphology of these hotspots lacks flexibility and nuance.
2. The current mitosis AI is trained on data from limited sources, such as specific patients, hospitals, and scanners [30–33]. Consequently, the AI may not achieve optimal performance when confronted with new and diverse data [34–36]. The pathologists performing histological analysis are still indispensable under such situations.

This work overcomes the limitations of current computational methods for mitosis quantification and detection by introducing a strategy that harnesses the preciseness of the computer algorithm and the collective intelligence of human pathologists. The first element of the strategy aims to enhance quantification, consisting of a computer algorithm called “depth-first search” (DFS). It can calculate the mathematically maximum possible MC in 10 consecutive HPFs of a slide, along with their corresponding locations. By analyzing 19 fully-annotated meningioma slides, we demonstrate that the DFS algorithm can identify 4.29 more and 3.32 more mitoses in 10 consecutive HPFs on average, compared to mitoses counted in linear and rectangular HPF arrangements, respectively. As a result, the elevated mitoses counted by the proposed DFS algorithm led to potential upgrades of two cases compared to the previous diagnoses verified by consensus conferences. The second element of the computational strategy targets to improve mitosis detection by leveraging the collective expertise of a group of pathologists. This approach involves letting a certain number of pathologists annotate mitoses independently and generating a final judgment through a majority vote. To validate this element, we hosted a user study involving 41 pathologists and pathology residents, where each participant independently annotated mitoses based on their own judgments in 48 selected HPFs of meningiomas. We evaluated the correctness of annotations from each participant, as well as the majority voting decisions generated from randomly-sampled subgroups. We report that groups of three pathologists and/or pathology residents can achieve an average precision of 0.897 and sensitivity of 0.699 in mitosis detection, which is higher than an average pathologist, who had a precision of 0.750 and a sensitivity of 0.667.

## Materials and methods

### Meningioma specimen preparation and mitosis annotation

All specimens were collected from the department of Pathology and Laboratory Medicine, University of California, Los Angeles. A total of 22 slides were selected based on the size of the tissue on the H&E slide and the availability of the corresponding Ki-67 IHC slide. These slides were from 12 patients (6 males, 6 females, age range between 39 and 79 years), including two WHO grade 1, six WHO grade 2, and four WHO grade 3 meningiomas. The grade 2 and grade 3 tumors for each slide were based on mitotic counts. Other criteria for upgrading to a grade 2 or 3 tumor were absent. None of these cases had available molecular testing such as *CDKN2A/B* and/or *TERT* promoter status. The specimens were collected between October 2019 and December 2021. The formalin fixed paraffin embedded blocks were sectioned at 6$\mu m$ thickness and stained with H&E followed by whole slide scanning (at 400$\times$ total scanning resolution with a 20$\times$ objective; Leica Aperio CS2). The same slide was then destained and immunostained with PHH3 antibody (1:200, Cell Marque, 369A-16) followed by rescanning of the slide with the same settings used in the first scan.

**Fig. 1:**
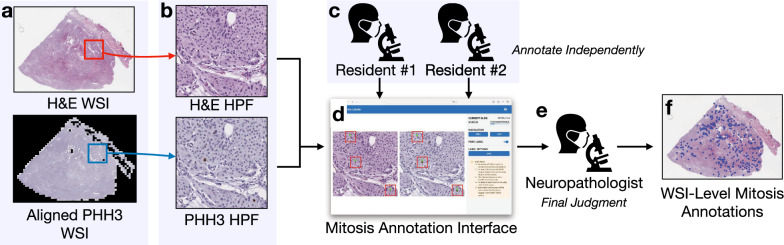
Workflow for mitosis annotation. **a** H&E and aligned PHH3 WSIs were prepared. **b** H&E and PHH3 image tiles with a size of one HPF were cut from corresponding WSIs. **c** Two pathology residents annotated mitoses on H&E-PHH3 HPF tiles independently using **d** the mitosis annotation interface. **e** A neuropathologist reviewed mitosis annotations from the two pathology residents and provided the final judgment for each one. **f** Mitosis annotations were transferred onto H&E WSIs for further analysis (each dot represents a mitosis annotation)

A two-step image transform approach was used to align PHH3 and H&E whole slide images (WSIs) to assist pathologists in verifying mitoses on H&E. All image alignment procedures were performed on PHH3 WSIs, while the H&E slides remained unaltered. Of the 22 pairs of WSIs, the H&E WSI from one pair was mainly out-of-focus, and two pairs failed to align the PHH3, leaving the remaining 19 WSIs for further annotation (Fig. 1(a)).

H&E and PHH3 image tiles with a size of one HPF (size=$1600\times 1600$ pixels, or $0.4mm \times 0.4mm = 0.16mm^2$)^1^ were extracted from WSIs without overlapping (Fig. 1(b)). Two postgraduate year 3 (i.e., three years in training) pathology residents independently annotated the mitoses in each HPF using a web-based interface (Fig. 1(c-d)), which can show H&E and PHH3 HPFs side-by-side. The resident-annotated images were then reviewed by a neuropathologist (M.H.) with the same annotation interface. The neuropathologist provided the final judgment on the mitosis annotation using the criteria specified in Additional file 1, Section 1 (Fig. 1(e)). The resulting annotations were transferred to H&E WSIs (Fig. 1f) and were used for the MC analysis.

### Quantification of the hotspot mitosis count of WSIs

In each of the 19 annotated WSIs from Sect. 2.1, the MC in 10 HPFs was quantified with the following six different methods. Methods 1 – 3 count MCs in consecutive 10 HPFs and are compatible with the current definition of WHO CNS 5 Blue Book, while the HPFs in methods 4 – 6 are not necessarily connected. Background and out-of-focus regions were identified by a deep-learning algorithm [37] and were excluded. Non-tumor regions were marked by a neuropathologist (M.H.) with Aperio Imagescope software (version 12.3.2.8013)^2^ and were not used for calculation.

1. *MC in 10 HPFs generated by depth-first search algorithm (Proposed)* This method arranges HPFs as a sequence of 10 connected HPFs that form a *connected* path. Because the HPF view in digital pathology is square, two HPFs that share at least one connected edge are considered connected. The path should be traversed from the first HPF to the tenth, with the requirement that each HPF is visited exactly once. The path is calculated by our proposed DFS algorithm (see Appendix A for implementation), which consists of a recursive depth-first searching with backtracking. In a WSI, the algorithm comprehensively searches all possibilities of 10 connected HPF paths, satisfying the constraint mentioned above to guarantee that the resulting quantification of MC is the highest, mathematically.
2. *MC in linear 10 HPFs* This method quantifies the highest MC of 10 HPFs with the linear arrangement. The highest MCs in $10\times 1$ HPFs (vertical) and $1\times 10$ HPFs (horizontal) are reported.
3. *MC in rectangular 10 HPFs* This method follows the previously reported algorithm [26] and quantifies the highest MC of 10 HPFs with the rectangular arrangement. The highest MCs in $5\times 2$ HPFs (vertical) and $2\times 5$ HPFs (horizontal) are reported.
4. *Average MC per 10 HPFs* This method quantifies the average number by dividing the total MC in tumor areas by the size of the tumors and multiplying by 10.
5. *MC in random 10 HPFs* This method [7] randomly samples 10 HPFs from each WSI 1,000 times (without replacement). The distribution of MCs in each set of 10 sampled HPFs is reported.
6. *MC in maximum 10 (not connected) HPFs* The MC of each HPF in the entire WSI is ranked in descending order, and the MC of the top 10 HPFs is added together. This method represents the maximum possible MC that can be observed in 10 HPFs within a WSI.

Furthermore, the MCs yielded by methods 1 – 3 were compared to the WHO grades determined by the consensus conferences. Here, we sought cases where *upgrades* in WHO grades could be according to the criterion of the MC alone.

### User study for evaluating pathologists’ mitosis detection

A user study was conducted to evaluate the ability of pathologists and pathology residents to detect mitoses in meningioma at the magnification comparable to 1 HPF. The study was approved by the Institutional Review Board of the University of California, Los Angeles (IRB#21-000139) and was conducted between February 2023 and May 2023.

**Fig. 2:**
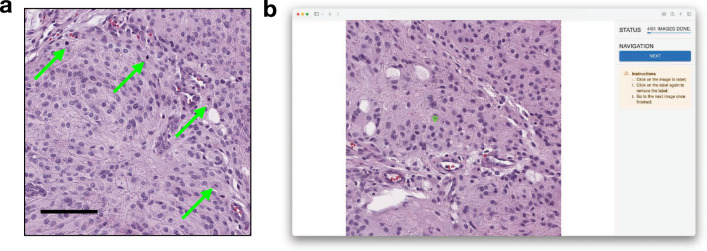
**a** Representative HPF image used in the study, with mitoses noted by arrows, bar=100$\mu m$. **b** Interface screenshot of the user study website, where the HPF images were shown to participants for mitosis annotation in the random order

Forty-eight images, each equal to 1 HPF surface area, were selected by a neuropathologist (M.H.) from two H&E WSIs of meningothelial-transitional meningioma for each case (see Fig. 2(a) for example), based on five criteria (see Additional file 1, Section 2). Random sampling was not used because approximately 89.1% of the area in the two WSIs did not include mitosis. The decision to limit the number of images to 48 was motivated by practical considerations: our preliminary finding suggested that annotating these 48 HPF images required about 30 min for a pathologist, and increasing the number further would not have been feasible due to the potential risk of causing fatigue among the participants.

Amongst 48 selected HPF images, eight did not include mitoses, while the remaining images had 88 mitoses, ranging from one to six per image. Note that the image set was skewed towards HPFs with one or more mitoses to observe pathologists’ correctness when detecting mitoses with various morphologies.

The participants were recruited by sending email invitations to the mailing list and snowball recruitment. In total, 41 participants from 11 institutions (Appendix B) in the United States (N=40) and Costa Rica submitted their responses, including 19 neuropathologists/neuropathology fellows (AP/NP), 10 pathologists/pathology fellows (AP), and 12 pathology residents.

Each participant was instructed to visit our user-study website with their own laptop or desktop computer with recommended settings. On the website, participants watched an instruction video and were asked to answer multiple-choice questions about their occupation and sub-specialty (if applicable). Afterward, they reviewed three tutorial images to become familiar with the website interface and the task. Participants were informed that there may be zero to six mitoses in each of the 48 images and were instructed to annotate mitotic figures based on their daily practice experience. The 48 images were presented to each participant in random order (Fig. 2b). To prevent bias, participants were blinded to the mitosis annotations until they completed the study. Their annotations, survey responses, and time logs were recorded for further analysis.

### Grouping pathologists’ annotations

**Fig. 3:**
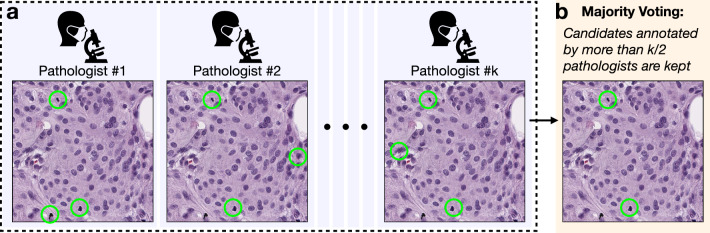
Generating mitosis decisions with grouping annotations from a set of *k* pathologists. **a** Step 1 (group member selection): annotations were collected from an odd number of *k* pathologists. The *k* pathologists are selected randomly. **b** Step 2 (majority voting): candidates annotated by more than *k*/2 pathologists are kept as the final grouping mitosis decision

The grouping process is based on the participants’ annotations collected in Sect. 2.3: First, annotations from an odd number of *k* pathologists were randomly selected (Fig. 3(a)). Second, mitosis candidates that were annotated by more than *k*/2 (i.e., $>50\%$) participants were kept as the decision of the group (Fig. 3(b)). This approach assigns equal importance weight to each selected participant and emulates a majority voting process. As for the group size, we randomly selected from the 41 participants without replacement and explored the group size from 3 to 37 (i.e., 3,5,7,9,..., 37). For each group size, we ran the grouping process 100 times for result evaluation.

### Measurements and statistics

The correctness of mitosis detection was measured by precision or positive predictive value (PPV, see Eq. 1) and sensitivity (recall, see Eq. 2), which was calculated according to the true-positive (TP), false-positive (FP), and false-negative (FN) counts. A TP indicates a mitosis present within 15$\mu m$ (60 pixels) distance of a participant’s annotation. An FP stands for no mitoses present within 15$\mu m$ radius distance of a participant’s annotation. And an FN means no participant’s annotations present within 15$\mu m$ radius distance of a mitosis.

We report the precision and sensitivity of three conditions: (1) individual participant; (2) decisions from groups that have sizes between 3 to 37; and, (3) an EfficientNet-b3 Convolutional Neural Network AI model [38, 39] trained from a part of mitosis annotations collected in Sect. 2.1 (see Additional file 1, Section 3 for the training detail), as a reference. A bootstrapping method (10,000 times, 100% re-sampling with replacement) was used to describe the average and 95% confidence interval for these criteria. For AI evaluation, we ran the AI on the 48 HPF images 100 times. In each test run, these HPF images were randomly flipped and/or rotated ($0^{\circ }, 90^{\circ }, 180^{\circ }, 270^{\circ }$), and the Dropout layers in the AI model were enabled.

1 $$\begin{aligned} Precision (PPV)= & {} \frac{TP}{TP + FP} \end{aligned}$$

2 $$\begin{aligned} Sensitivity~(Recall)= & {} \frac{TP}{TP + FN} \end{aligned}$$

We further tested whether participants’ precision and sensitivity varied according to their experience level: a Kruskal-Wallis Test was applied to test significance among three of the participants’ experience levels (i.e., AP/NP, AP, and pathology residents). A post-hoc Dunn’s test was used to show pair-wise difference between each experience level.

Finally, we introduced a metric called *agreement rate* as a measure of participants’ consistency in detecting mitoses: Given a nucleus, its agreement rate was defined as the percentage of participants that annotated it. We calculated the agreement rates for (1) all “ground truth” mitoses (i.e., annotated by the participants in Sect. 2.1), and (2) false-positive mitoses where *more than three* participants agreed on to illustrate false-positive errors with higher agreement rates.

The image processing, AI inferencing, and statistics were performed in a local server with Intel W-2195 CPU with 128GB RAM and Nvidia RTX 3090 Graphics Processing Unit. The server has a Python 3.6.8 environment, with Numpy version 1.19.5, OpenCV version 4.5.2, scikit-image version 0.17.2, scipy version 1.7.3, scikit-learn version 1.0.2, PyTorch 1.12.0, and Matplotlib version 3.5.2 for figure making.

## Results

### Mitosis count in meningioma WSIs

**Fig. 4:**
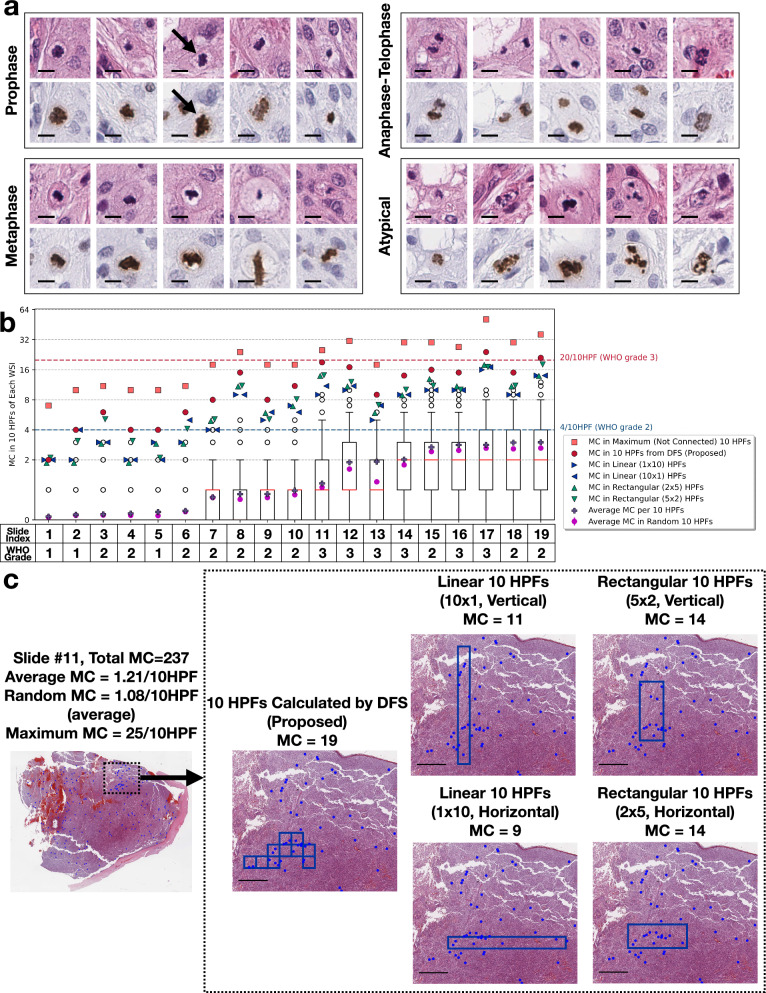
**a** Example of annotated mitoses in the meningioma WSIs at different phases, including prophase, metaphase, anaphase-telophase, and atypical mitoses, bar=10$\mu m$. **b** Hotspot MC per 10 HPFs in each WSI using five methods of quantification, namely the proposed DFS algorithm, linear 10 HPFs (i.e., $10\times 1$ and $1\times 10$ arrangements), rectangular 10 HPFs (i.e., $5\times 2$ and $2\times 5$ arrangements), average MC per 10 HPFs, and maximum possible MC in 10 (not connected) HPFs.  For the MC distribution in randomly-sampled 10 HPFs: the box-whisker plot shows the percentiles and medians, and the magenta error bars demonstrate the average and 95% confidence intervals. For each WSI, the WHO grade assigned at the consensus conference was included. **c** Mitosis distribution in slide #11, and the spatial distribution of 10 HPFs with the proposed DFS algorithm (MC=19), linear 10 HPFs (MC=9 or 11), and rectangular 10 HPFs (MC=14), bar=1*mm*

A total of 4,133 mitotic figures were annotated with examples shown in Fig. 4(a). The 19 WSIs exhibit a wide range of total MCs, spanning from 8 to 623 per slide. Figure 4(b) presents the hotspot MCs of each WSI, quantified according to the six methods introduced in Sect. 2.2. The MCs quantified by the DFS are generally higher than those with rectangular or linear HPF arrangements: On average, DFS counted 4.29 and 3.32 additional mitoses than those with linear and rectangular HPF arrangements, respectively.

Importantly, our proposed DFS algorithm identified two borderline cases where the meningioma grades may be higher than the original WHO grades from the consensus conferences, based on the MC alone. Slide #5, previously signed-out as WHO grade 1 meningioma, could be upgraded to grade 2 due to the presence of 4 mitoses in the DFS-counted 10 consecutive HPFs. Slide #19 was signed-out as WHO grade 2 meningioma, but could be upgraded to grade 3 based on the DFS finding of 21 mitoses/10HPFs.

For all 19 WSIs, the MCs in maximum 10 (not connected) HPFs exceed the WHO grade 1/2 threshold. The MCs in the randomly selected 10 HPFs (average=1.18 mitoses/10HPFs) are approximately 16.1% lower than the average MC/10HPFs (average=1.37 mitoses/10HPFs), which indicates these two metrics are not always aligned.

Figure 4(c) displays the spatial distribution of mitosis locations in one example WSI (slide #11). It also shows the hotspot 10 HPFs identified by the DFS algorithm, and with linear/rectangular arrangements exhibiting roughly similar locations. Nonetheless, the MCs in these areas significantly differ due to the variation in HPF arrangements: the MC in the DFS-calculated HPFs was 19. However, the rectangular arrangement had 14, and the linear arrangement only had 9 or 11.

### Participants’ correctness of detecting mitoses

**Fig. 5:**
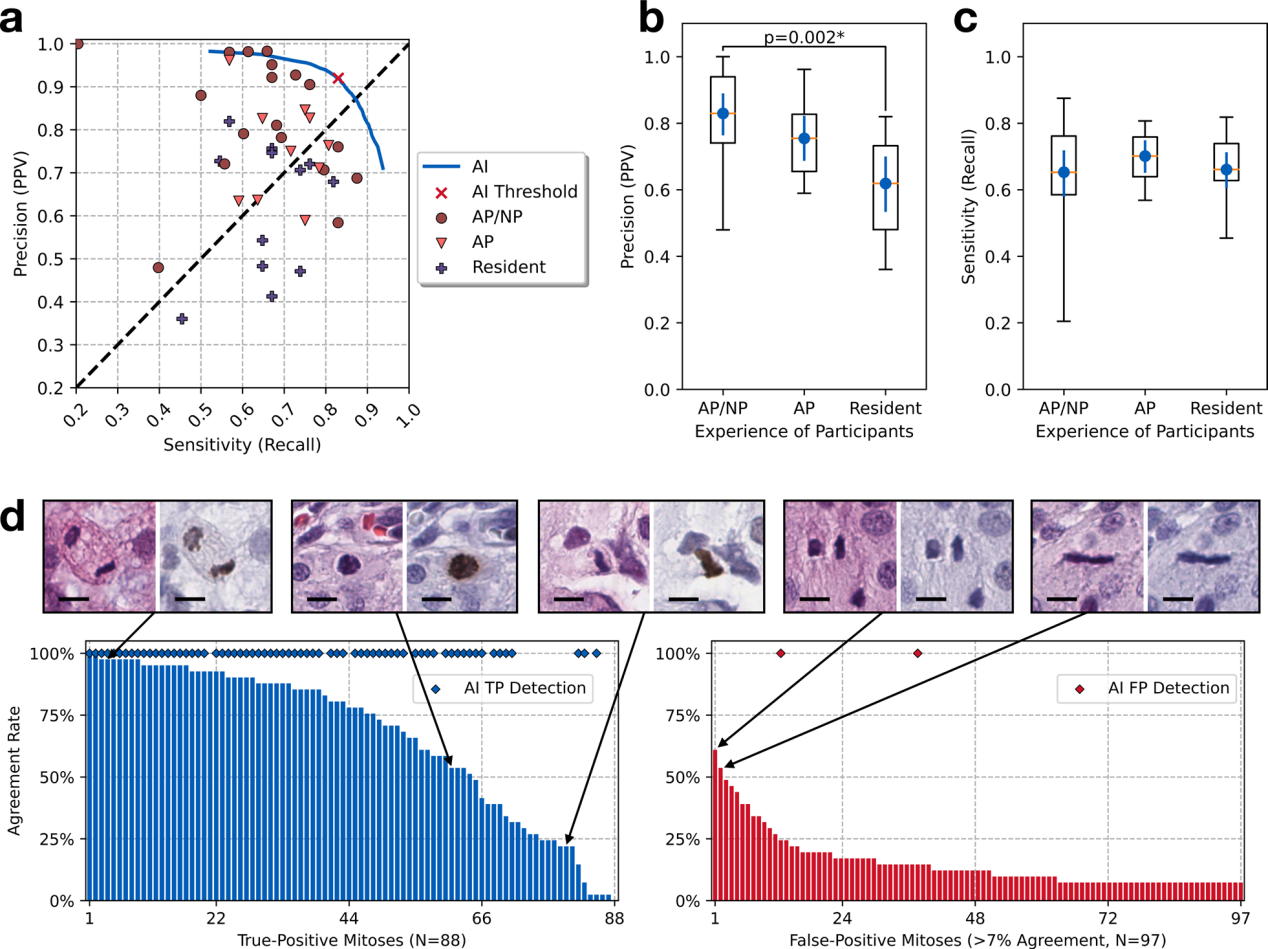
**a** The precision-recall scatter plot for each participant’s performance in detecting mitoses on the 48 HPF images from the user study. The blue line indicates the precision-recall curve from AI and the marker ($\times$) indicates AI’s operating point with the threshold cut-off. The diagonal dashed line is the reference where the precision is equal to sensitivity. **b** The box-whisker plot with the average and 95% confidence interval, showing the precision (PPV) values of the participant groups with different experience levels: AP/NP, AP, and pathology residents. AP/NP participants achieved significantly higher precision than pathology residents (p=0.002, post-hoc Dunn’s test). **c** Box-whisker plot with 95% confidence intervals, showing the sensitivity (recall) values of the participant groups with different experience levels. No statistical significance was observed. **d** Bar plots illustrating the agreement rates of participants in identifying ground-truth mitoses and false-positive mitoses, with selected examples (bar=10$\mu m$). The AI detections (both true-positive and false-positive) are shown as the diamond ($\lozenge$) markers

Overall, the 41 participants achieved an average precision of 0.750 (Standard Deviation (*SD*) 0.026, 95% Confidence Interval (*CI95*) [0.698, 0.800]), sensitivity 0.667 (*SD* 0.002, *CI95* [0.626, 0.704]). Among the participants, the top-2^3^ were a neuropathologist and a neuropathology fellow, achieving a tied score with a precision of 0.905 and a sensitivity of 0.761 (TP: 67, FP: 7, FN: 21). In contrast, the AI model achieved an average precision 0.920 (*SD* 0.002, *CI95* [0.916, 0.923]) and sensitivity 0.830 (*SD* 0.002, *CI95* [0.826, 0.833]). As shown in Fig. 5(a), the AI model exhibits both high precision and sensitivity, resulting a performance better than human participants.

Participants made significantly fewer false-positive mistakes than false-negative ones ($p=0.012$, effect size $r=0.277$, Wilcoxon rank sum test), indicating that they tended to achieve a higher precision than a higher sensitivity: As shown in Fig. 5(a), 26 out of 41 participants achieved a higher precision than sensitivity (above the diagonal dashed line), while 15 out of 41 participants achieved a higher sensitivity instead (below the diagonal dashed line).

A Kruskal-Wallis test revealed a significant difference in participants’ precision ($p = 0.003$, $\eta _H^2 = 0.231$) according to their experience level. Post-hoc Dunn’s test indicated a significant difference ($p = 0.002$) in the precision between AP/NP participants and pathology residents (Fig. 5(b)). The average precision of the former group was 0.829 (*SD* 0.033, *CI95* [0.763, 0.890]), while the latter had 0.619 (*SD* 0.043, *CI95* [0.531, 0.701]), indicating a 33.93% increase. No significance was observed among other comparison pairs. As for the sensitivity (Fig. 5(c)), the Kruskal-Wallis test did not show a significance due to participants’ experience level ($p = 0.715$).

Figure 5(d) shows the distribution of participants’ agreement rates for 88 ground-truth and 97 false-positive mitoses that at least three participants had agreed on. Among the ground-truth mitoses, 64 out of 88 had agreement rates exceeding 50%, while only 2 out of 97 false-positive mitoses achieved agreement rates above 50%. This provides motivations to use the majority voting approach to reduce false-positive errors.

### Grouping pathologists’ decisions and evaluation of correctness

**Fig. 6:**
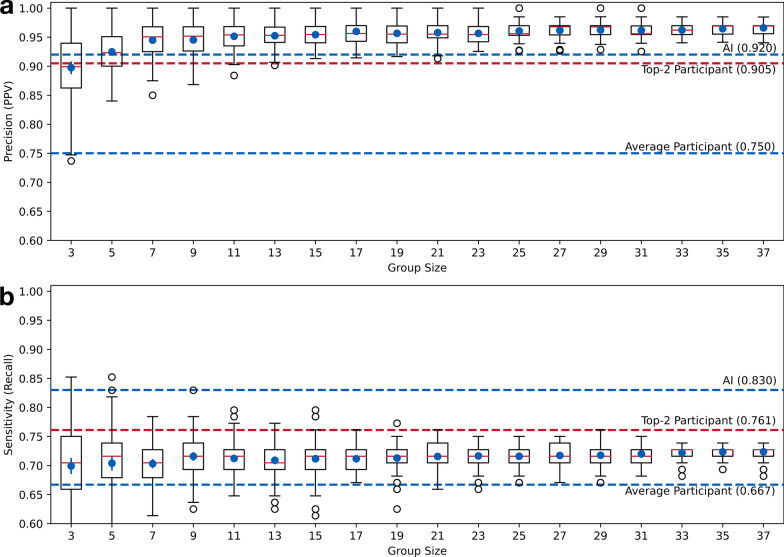
Box-whisker plot with the average and 95% confidence interval for **a** precision and **b** sensitivity values of decisions derived from grouped participants

Figure 6(a) presents the precision of decisions of groups sized between 3 and 37. The figure demonstrates that the grouping process can effectively mitigate random errors made by individual pathologists: Even groups of three participants achieved an average precision of 0.897 (*SD* 0.006, *CI95* [0.886, 0.908]). Compared to the precision an average participant (0.750), the groups of three participants demonstrated a 19.60% increase. Groups of five participants achieved an average precision of 0.925 (*SD* 0.004, *CI95* [0.918, 0.932]), which was slightly higher than the AI model (0.920, 0.54% increase), and the top-2 individual participants (0.905, 2.21% increase). Groups of more than seven participants would have a higher precision in general, ranging from 0.945 (for groups of 7) to 0.966 (for groups of 37).

The average sensitivity of groups of three participants was 0.699 (*SD* 0.007, *CI95* [0.685, 0.713]), which was also higher than an average participant (0.667, 4.79% increase). Figure 6(b) shows the sensitivity scores for groups of varying sizes, ranging from an average of 0.699 (for groups of 3) to 0.724 (for groups of 37). The sensitivities of grouped participants are lower the top-2 participants (0.761) and the AI model (0.830) in general.

Interestingly, the peak sensitivity of 0.852 was reached by a specific group of three participants, who also had a precision of 0.862. However, out of the 100 grouping experiments that involved sampling three participants, we observed only two groups that outperformed AI in terms of sensitivity.

## Discussion

Mitosis is an important component in grading many brain tumors such as meningioma, IDH-mutant astrocytoma, oligodendroglioma, solitary fibrous tumor and ependymoma among others [2]. Additionally, mitosis can provide clues to modify a differential diagnosis toward a more accurate diagnosis. For instance, a subependymoma with few mitoses may prompt more careful examination for including an ependymoma-subependymoma in the differential [40]. Few scattered mitoses in a high-grade appearing IDH-wild type astrocytic tumor should add pleomorphic xanthoastrocytoma to the differential diagnosis beside glioblastoma usually demonstrating brisk mitotic activity [2]. Accurate mitosis quantification and detection is important but time-consuming – it can be challenging especially in large and heterogeneous specimens, where few highly proliferative foci, present in only one slide, are easily missed.

To our best knowledge, this is the first study to comprehensively analyze MC quantification approaches and observe the “grading migration” [41] through a new computer algorithm for mitoses counting. However, it should be underscored that the aim of the study is not highlighting the errors of pathologists. Instead, this work demonstrates the potential of using computer algorithms to precisely quantify MCs following the WHO guidelines. The algorithm can offer more flexibility in arranging HPFs, which may be challenging for human pathologists, even in digital interfaces. Furthermore, this study is also unique in its evaluation of pathologists’ ability for mitosis detection with the largest number of participants so far, compensating for previous studies that had limited participants and highlighted the inter-pathologist variation in detecting mitotic events events [4, 24]. Because of its large participant size, this work demonstrates that a higher precision and more robust mitosis detection can actually be achieved by the majority voting from a group of pathologists.

While new molecular methods such as chromosomal microarray analysis [42], next generation sequencing [43], and methylation profiling [20] provide better understanding of tumor biology and behavior, they are time-consuming, costly and unavailable in many countries [44]. This technology gap generates an urgent need for developing AI-assisted solutions to analyze the mitotic count based on the histologic analysis. The AI pathology assistant is instantly present, cost-effective, and available 24/7, especially for institutions where trained neuropathologists are less available. In this study, for example, the average time for pathologists to annotate the 48 HPF images was about 24 min 32 s ($SD=91.63$ seconds, $CI_{95}$=[21 min 56 s, 27 min 55 s]). In contrast, it only took about 1 min 33 s ($SD=6.08$ seconds, $CI_{95}$=[1 min 24 s, 1 min 41 s]) for a computer (see Sect. 2.5 for hardware configuration) to finish the mitosis detection by AI. It is noteworthy that such computer speed can be further accelerated by parallel computing, where clusters of computers split the task and work individually in parallel. In the future, the AI shall examine the entire set of slides, detect almost all mitotic figures, and generate a detailed report showing mitosis hotspots.

Since 2012, a considerable amount of literature has built mitosis datasets for AI development [30–34, 45], although there is still a lack of mitosis datasets in meningiomas. A widely-adopted approach for mitosis-detection AI includes two steps: an initial AI model screening potential candidates, followed by a step-2 for verification by AI [30, 31, 46, 47]. This work follows this approach and introduces the first mitosis AI specifically trained based on mitoses in meningiomas, and compares the correctness of mitosis detection between humans and AI though a user study of 41 participants. We discovered that the AI could outperform pathologists in sensitivity. Although few pathologists achieved higher precision, AI maintained high precision and sensitivity simultaneously, which contributed to a higher overall performance.

 Although AI has demonstrated impressive performance, human-based quality assurance must be introduced: mitotic figures identified by AI still need to be checked for accuracy, especially in substandard samples with cautery and other processing artifacts. To demonstrate this workflow of human pathologists’ supervision of AI, we have designed and implemented a prototype that can integrate the computational approach proposed in this study.

**Fig. 7:**
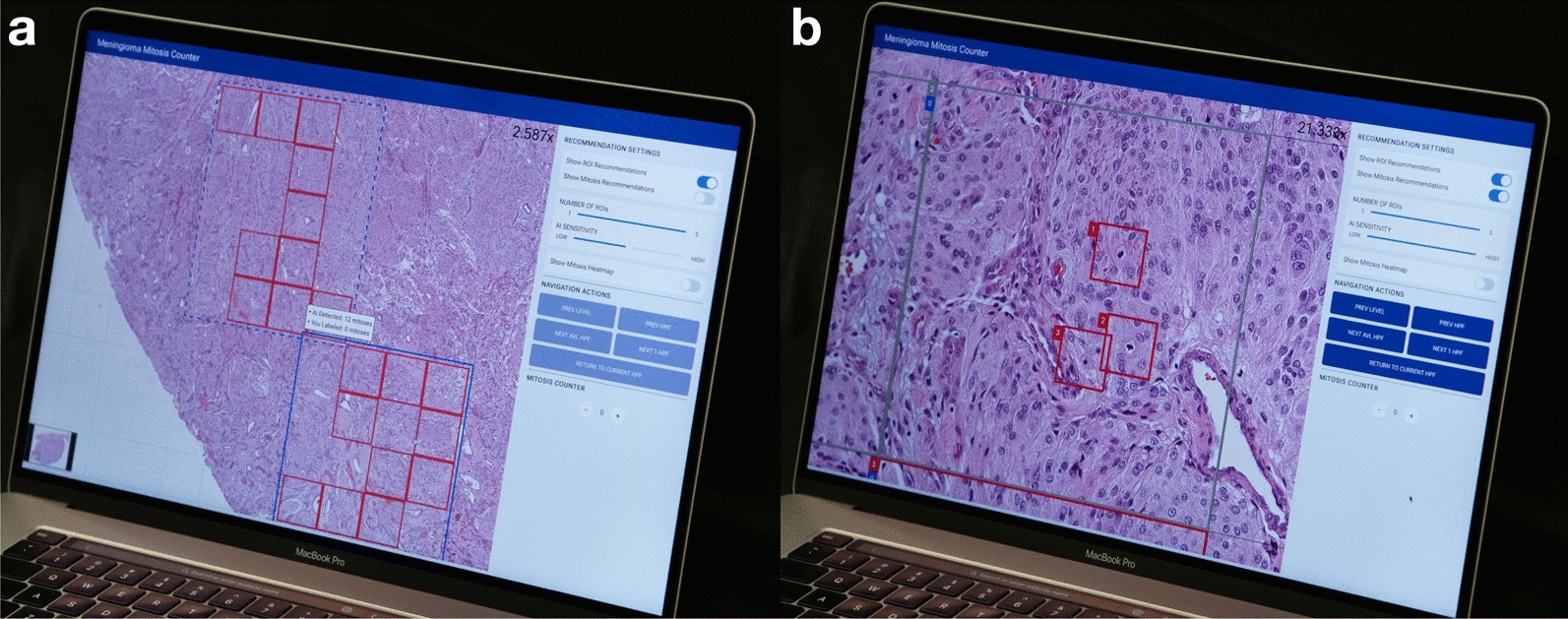
An AI-assisted prototype that conceptualizes the augmented pathology. **a** DFS-quantified mitosis hotspots are calculated based on AI-detected mitoses according to a sensitivity setting. The user can adjust the number of hotspots and adjust the AI sensitivity with the sliders on the right side of the interface. **b** Once a user selects a DFS-quantified hotspot, the prototype can guide the user to see each HPF inside, where it can mark a box surrounding each AI-detected mitosis candidate with the highest sensitivity. Multiple pathologists may visit the same hotspot areas recommended by the prototype to ensure the highest precision

Figure 7(a) illustrates the prototype’s functionality, where users can activate the “Show ROI Recommendation” (ROI: region of interest) feature to visualize hotspot areas quantified by the DFS algorithm. Users have control over the number of DFS-quantified hotspots displayed (ranging from 1 to 5) as well as the sensitivity setting of AI. Upon selecting a DFS-calculated hotspot for further examination, the system guides the user to examine each HPF within the hotspot at higher magnification (Fig. 7(b)), with AI-detected mitoses boxed by the system. The prototype assists pathologists in maintaining consistency in the selection of hotspot areas found by the DFS algorithm and also allows them to see potential mitoses marked by AI in the high-sensitivity setting. To achieve a high precision, one can let a group of pathologists examine the slide with the prototype independently. Therefore, the combined high sensitivity by AI and high precision by pathologists can ultimately achieve a higher overall performance. Even with AI as an assistant, it is the human pathologist who provides the adjudication for each mitosis, and mechanisms to prevent humans from blindly trusting AI should be introduced and evaluated in the future.

### Limitations and future work

This work covers two aspects of mitosis quantification and mitosis detection. This subsection discusses the limitations in each aspect and suggests potential future directions for improvement.

For the experiment of the MC quantification:

1. *Cohort size* This study could have benefited by including other independent cohorts from different institutions, which need more resources not available at this time. To potentially address this, we extended the validation of the DFS algorithm to two additional public datasets with full-slide mitoses annotations in canine mammary carcinoma [31] and canine cutaneous mast cell tumors [30]. In both datasets, the DFS algorithm consistently demonstrated an ability to yield higher MCs compared to linear ($\sim$13 more) and rectangular ($\sim$8 more) HPF arrangements (see, Additional file 1 Section 6). While these results should not be directly compared to meningiomas due to varying mitosis prevalence rates across different tumor types, this preliminary finding sheds light on the potential of the DFS algorithm to enhance the MC quantification in various tumor contexts, providing a foundation for future large-cohort studies.
2.  *Selection of slides* In this study, only one to two slides were sampled from each patient, which could inevitably introduce bias to the MC quantification and subsequent grading analyses, because other slides might have a higher mitotic rate. Ideally, at least one tissue section/block/slide is recommended for every tumor cm. Therefore, future studies can include entire set of slides from each patient to ensure a comprehensive analysis.

For the user study evaluating the mitosis detection:

1. *Limited test images* The use of only 48 selected HPF images in the user study introduces potential biases and may influence the findings, and the duration of the study is relatively brief (about 30 min in total). Since pathologists are used to the H&E stain in their own institutions, they usually need more time to get used to H&E slides from another institution with different performance. Therefore, future work can focus on measuring pathologists’ mitosis identification with more images from multi-z-axes WSIs and standardized H&E stains.
2. *One z-plane in WSI scanning* All specimens were scanned with only one z-plane instead of multiple, due to the limitation of instrumentation. In light microscopy, pathologists can adjust the z-focus, which can aid in judging the authenticity of a mitosis. Hence, the missing z-axis might cause pathologists to achieve a lower sensitivity, which, unfortunately, makes the sensitivity values reported in this work inevitably speculative.
3.  *Generalizability of AI* The AI used in this study was developed based on a single meningioma dataset, and the bench-marking of its performance on other tumor datasets is, unfortunately, out of the scope of this study and thus considered as future work.
4. *Participant selection* There was also a potential bias in the participants, because the majority of participants are from the United States. To address this, we have provided public access to the user study website (https://mg-labeler.vercel.app) and encourage readers of interest to participate.

## Conclusion

This work presents a computational strategy that leverages digital pathology to enhance the quantification and detection of mitosis. The strategy consists of two key components: (1) a depth-first search algorithm that quantifies the mathematical maximum mitosis count in 10 consecutive HPFs, enabling more precise grading, especially in cases with borderline mitotic figures; and (2) a collaborative approach that groups pathologists to detect mitoses under each HPF, reducing random errors from individual assessments and thus increasing assessment robustness. The integration of our computational strategy with AI promises a more efficient and robust mitosis assessment by pathologists, which holds the potential to create more accurate reports when mitoses count is critical for tumor grading, and ultimately, benefits patient management.

### Supplementary Information

**Additional file 1:** Summary of supplementary documents.

**Additional file 2:** De-identified information sheet of mitosis count summary for each WSI.

**Additional file 3:** Mitosis count in 10 HPFs with the DFS, linear, and rectangular arrangements, with regard to the shift in the coordinate origins.

**Additional file 4:** Mitosis annotations (ground truth) of the 48 HPF images in the user study.

**Additional file 5:** Performance report of a typical AI prediction on the 48 HPF images.

**Additional file 6:** Performance report of the top-2 participants that achieved the best performance in the user study (1/2).

**Additional file 7:** Performance report of the top-2 participants that achieved the best performance in the user study (2/2).

## Figures and Tables

**Algorithm 1:**
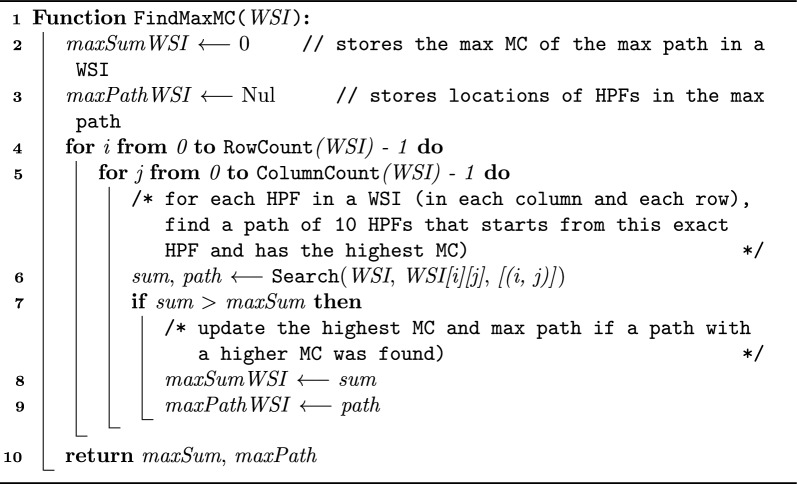
Find the max MC in 10 consecutive HPFs of a WSI

**Algorithm 2:**
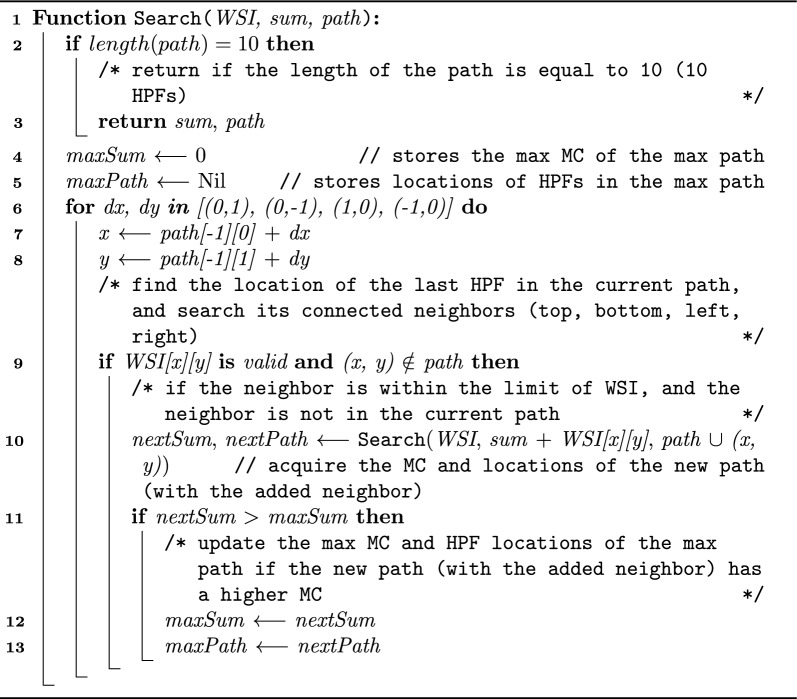
Depth-first search helper function

## Data Availability

The user study website is can be accessed publicly online. The mitoses annotations of the images used in the user study, a summary information sheet of mitosis count for each Whole Slide Image, reports of the AI’s and top-2 participants’ performance in the user study are also submitted as the supplementary material. Other raw data generated from the study are available from the corresponding authors upon reasonable request.

## Appendix A depth-first search algorithm to calculate sum of mitosis count in highest 10 consecutive HPFs

Locations of all mitosis annotations (whether they were annotated by pathologists or detected by AI) of a WSI are needed in order to run the DFS algorithm. Firstly, the WSI was split into non-overlapping HPF tiles. Then, a matrix of MC for each HPF in the WSI, denoted as *WSI*, was built. The coordinate of each element in *WSI* matrix represents the location of a HPF, and the value represents the MC in this HPF. The algorithm FindMaxMC is the main function, and algorithm Search is the helper function. To caulcate with DFS, the *WSI* matrix should be passed to FindMaxMC function, and it will return two values: (1) *maxSum*: the highest MC in 10 consecutive HPFs, and (2) *maxPath*: the locations of these 10 HPFs.

## Appendix B summary of participants’ affiliations

See Table 1

**Table 1:** Participants’ affiliations in the user study

Institution	Number of Participants
Kansas University Medical Center	18
UCLA / Ronald Reagan UCLA Medical Center	6
Baylor College of Medicine / Texas Children’s	6
Brown University / Rhode Island Hospital	3
University of California, San Francisco	1
Mayo Clinic	1
University of Pennsylvania / Penn Medicine	1
USC / Los Angeles General Medical Center	1
Loma Linda University	1
UTHealth Houston	1
Other (Unspecified / not in United States)	2
Total	41

## Abbreviations

CNS: Central nervous system
WHO: World Health Organization
WHO CNS 5 Blue Book: The 2021 WHO classification of tumors of the CNS (5th edition)
MC: Mitotic count
IHC: Immunohistochemistry
PHH3: Phosphohistone-H3
AI: Artificial intelligence
WSI: Whole slide image
HPF: High-power field
DFS: Depth-first search
AP/NP: Neuropathologists/neuropathology fellows
AP: Pathologists/pathology fellow
TP: True-positive
FP: False-positive
FN: False-negative
SD: Standard deviation
CI95: 95% Confidence interval
ROI: Region of interest

## Footnotes

1 This work defines an HPF as a square. All HPFs mentioned in this work had sizes of $0.16mm^2$, as defined in the 2021 WHO CNS 5 Blue Book [2]. Therefore, the edge of an HPF was 0.4*mm*, or 1,600 pixels ($0.25\mu m$/pixel for 400$\times$ scans).

2 https://www.leicabiosystems.com/us/digital-pathology/manage/aperio-imagescope/

3 The top-2 is defined by the F1 score, which is the geometry mean of precision and sensitivity.
