# Supplementary material for: Enhancing mitosis quantification and detection in meningiomas with computational digital pathology
Source: Acta Neuropathol Commun. 2024 Jan 11;12:7. doi: 10.1186/s40478-023-01707-6 (PMC10782692; doi:10.1186/s40478-023-01707-6)
Supplement: Supplementary file 1 — Additional file 1: Summary of supplementary documents. [file 40478_2023_1707_MOESM1_ESM.pdf]

Supplementary Material Summary

1 Criteria for Determining Mitosis

Precedence was given to the H&E slides to confirm a mitotic figure while the PHH3 was used as a guide and assistance tool. The following criteria were followed.

1. Highly confident mitotic figures were determined when the H&E-stained slide showed a classic mitotic figure at prophase, metaphase or anaphase-telophase, and the PHH3 was positive. Also confirmed when a classic mitotic figure detected in H&E slides but it was weak or negative in PHH3 immunostain (our previous studies showed that some mitotic figures are negative for PHH3 due to variable antibody sensitivity or poor formalin fixation and/or tissue processing issues. In some cases the repeat immunostain was positive, data not shown).
2. When H&E supported or favored a mitotic figure and PHH3 supported this decision, the mitotic figure was confirmed.
3. When H&E did not highly favor a mitotic figure but the PHH3 was positive, the PHH3 was carefully examined for the appearance of a classic mitotic figure at various stages and the H&E was re-examined; if the morphology of the potential mitotic figure matched the classic pattern of mitosis seen in PHH3 immunostain, the mitosis was confirmed, otherwise it was rejected.
4. When the H&E did not favor a mitotic figure and the PHH3 was negative, mitosis was rejected.

5. When a classic pattern of mitosis was seen in the PHH3 immunostain, but this pattern was not seen in the H&E slide, the mitosis was rejected. Our decision for rejecting this kind of mitosis is to prioritize H&E to simulate the practice of pathology in the real world. It is noteworthy that these are true mitotic figures showing classic morphology on PHH3 immunostain but were not included as the mitosis morphology was not evident on H&E.

2 Criteria for Selecting HPFs for the User Study

The following criteria were used to select the 48 HPFs for the user study.

1. HPFs with no or minimal out-of-focus area.
2. HPFs with at least 50% tumor content.
3. Mitotic figures at different stages (i.e., prophase, metaphase, and anaphase-telophase) should be present.
4. Very few atypical mitotic figures in the collection are allowed.
5. The number of mitotic figures ranges from none to 6 to represent mitotic counts that may be seen in tumor grades 1 – 3. HPFs with significant staining issues and tissue folding/wrinkling artifacts were removed from the collection.

3 AI Training Detail & WSI-Level Evaluation Result

An EfficientNet-b3 Convolutional Neural Network (CNN) [1] was trained based on H&E WSIs and their mitosis annotations, with 10/19 slides for training/validation, and 9/19 for testing. The upper 75% areas of the training WSIs were used for training, leaving the lower 25% for validation and hyperparameter tuning.

The model training process involves a multi-round activate learning strategy: for each round, both training and evaluation sets consists of patches (size=240×240) extracted from the corresponding areas in the WSIs. In each round, the model was

trained with Stochastic Gradient Descent optimizer (momentum=0.9), Cosine Annealing learning rate scheduler with warm restart (max learning rate=6×10^{-4}), with combined online uncertainty sample mining (k=1) [2] and Consistent Rank Logits loss [3], batch size 128, data augmentation as specified in [4], and 80 epochs. After each round of training, the model with best validation F1 score was applied to the train WSIs. Correspondingly, false-positive and false-negative patches were added to the training/validation set for the next round of training. The training process repeated four times when the validation F1 score stopped increasing, resulting 102,575 patches in the training set and 24,792 in the validation. The model with the best validation F1 score in the last round of training, along with corresponding thresholds, was chosen for evaluation.

For evaluation, the trained CNN was applied on the test WSIs with a window size of 240×240 pixels and a step size of 45 pixels. A window with a probability greater than 0.70 was regarded positive, and overlapping positive windows were removed by non-max suppression with a threshold of 0.10. For each remaining positive window, a class-activation map was calculated using GradCAM++ [5], and the centroid of each hotspot in the class-activation map was extracted as a candidate mitosis location. The CNN was applied again on these candidate locations for step-2 verification, and candidates with a step-2 verification probability greater than 0.775 were considered as positive mitosis detections.

Figure 1 shows the precision-recall curve of the CNN on the nine test WSIs. With the threshold cut-off of 0.775, the model achieved a 1,091 TP, 437 FP, and 491 FN mitoses, resulting a precision/PPV of 0.714, sensitivity/recall of 0.690, and an F1 score of 0.702.

A local server with Intel W-2195 CPU (36 cores), 128GB RAM and Nvidia RTX-3090 GPU was used for model development and performance evaluation. All model training and evaluation were performed in a Python 3.7.13 environment, with Melas

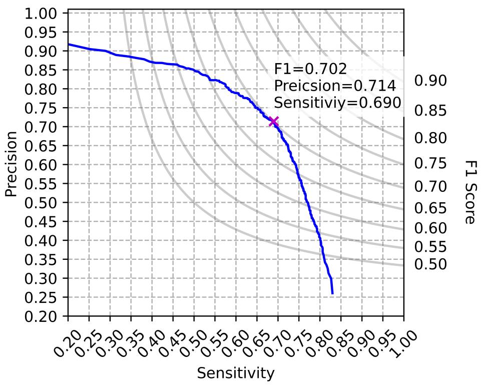

Fig. 1: Precision-recall curve for the AI model on the nine test WSIs.

et al. 's implementation of EfficientNet with ImageNet pre-trained weight[1], and Gildenblat et al.'s implementation of GradCAM++[2].

4 Robustness of DFS against Shifting in Coordinate Origins

To further assess the robustness of DFS algorithm against shifts (i.e., the repositioning of HPFs due to the change in coordinate origins), we displaced the coordinate origins of all mitosis annotations in three directions and calculated the updated MCs for DFS, linear (1×10 and 10×1 HPFs), and rectangular (2×5 and 5×2 HPFs). These three directions include:

1. A vertical shift of 800 pixels (equivalent to $0.2mm$ in the x-direction, Figure 2(b)).
2. A horizontal shift of 800 pixels (equivalent to $0.2mm$ in the y-direction, , Figure 2(c)).
3. A combined shift of 800 pixels in both the vertical and horizontal directions (equivalent to $0.2mm$ in both the x and y directions, Figure 2(d)).

[1] https://github.com/lukemelas/EfficientNet-PyTorch
[2] https://github.com/jacobgil/pytorch-grad-cam

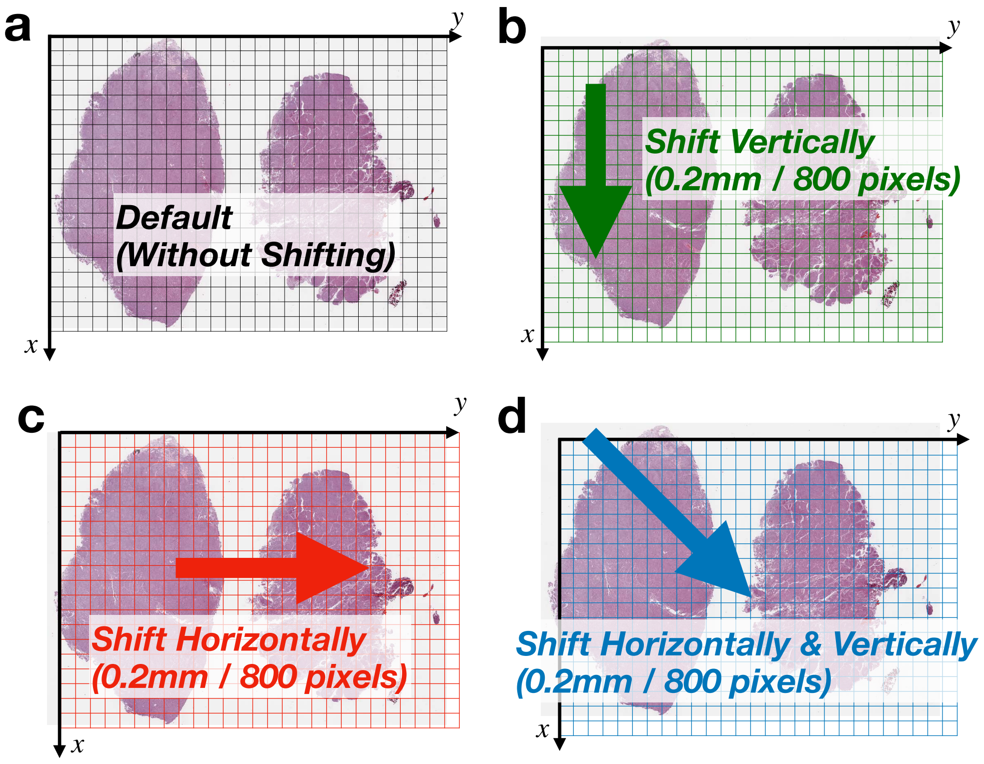

Fig. 2: Illustration of the shifting of coordinate origins

Under each shifting condition, the DFS algorithm showed a maximum increase of 2/WSI and a decrease of 1/WSI in MC. Interestingly, the linear and rectangular arrangements also displayed similar variation MC counts in these scenarios. On average, within each shifting condition, the DFS algorithm counted approximately 4.5 and 3.2 more mitoses than the linear and rectangular arrangements, respectively (refer to `06_MC_shift_supp.xlsx` for more details). This suggests that the differential of the DFS compared to linear or rectangular methods remains consistent with the findings reported in our manuscript.

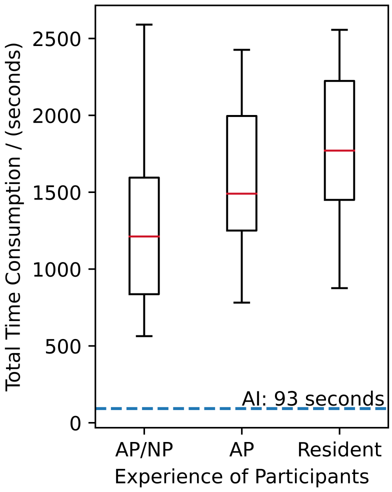

Fig. 3: Participants' time usage in annotating the 48 HPF images in the user study, grouped by the experience level.

5 Participants' Time Usage in Annotating the 48 Images the User Study

Figure 3 shows the participants' time usage in annotating the 48 HPF images in the user study, grouped by the experience level (i.e., AP/NP, AP, and residents). The median time usage for all participants was 23 minutes and 8 seconds (average = 24 minutes 32 seconds). The median time usage for AP/NP participants was 20 minutes and 12 seconds. For AP and resident participants, the median time usage were 24 minutes and 50 seconds, and 29 minutes and 29 seconds, respectively. However, the Kruskal–Wallis test did not reveal a significant difference ($p = 0.069$, $\eta_H^2 = 0.063$) in time usage by participants' experience levels.

6 Mitosis Count for Two Canine Datasets

Figure 3 shows the MC quantification of the DFS algorithm, linear (1×10 and 10×1 HPFs), and rectangular (2×5 and 5×2 HPFs) in canine mammary carcinoma [6] and canine cutaneous mast cell tumors [7], with one HPF having the size of $0.4mm \times$

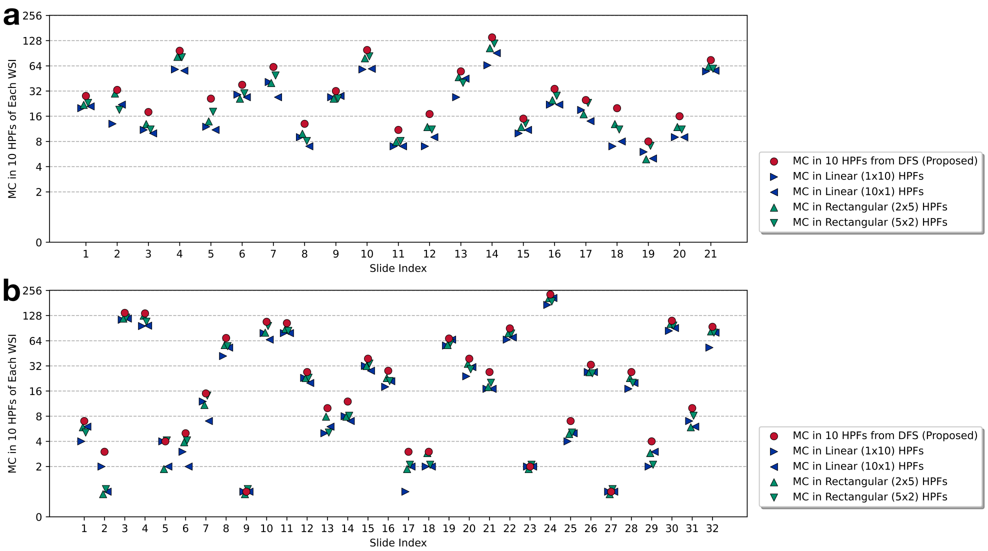

Fig. 4: Mitosis counts in two fully-slide-annotated mitosis datasets: (a) canine mammary carcinoma [6]; (b) canine cutaneous mast cell tumor [7].

$0.4mm = 0.16mm^2$. The MCs counted in 10 HPFs calculated by the DFS algorithm are higher than linear or rectangular arrangements in general.

7 Other Supplementary Materials

Other supplementary materials are submitted alongside with this document, including:

- `01_supp_info_sheet.xlsx`: A de-identified information sheet of mitosis count summary for each WSI.
- `02_User-study-key.pdf`: Mitosis annotations of the 48 images in the user study.
- `03_Report-AI-typical.pdf`: Performance report of a typical AI prediction on the 48 HPF images.
- `04_Report-participant-2b0577d1-e8ae-4d0a-bc75-7fcf3cfa39d7.pdf` and `05_Report-participant-d275b5c4-be08-4907-a032-62c6adfb8a34.pdf` Performance reports of the top-2 participants that achieved the best performance in the user study.

- `06_MC_shift_supp.xlsx`: Mitosis count in 10 HPFs with the DFS, linear, and rectangular arrangements, with regard to the shift in the coordinate origins.

References

[1] Tan M, Le Q. Efficientnet: Rethinking model scaling for convolutional neural networks. In: International conference on machine learning. PMLR; 2019. p. 6105–6114.

[2] Xue C, Dou Q, Shi X, Chen H, Heng PA. Robust learning at noisy labeled medical images: Applied to skin lesion classification. In: 2019 IEEE 16th International Symposium on Biomedical Imaging (ISBI 2019). IEEE; 2019. p. 1280–1283.

[3] Cao W, Mirjalili V, Raschka S. Rank consistent ordinal regression for neural networks with application to age estimation. Pattern Recognition Letters. 2020;140:325–331.

[4] Gu H, Haeri M, Ni S, Williams CK, Zarrin-Khameh N, Magaki S, et al. Detecting Mitoses with a Convolutional Neural Network for MIDOG 2022 Challenge. In: Sheng B, Aubreville M, editors. Mitosis Domain Generalization and Diabetic Retinopathy Analysis. Cham: Springer Nature Switzerland; 2023. p. 211–216.

[5] Chattopadhay A, Sarkar A, Howlader P, Balasubramanian VN. Grad-CAM++: Generalized Gradient-Based Visual Explanations for Deep Convolutional Networks. In: 2018 IEEE Winter Conference on Applications of Computer Vision (WACV); 2018. p. 839–847.

[6] Aubreville M, Bertram CA, Donovan TA, Marzahl C, Maier A, Klopfleisch R. A completely annotated whole slide image dataset of canine breast cancer to aid human breast cancer research. Scientific Data. 2020 Nov;7(1):417. https://doi.

org/10.1038/s41597-020-00756-z.

[7] Bertram CA, Aubreville M, Marzahl C, Maier A, Klopfleisch R. A large-scale dataset for mitotic figure assessment on whole slide images of canine cutaneous mast cell tumor. Scientific Data. 2019 Nov;6(1):274. https://doi.org/10.1038/s41597-019-0290-4.
