# Supplementary material for: Enhancing mitosis quantification and detection in meningiomas with computational digital pathology
Source: Acta Neuropathol Commun. 2024 Jan 11;12:7. doi: 10.1186/s40478-023-01707-6 (PMC10782692; doi:10.1186/s40478-023-01707-6)

Mitosis Annotations for the 48 High-Power Field Images in the User Study

Red arrow: mitosis

H&E (1HPF, 0.16mm^2)

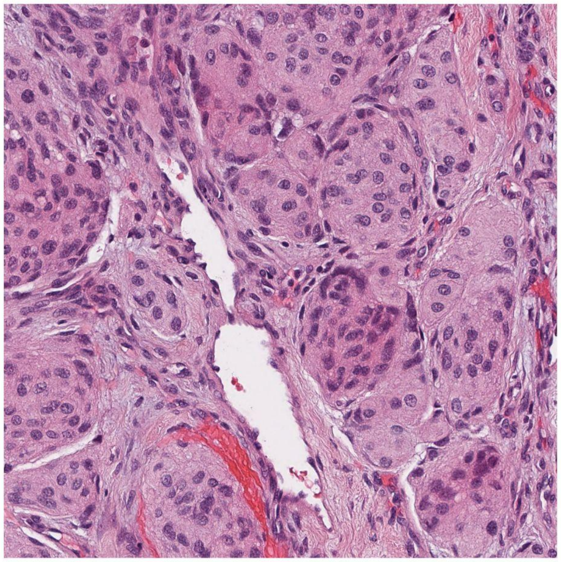

Phosphorylated Histone H3 (0.16mm^2)

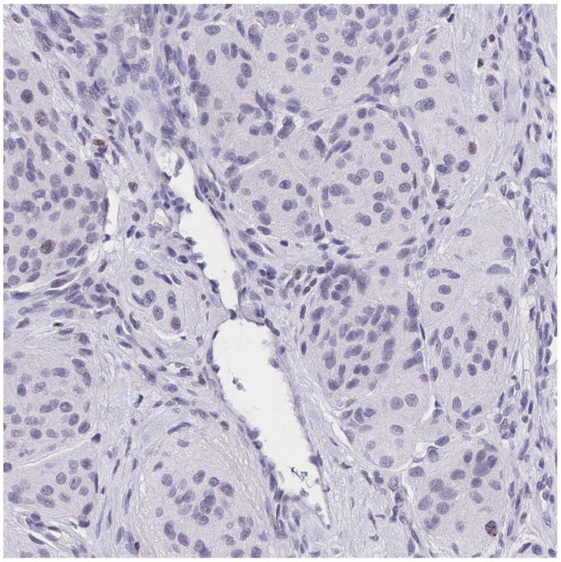

Red arrow: mitosis

H&E (1HPF, 0.16mm^2)

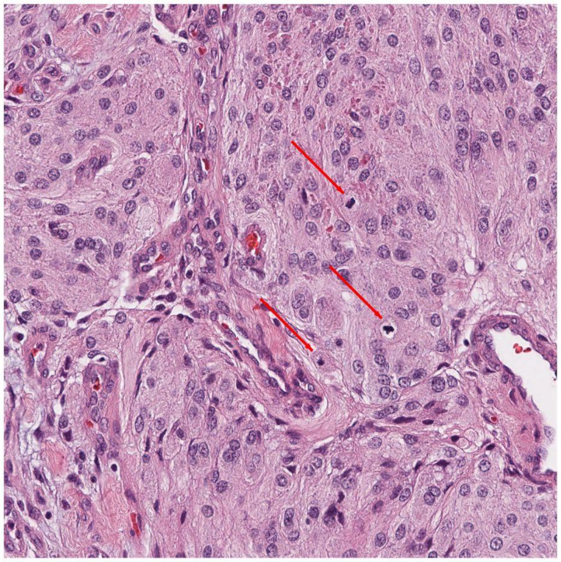

Phosphorylated Histone H3 (0.16mm^2)

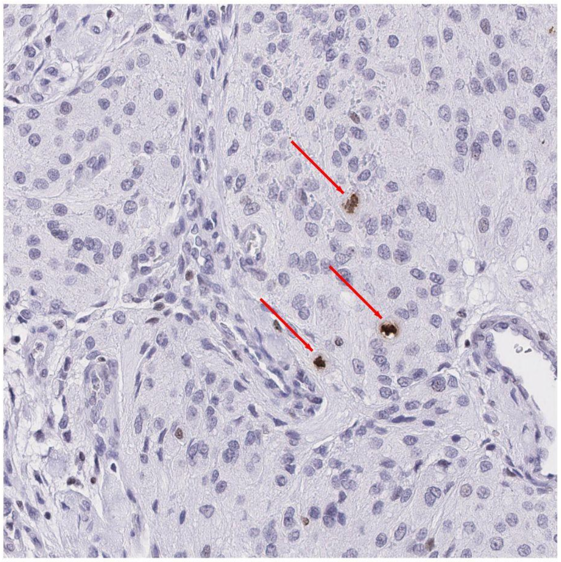

Red arrow: mitosis

H&E (1HPF, 0.16mm^2)

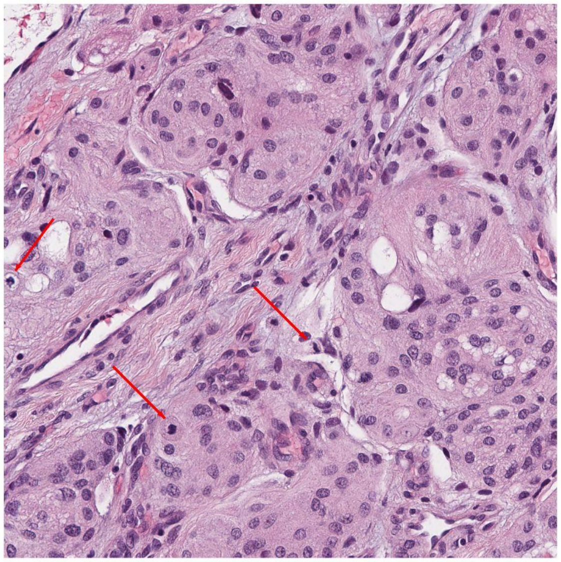

Phosphorylated Histone H3 (0.16mm^2)

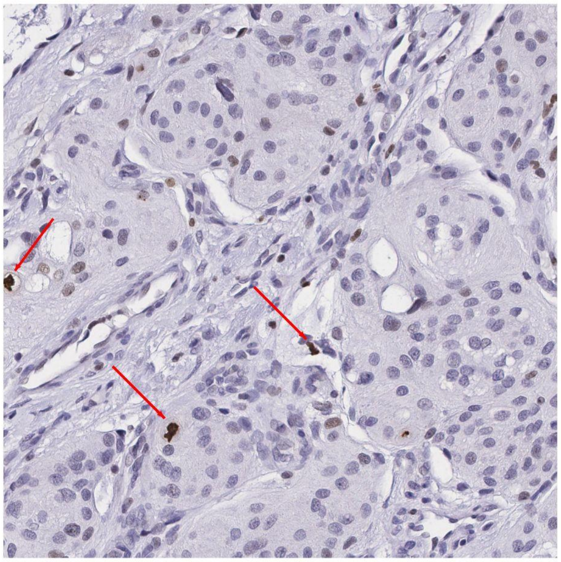

Red arrow: mitosis

H&E (1HPF, 0.16mm^2)

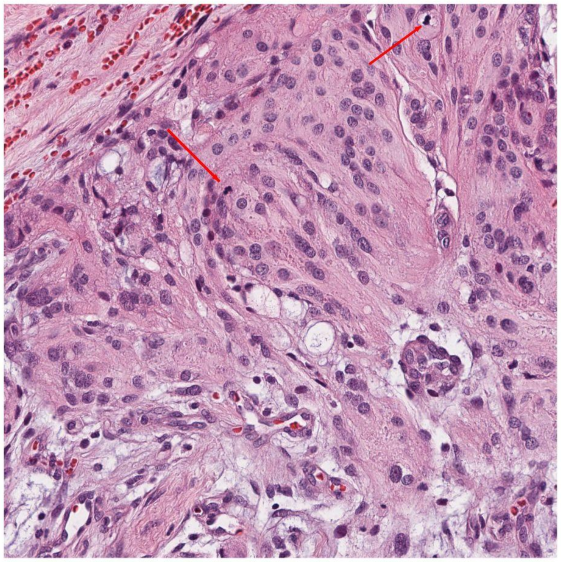

Phosphorylated Histone H3 (0.16mm^2)

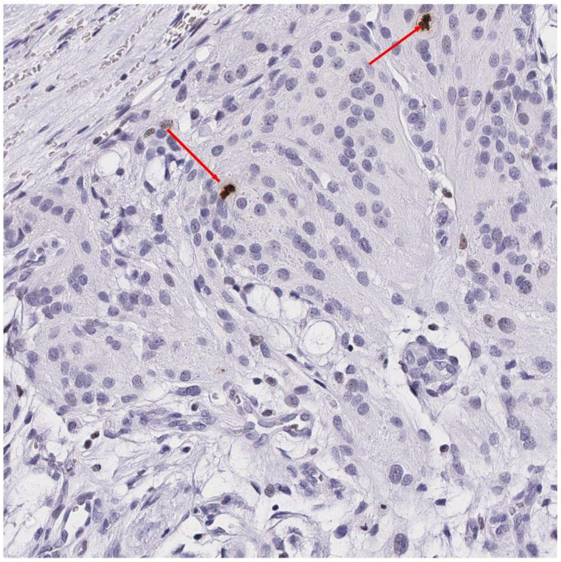

Red arrow: mitosis

H&E (1HPF, 0.16mm^2)

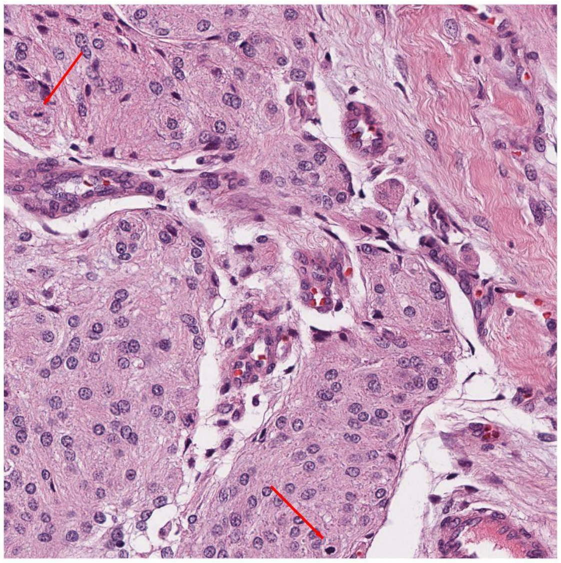

Phosphorylated Histone H3 (0.16mm^2)

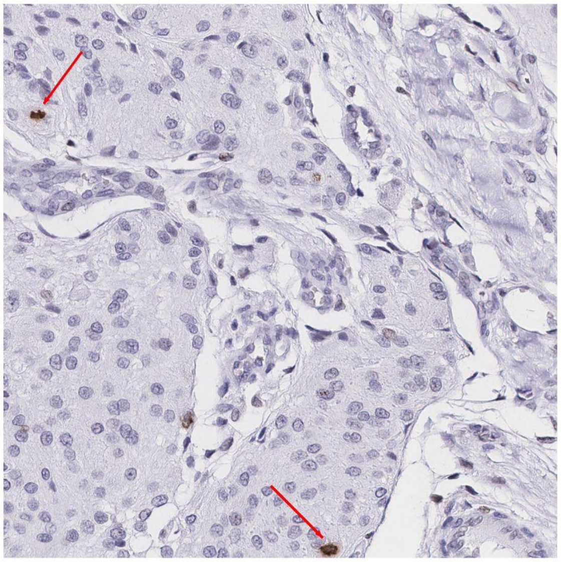

Red arrow: mitosis

H&E (1HPF, 0.16mm^2)

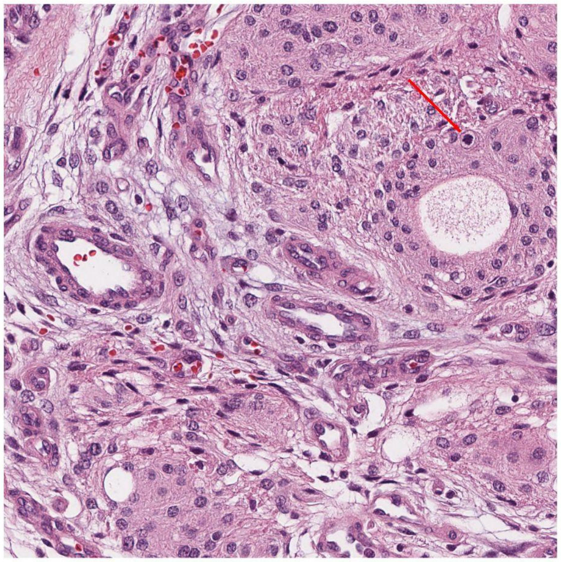

Phosphorylated Histone H3 (0.16mm^2)

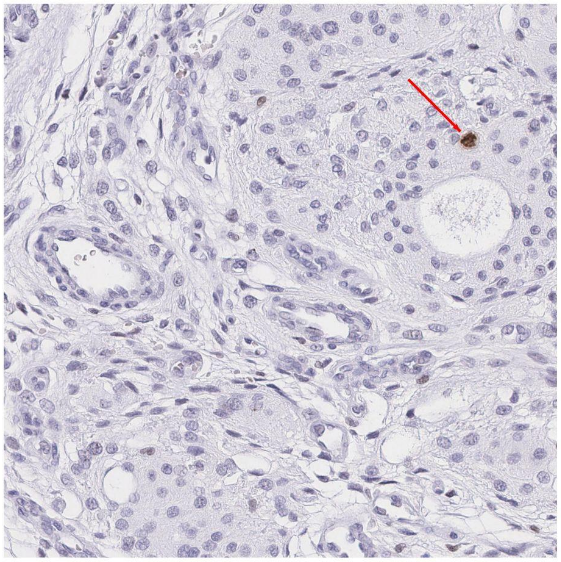

Red arrow: mitosis

H&E (1HPF, 0.16mm^2)

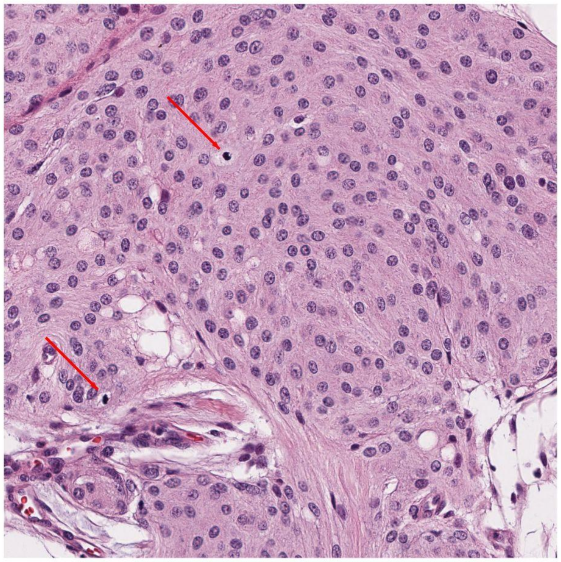

Phosphorylated Histone H3 (0.16mm^2)

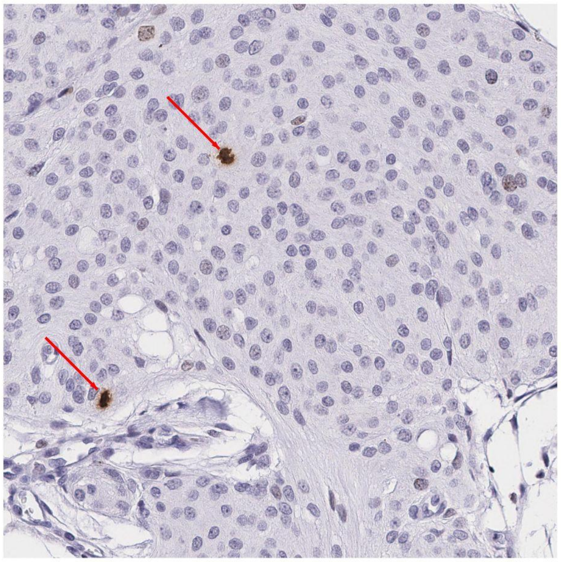

Red arrow: mitosis

H&E (1HPF, 0.16mm^2)

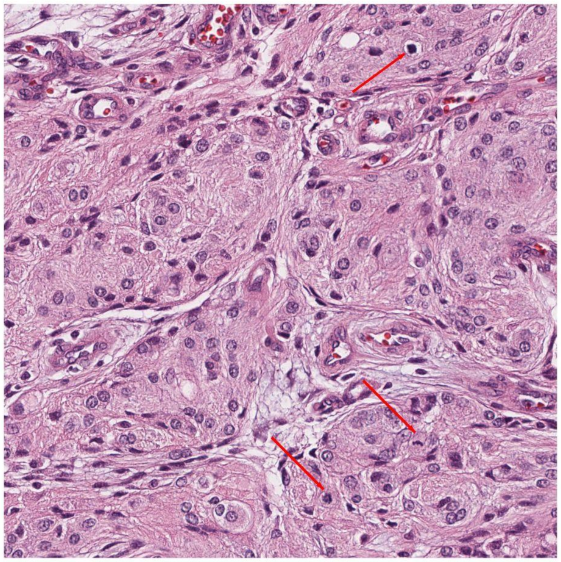

Phosphorylated Histone H3 (0.16mm^2)

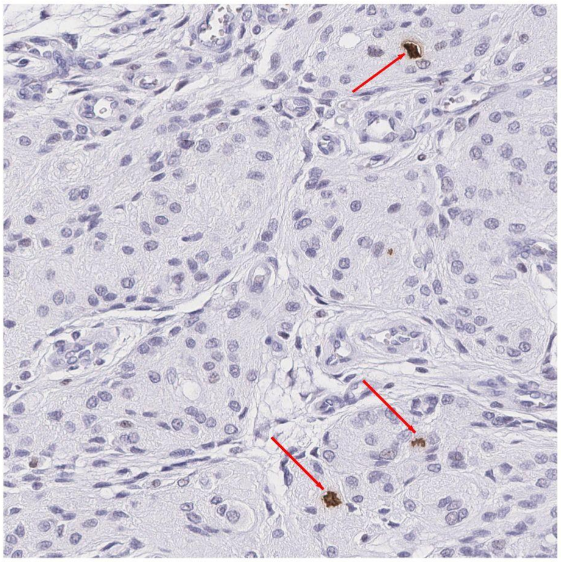

Red arrow: mitosis

H&E (1HPF, 0.16mm^2)

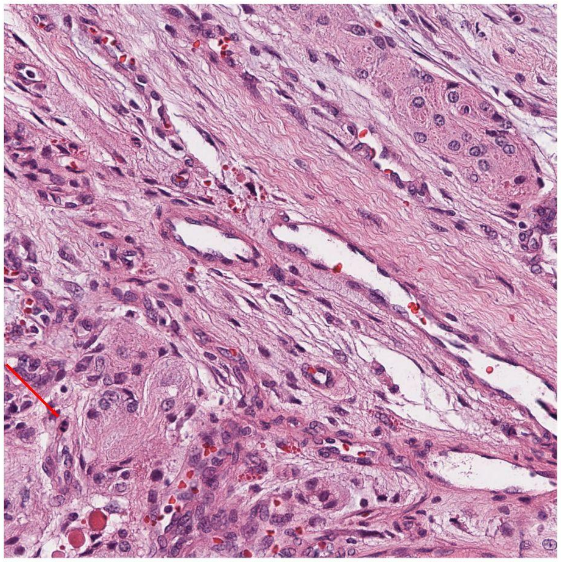

Phosphorylated Histone H3 (0.16mm^2)

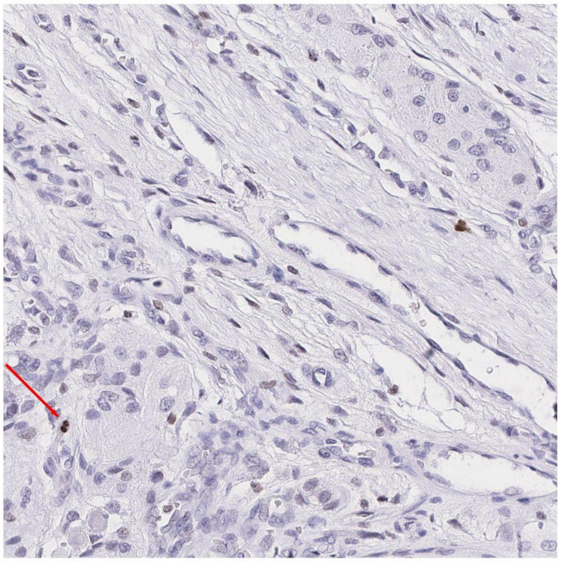

Red arrow: mitosis

H&E (1HPF, 0.16mm^2)

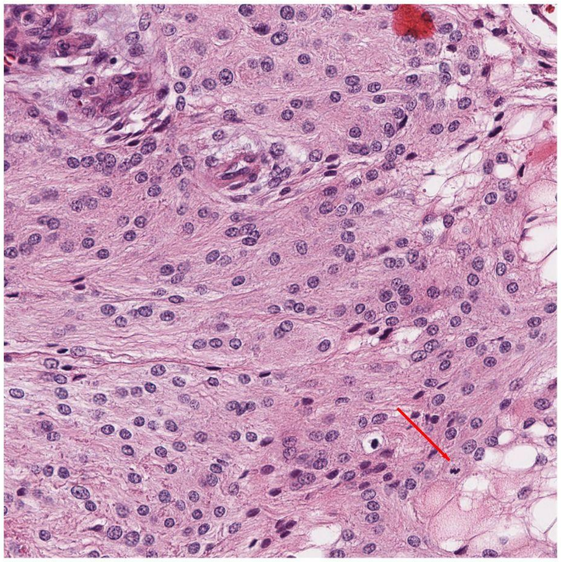

Phosphorylated Histone H3 (0.16mm^2)

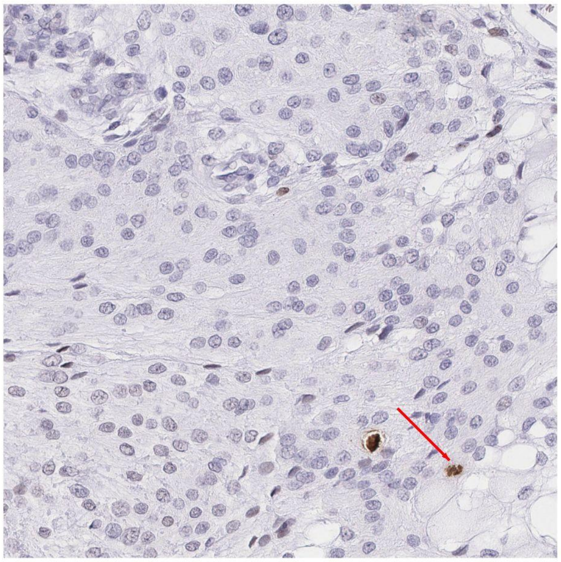

Red arrow: mitosis

H&E (1HPF, 0.16mm^2)

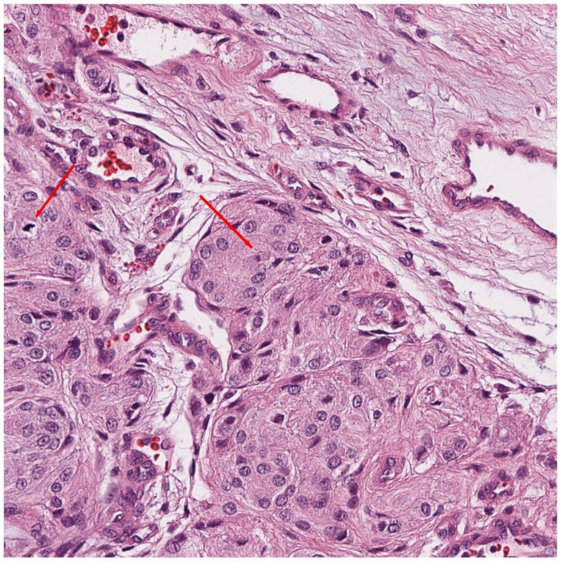

Phosphorylated Histone H3 (0.16mm^2)

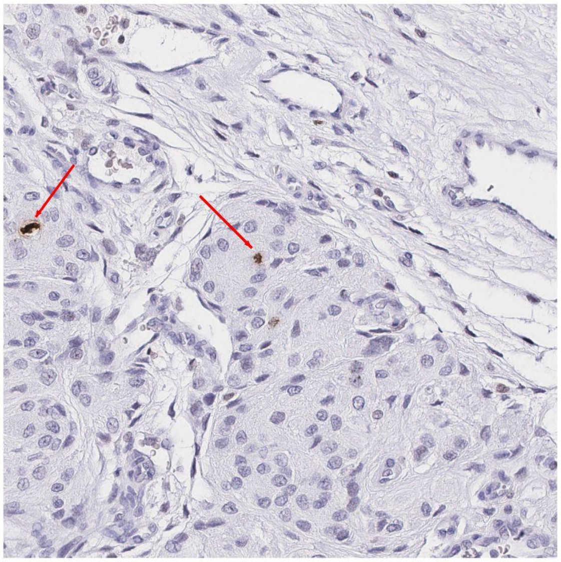

Red arrow: mitosis

H&E (1HPF, 0.16mm^2)

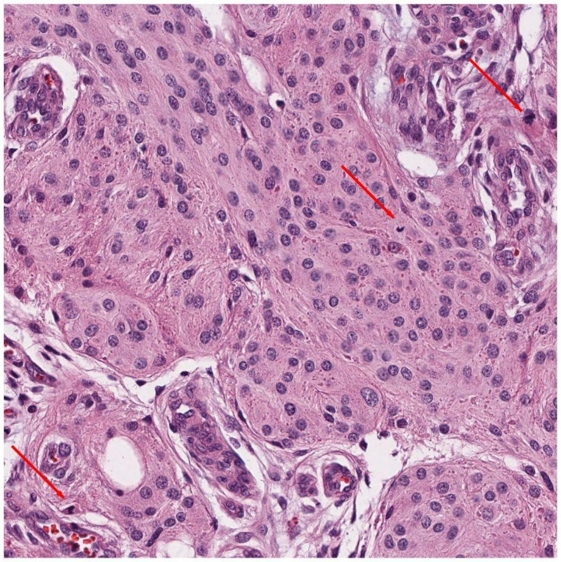

Phosphorylated Histone H3 (0.16mm^2)

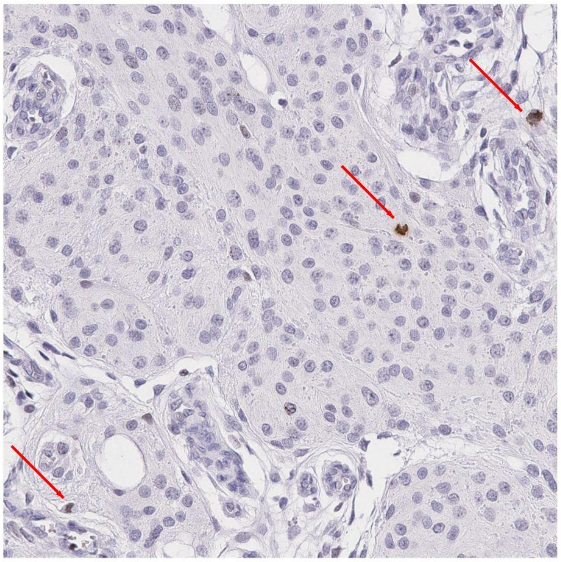

Red arrow: mitosis

H&E (1HPF, 0.16mm^2)

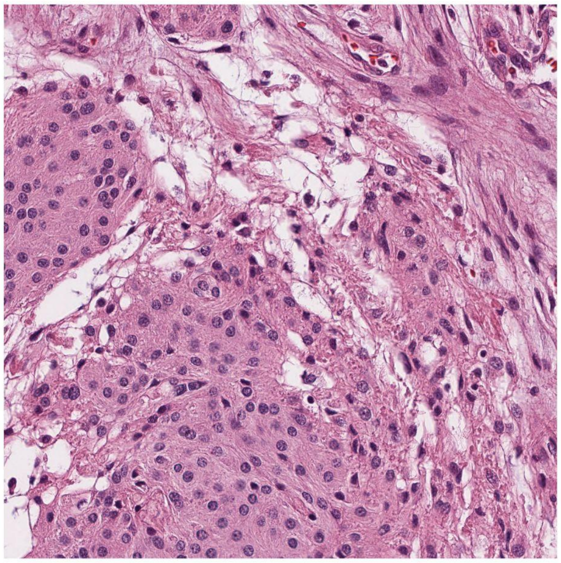

Phosphorylated Histone H3 (0.16mm^2)

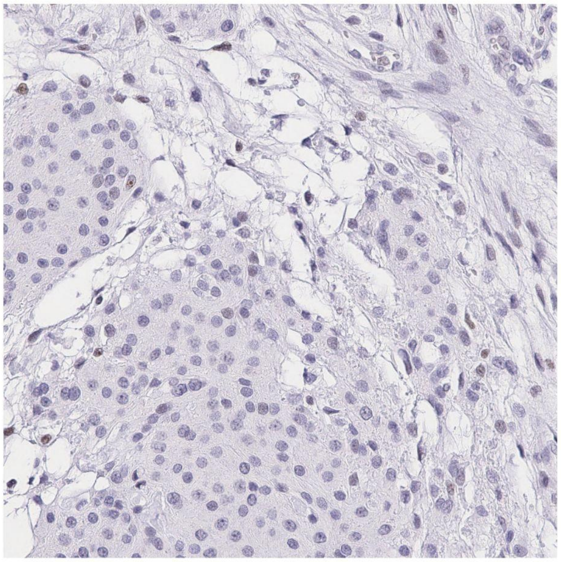

Red arrow: mitosis

H&E (1HPF, 0.16mm^2)

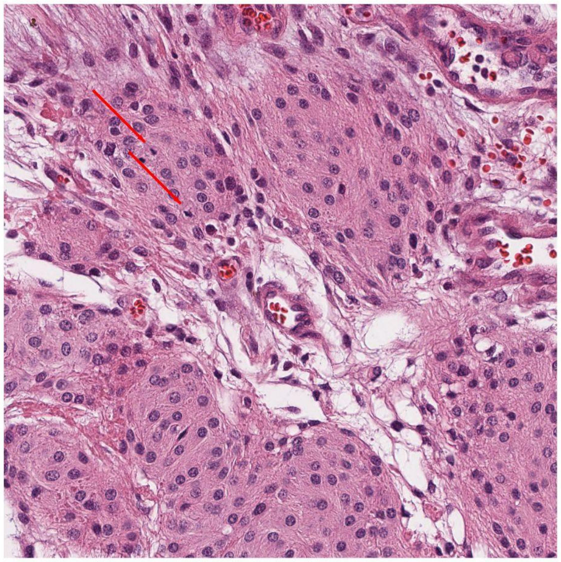

Phosphorylated Histone H3 (0.16mm^2)

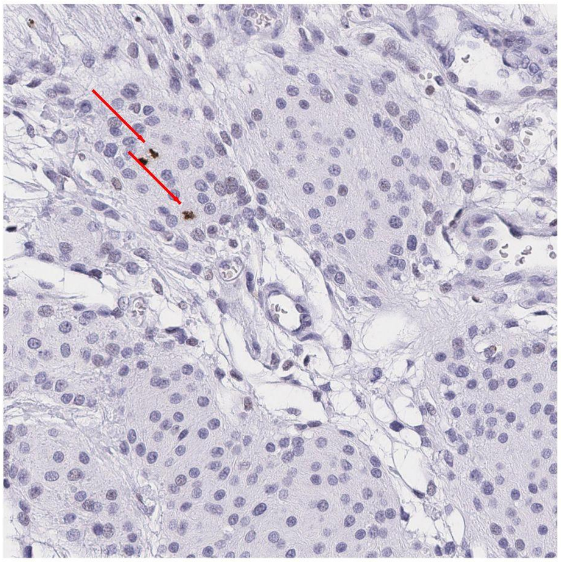

Red arrow: mitosis

H&E (1HPF, 0.16mm^2)

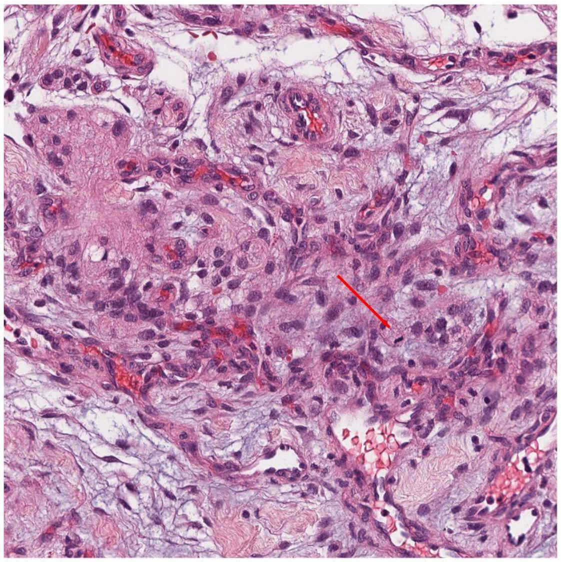

Phosphorylated Histone H3 (0.16mm^2)

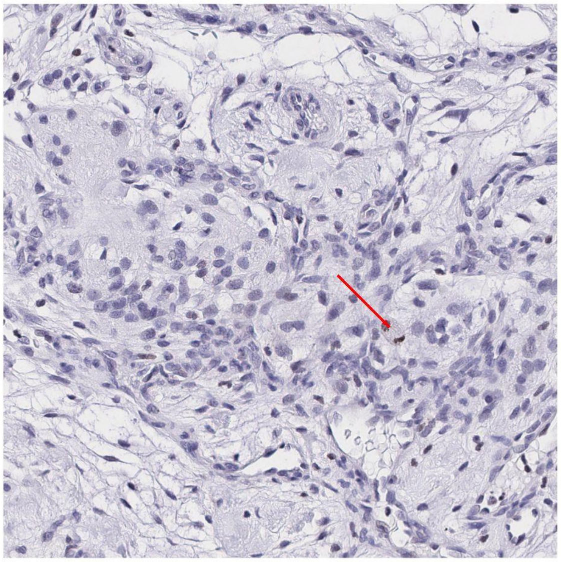

Red arrow: mitosis

H&E (1HPF, 0.16mm^2)

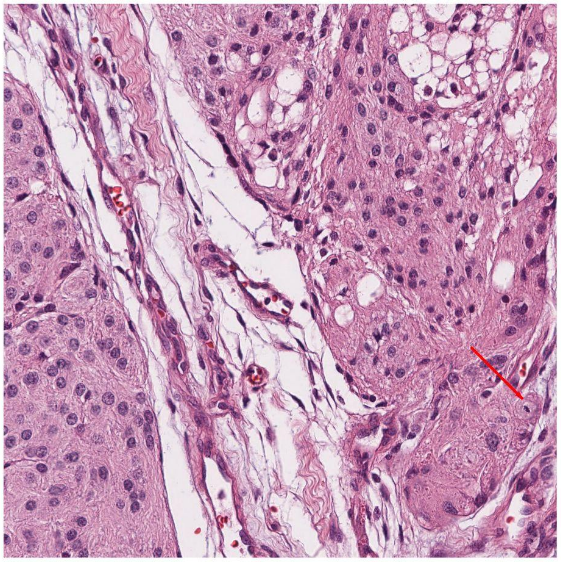

Phosphorylated Histone H3 (0.16mm^2)

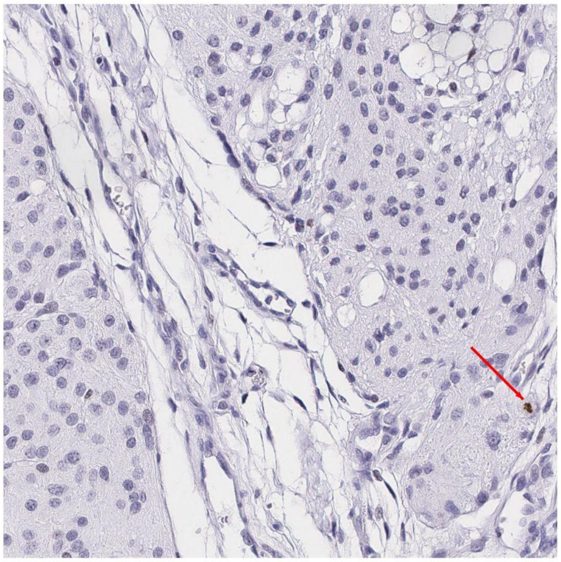

Red arrow: mitosis

H&E (1HPF, 0.16mm^2)

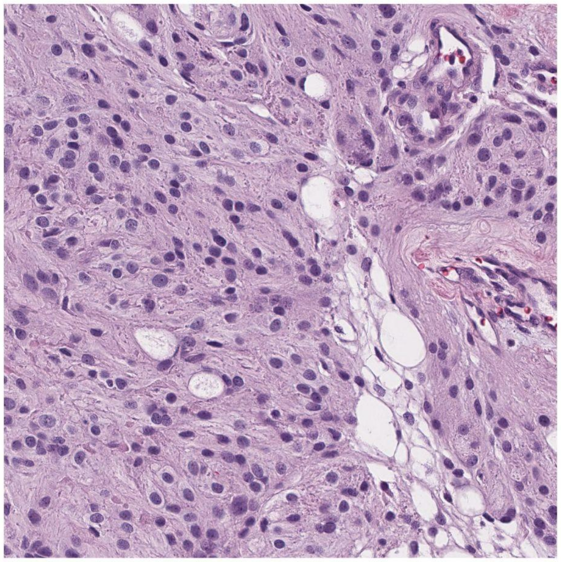

Phosphorylated Histone H3 (0.16mm^2)

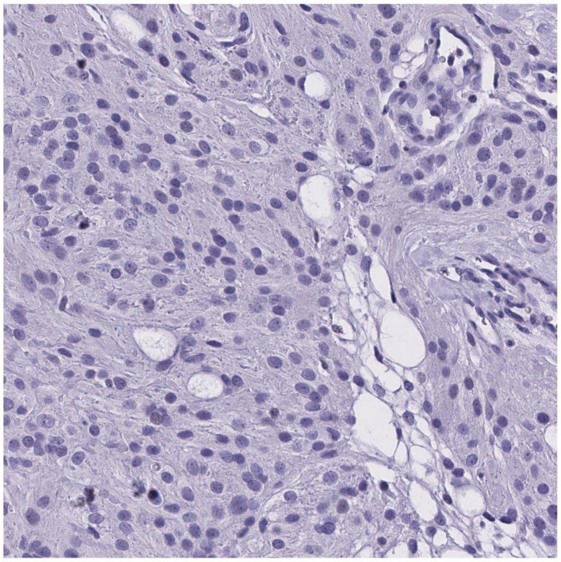

Red arrow: mitosis

H&E (1HPF, 0.16mm^2)

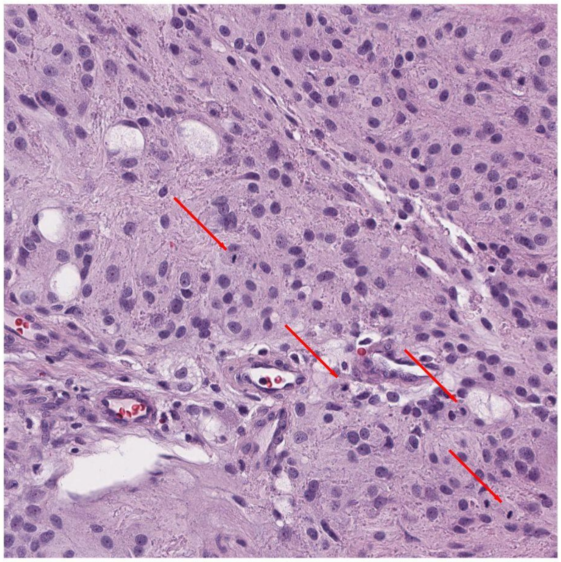

Phosphorylated Histone H3 (0.16mm^2)

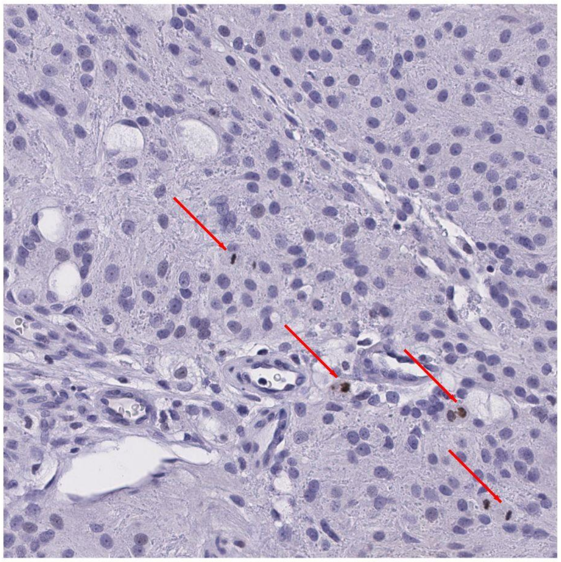

Red arrow: mitosis

H&E (1HPF, 0.16mm^2)

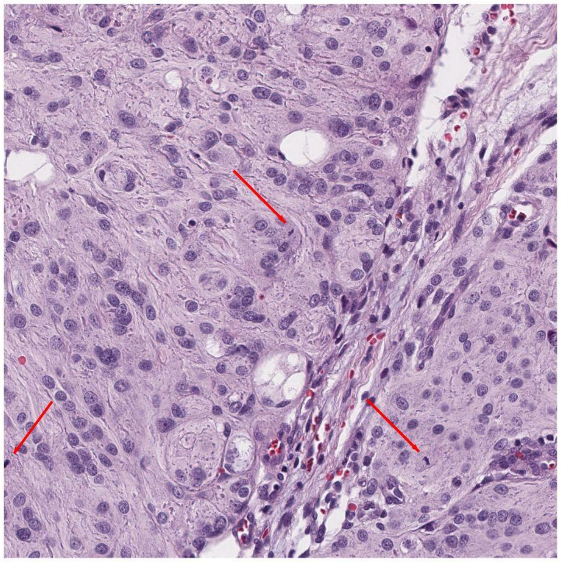

Phosphorylated Histone H3 (0.16mm^2)

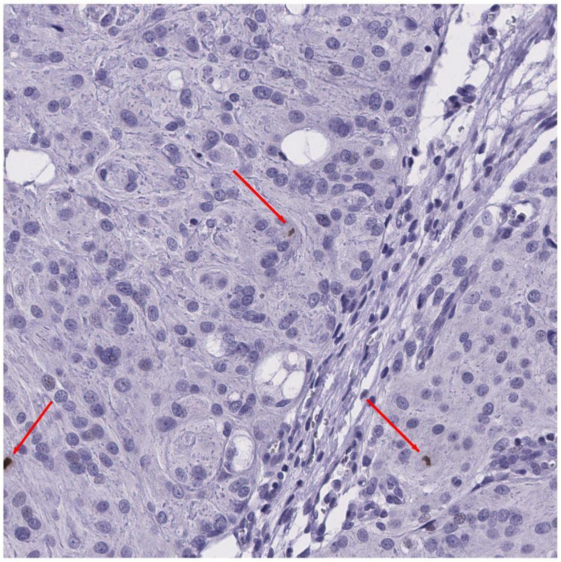

Red arrow: mitosis

H&E (1HPF, 0.16mm^2)

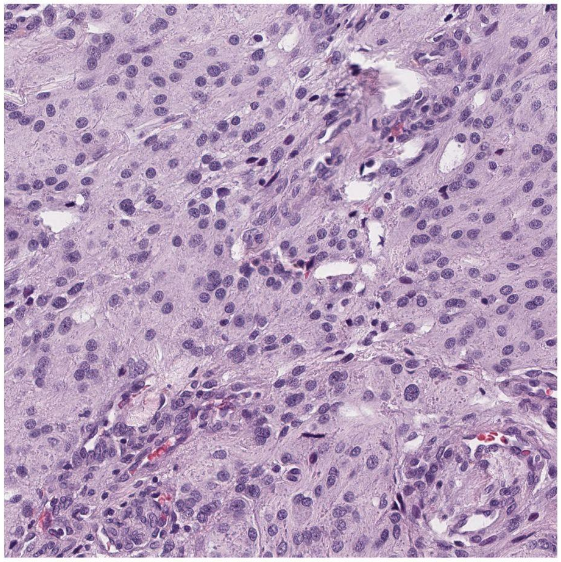

Phosphorylated Histone H3 (0.16mm^2)

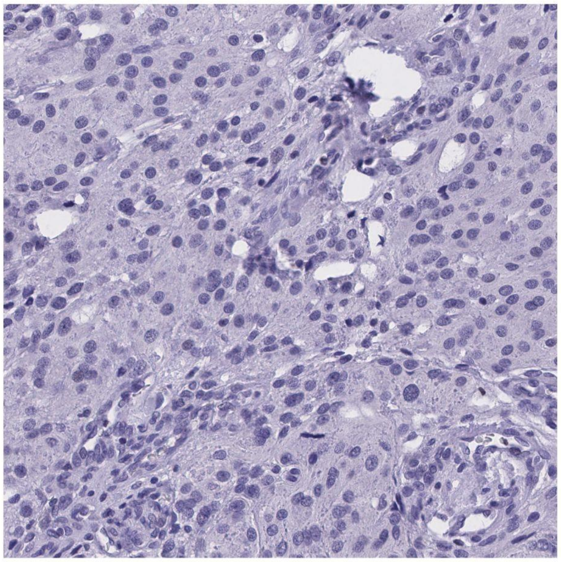

Red arrow: mitosis

H&E (1HPF, 0.16mm^2)

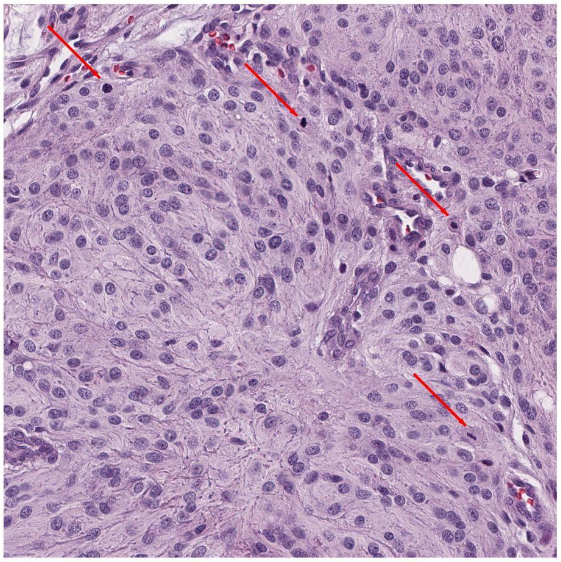

Phosphorylated Histone H3 (0.16mm^2)

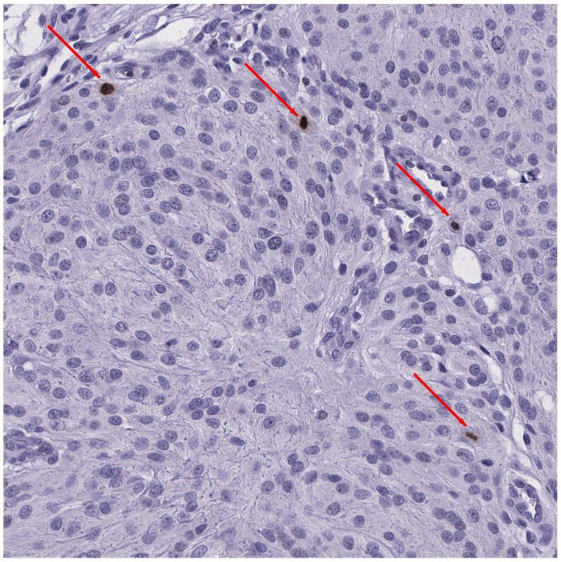

Red arrow: mitosis

H&E (1HPF, 0.16mm^2)

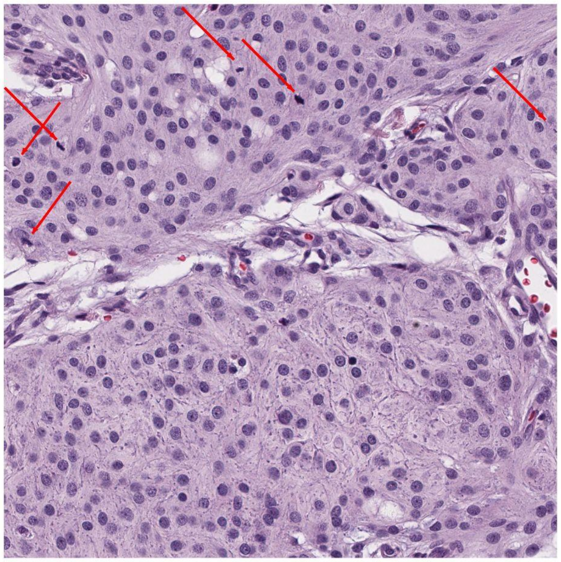

Phosphorylated Histone H3 (0.16mm^2)

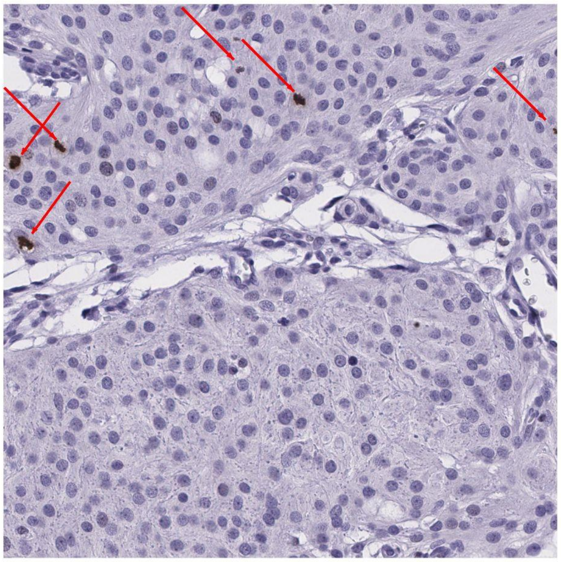

Red arrow: mitosis

H&E (1HPF, 0.16mm^2)

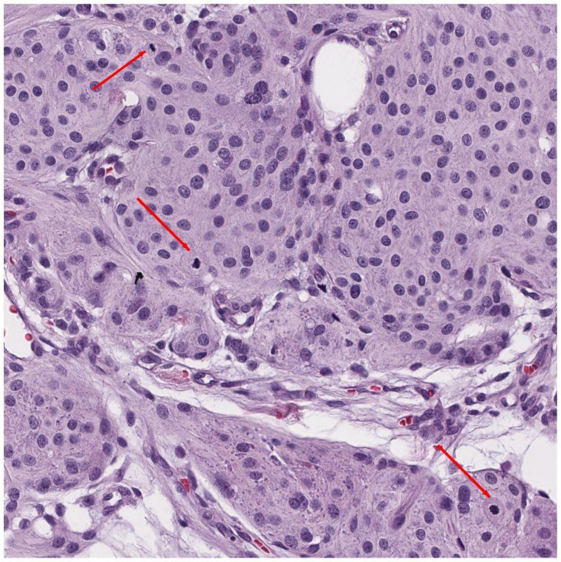

Phosphorylated Histone H3 (0.16mm^2)

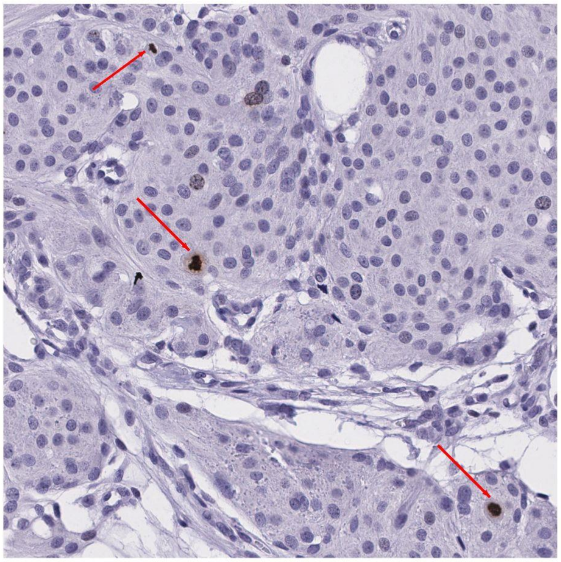

Red arrow: mitosis

H&E (1HPF, 0.16mm^2)

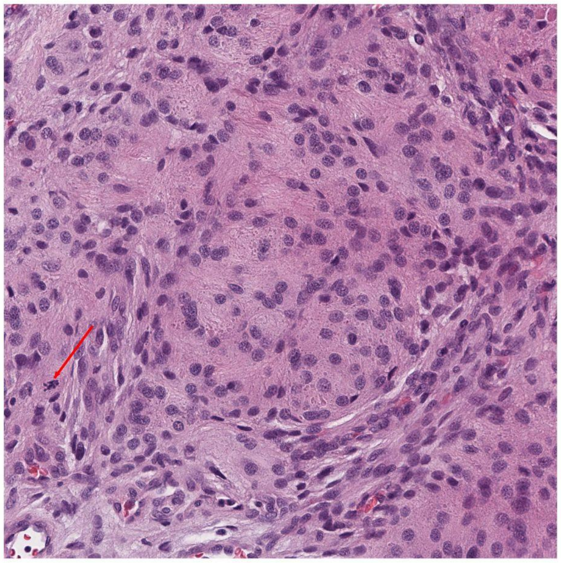

Phosphorylated Histone H3 (0.16mm^2)

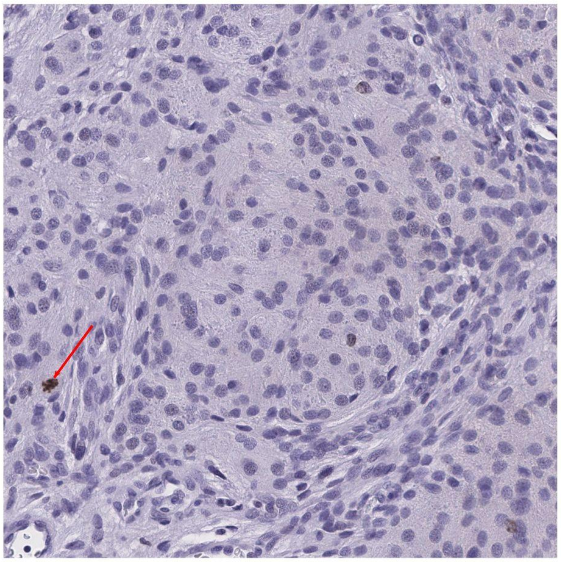

Red arrow: mitosis

H&E (1HPF, 0.16mm^2)

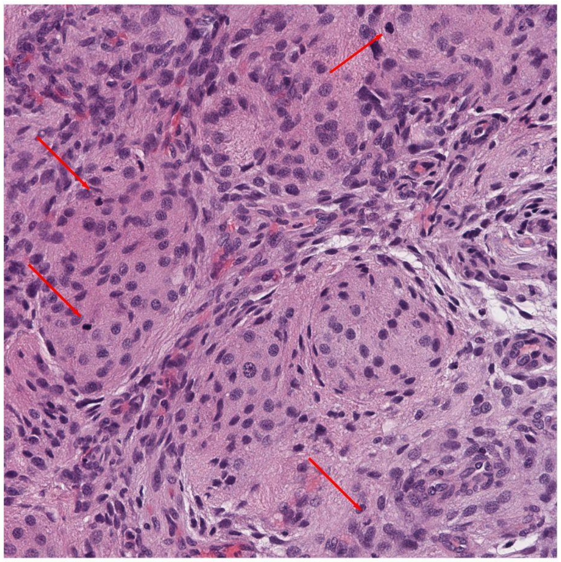

Phosphorylated Histone H3 (0.16mm^2)

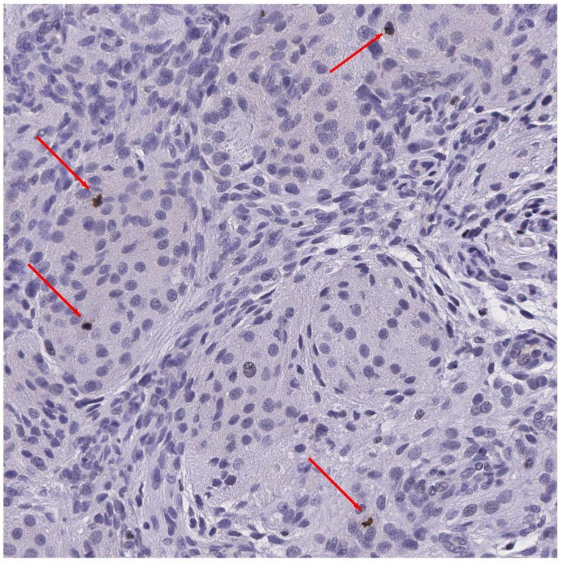

Red arrow: mitosis

H&E (1HPF, 0.16mm^2)

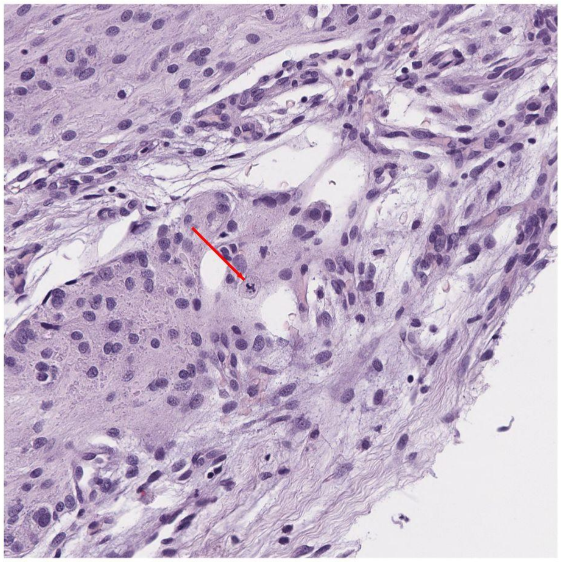

Phosphorylated Histone H3 (0.16mm^2)

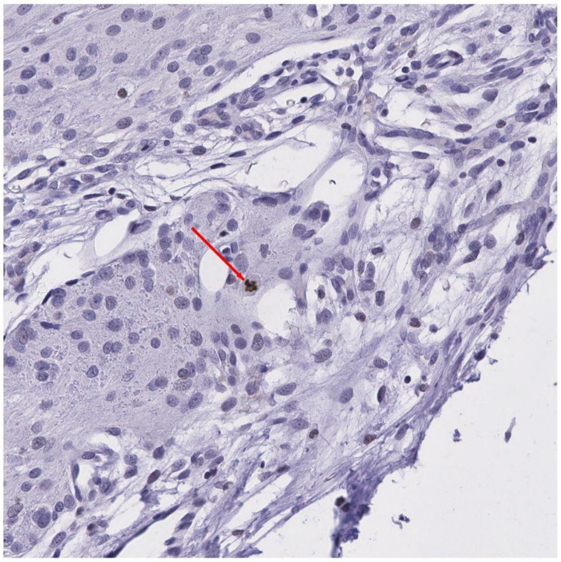

Red arrow: mitosis

H&E (1HPF, 0.16mm^2)

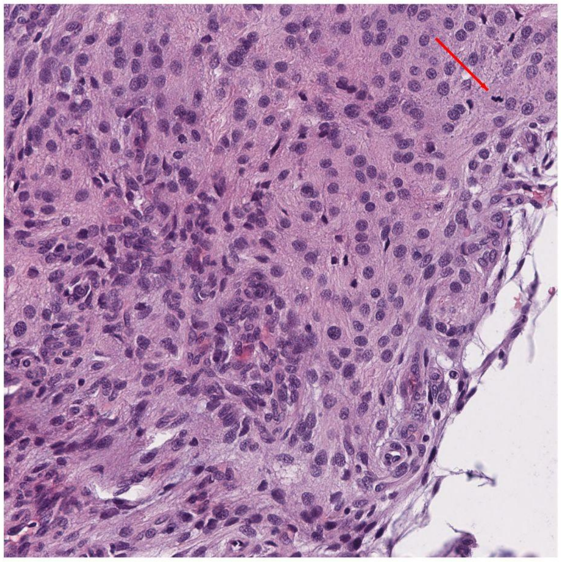

Phosphorylated Histone H3 (0.16mm^2)

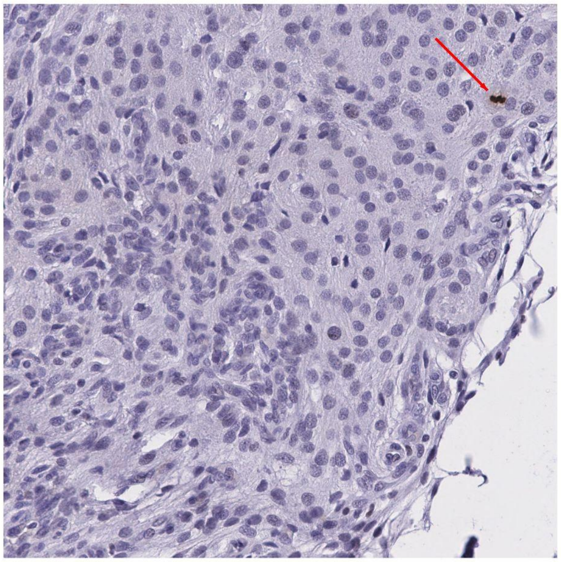

Red arrow: mitosis

H&E (1HPF, 0.16mm^2)

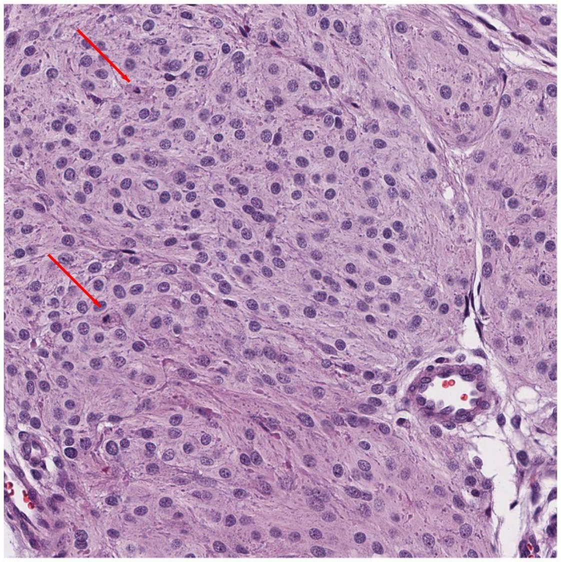

Phosphorylated Histone H3 (0.16mm^2)

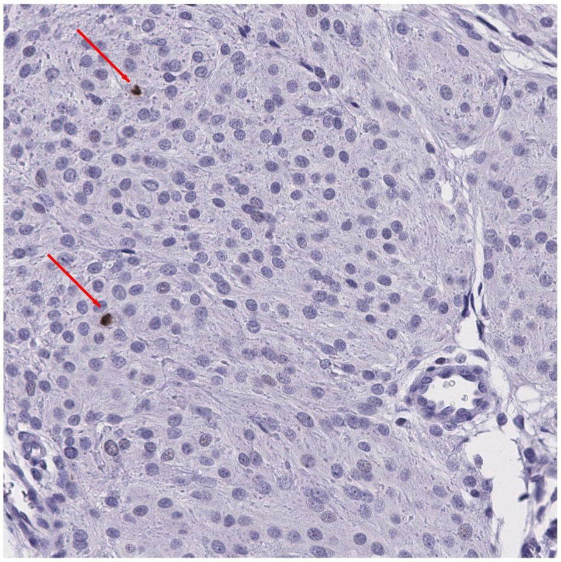

Red arrow: mitosis

H&E (1HPF, 0.16mm^2)

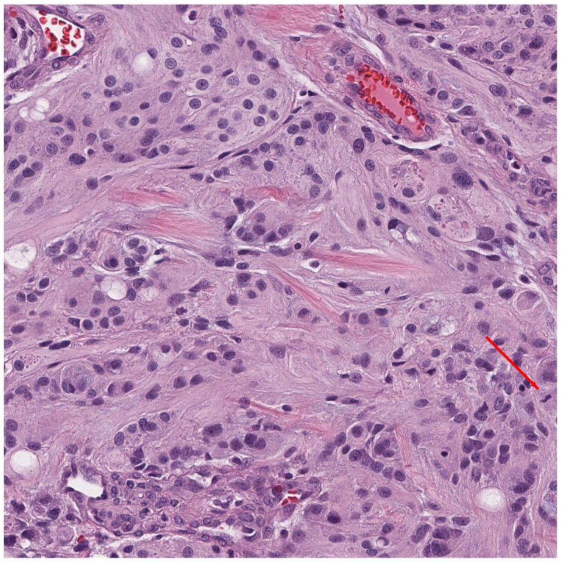

Phosphorylated Histone H3 (0.16mm^2)

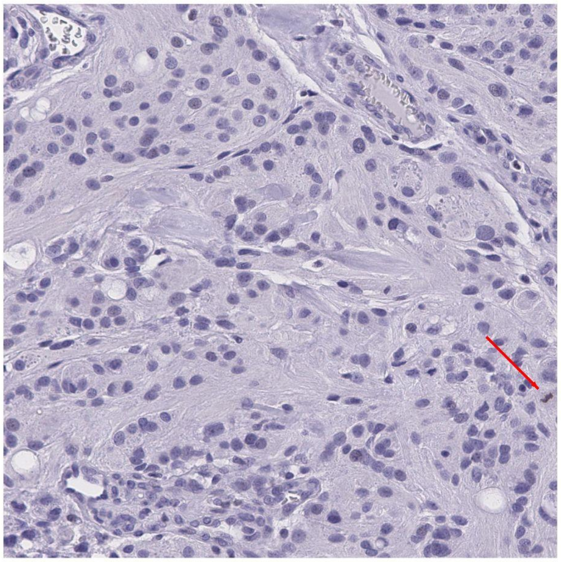

Red arrow: mitosis

H&E (1HPF, 0.16mm^2)

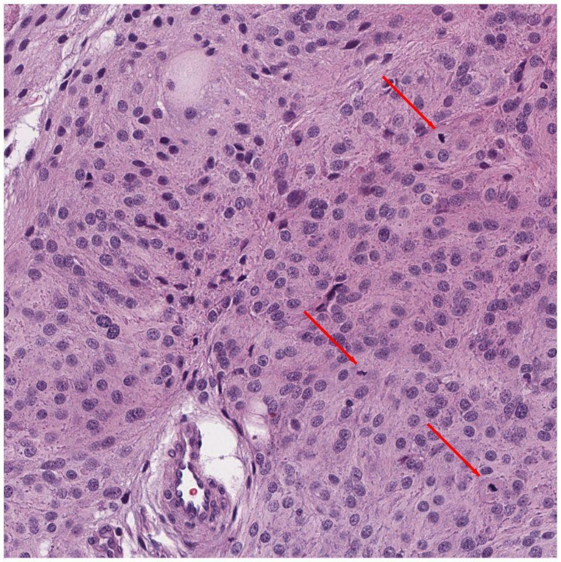

Phosphorylated Histone H3 (0.16mm^2)

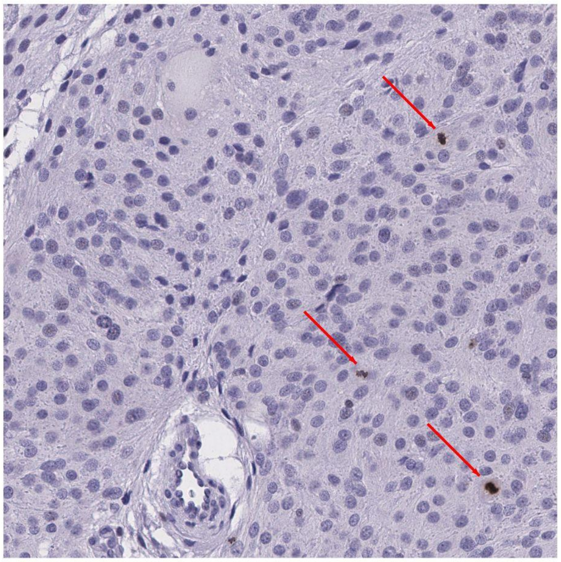

Red arrow: mitosis

H&E (1HPF, 0.16mm^2)

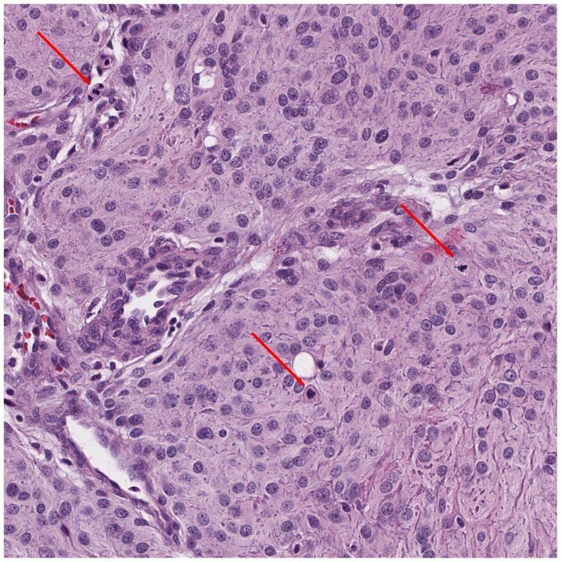

Phosphorylated Histone H3 (0.16mm^2)

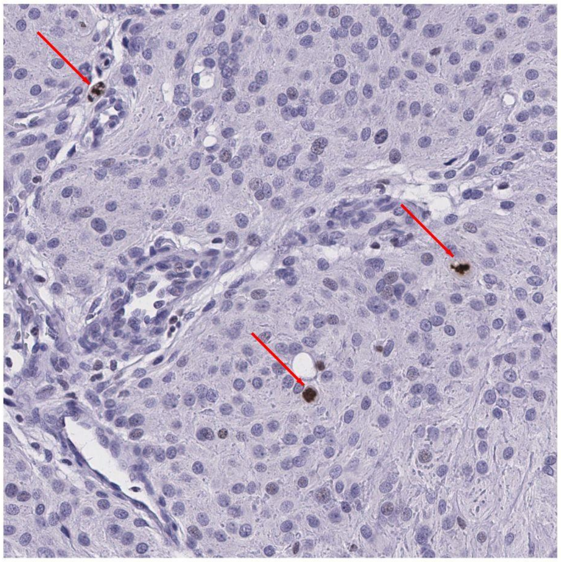

Red arrow: mitosis

H&E (1HPF, 0.16mm^2)

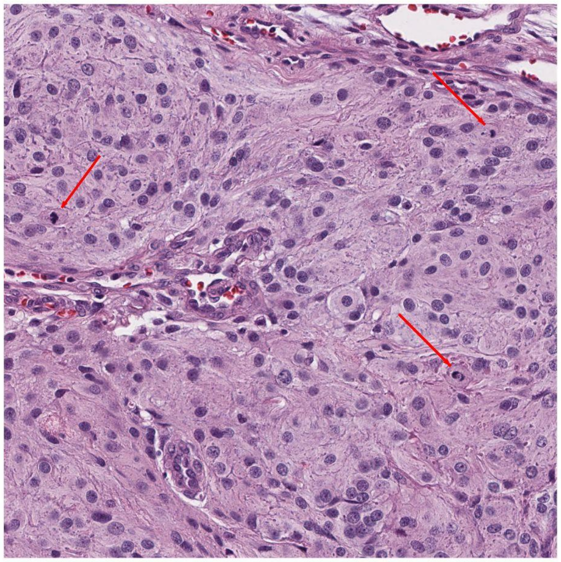

Phosphorylated Histone H3 (0.16mm^2)

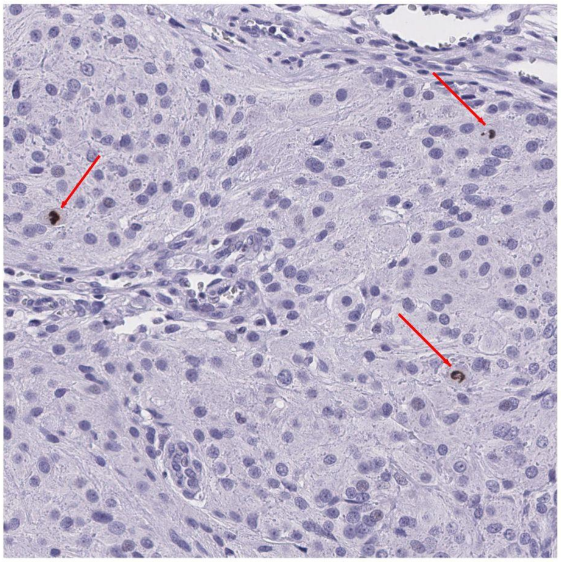

Red arrow: mitosis

H&E (1HPF, 0.16mm^2)

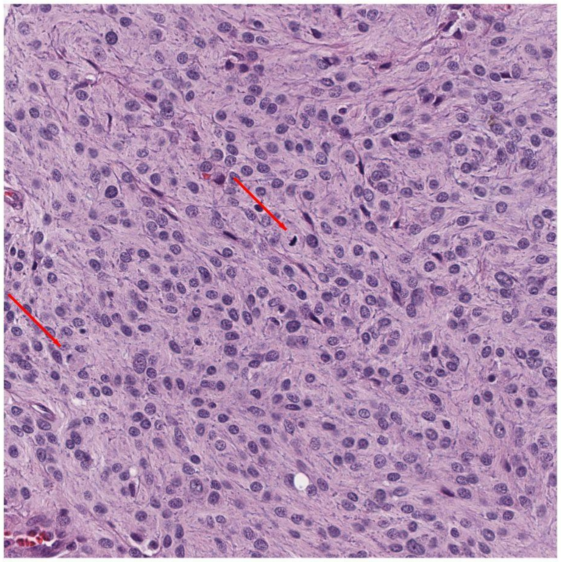

Phosphorylated Histone H3 (0.16mm^2)

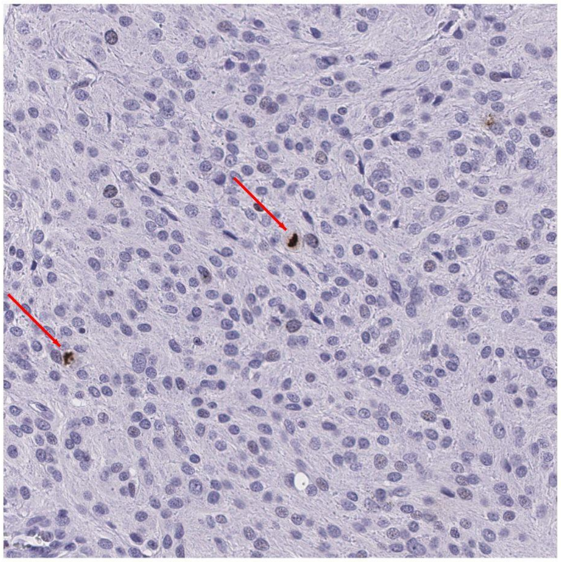

Red arrow: mitosis

H&E (1HPF, 0.16mm^2)

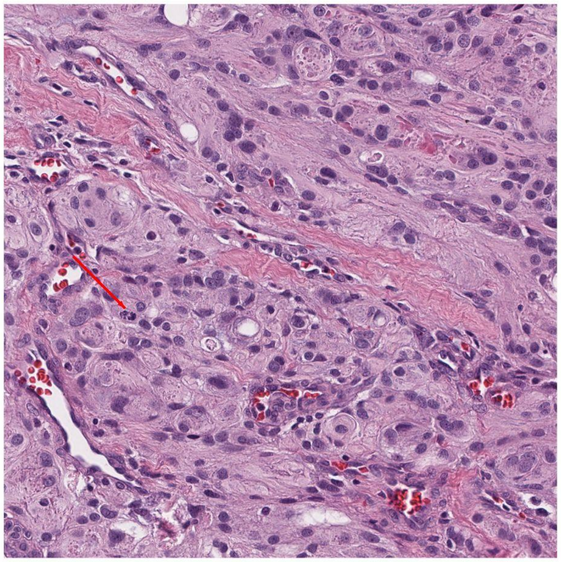

Phosphorylated Histone H3 (0.16mm^2)

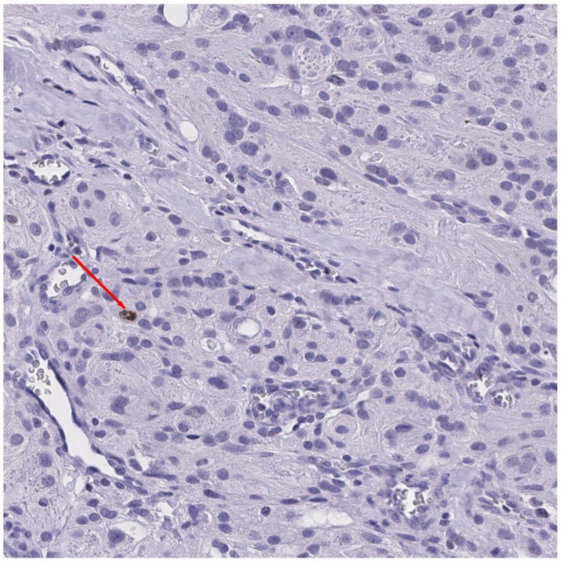

Red arrow: mitosis

H&E (1HPF, 0.16mm^2)

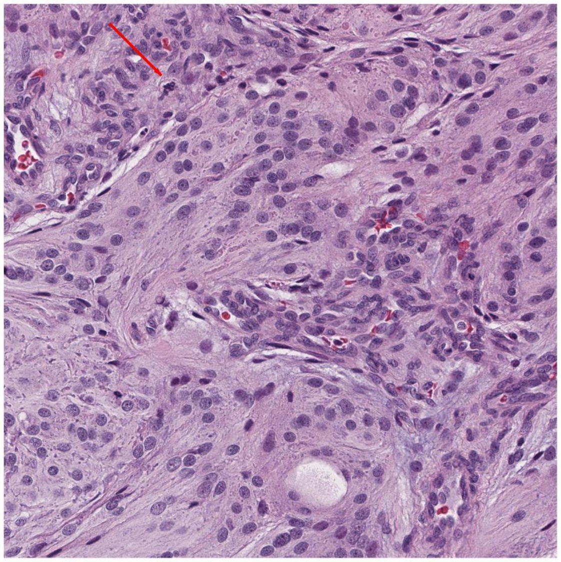

Phosphorylated Histone H3 (0.16mm^2)

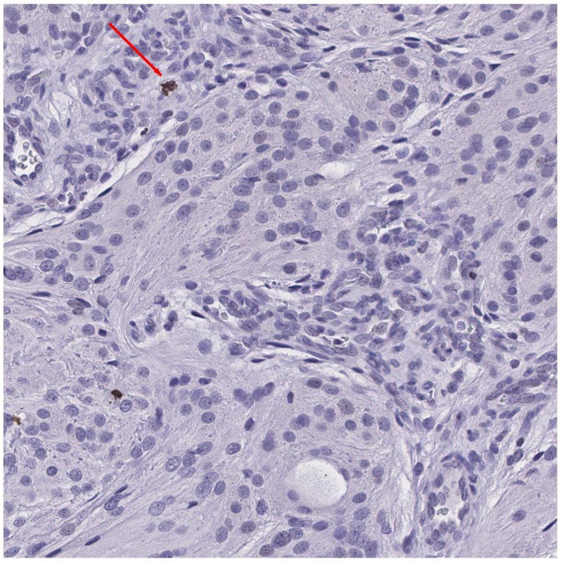

Red arrow: mitosis

H&E (1HPF, 0.16mm^2)

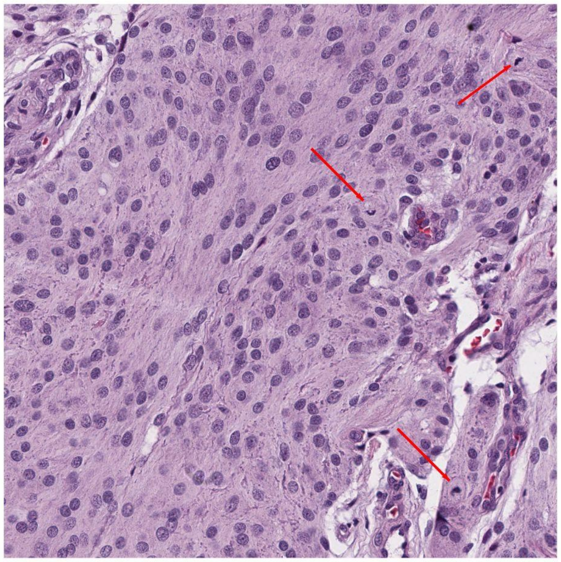

Phosphorylated Histone H3 (0.16mm^2)

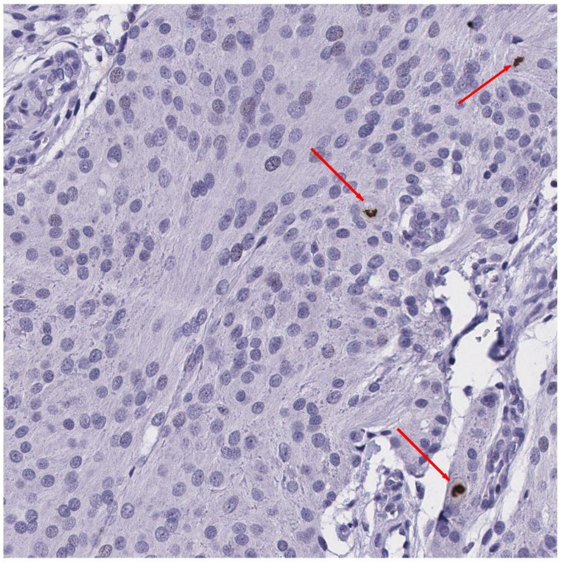

Red arrow: mitosis

H&E (1HPF, 0.16mm^2)

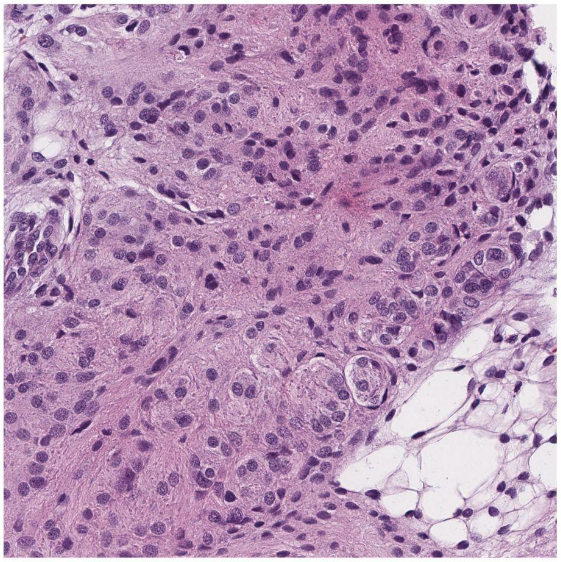

Phosphorylated Histone H3 (0.16mm^2)

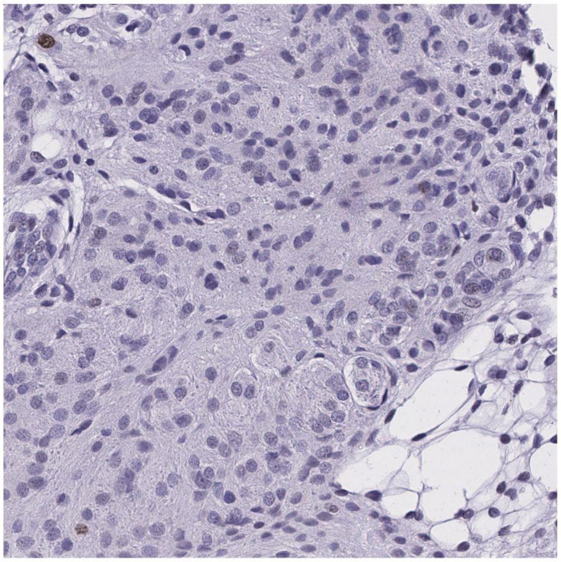

Red arrow: mitosis

H&E (1HPF, 0.16mm^2)

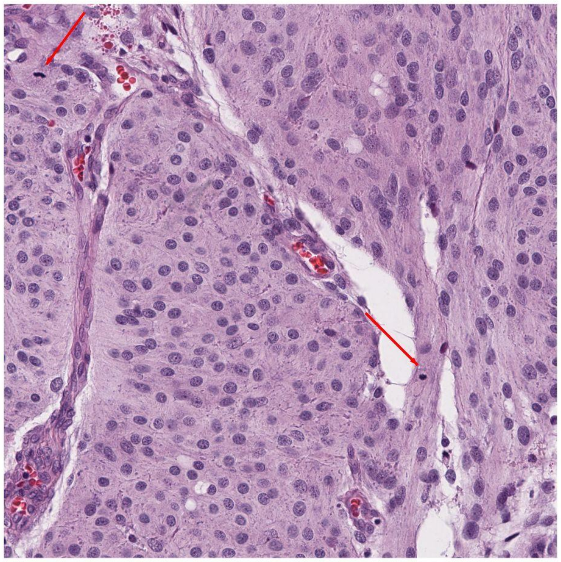

Phosphorylated Histone H3 (0.16mm^2)

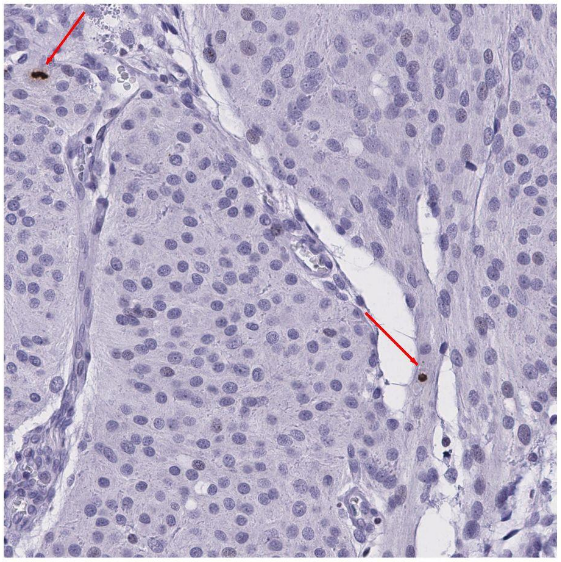

Red arrow: mitosis

H&E (1HPF, 0.16mm^2)

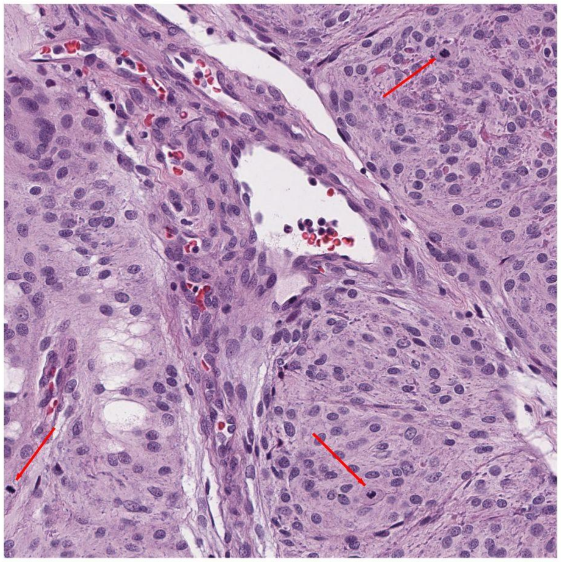

Phosphorylated Histone H3 (0.16mm^2)

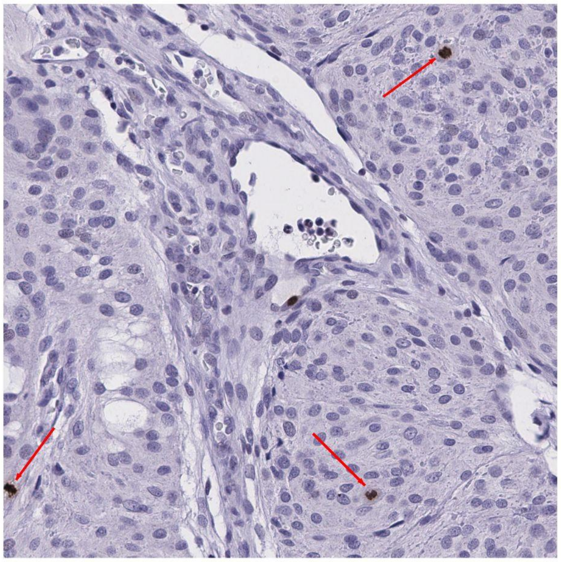

Red arrow: mitosis

H&E (1HPF, 0.16mm^2)

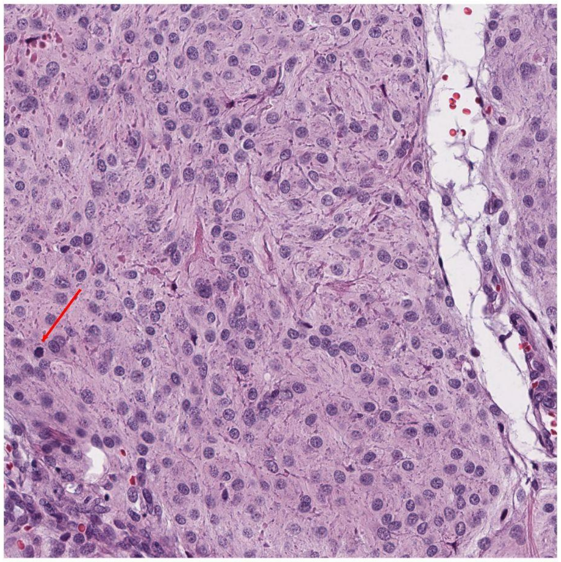

Phosphorylated Histone H3 (0.16mm^2)

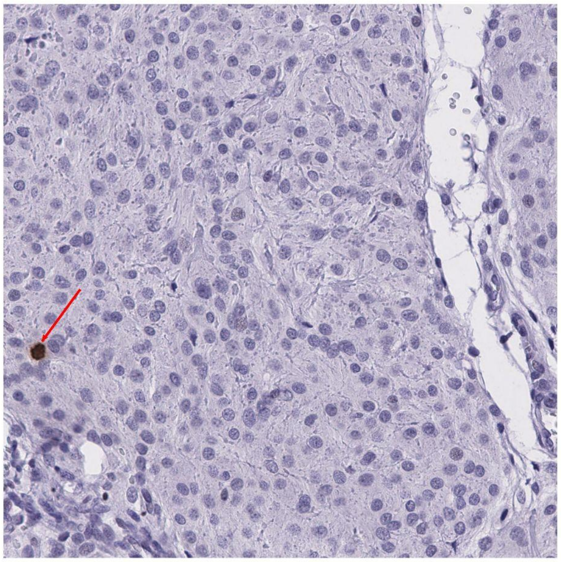

Red arrow: mitosis

H&E (1HPF, 0.16mm^2)

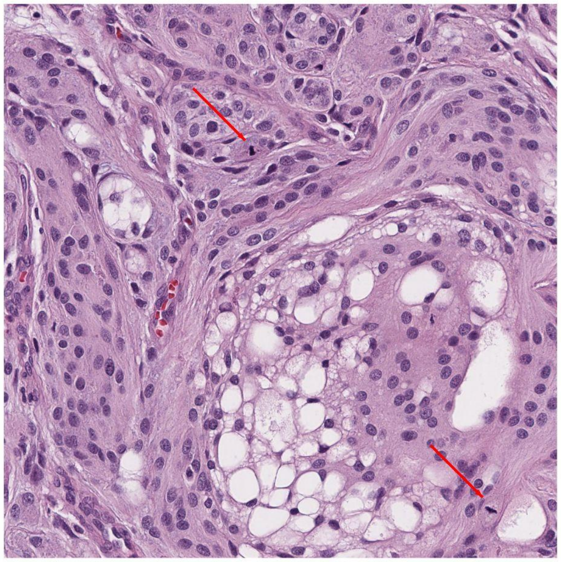

Phosphorylated Histone H3 (0.16mm^2)

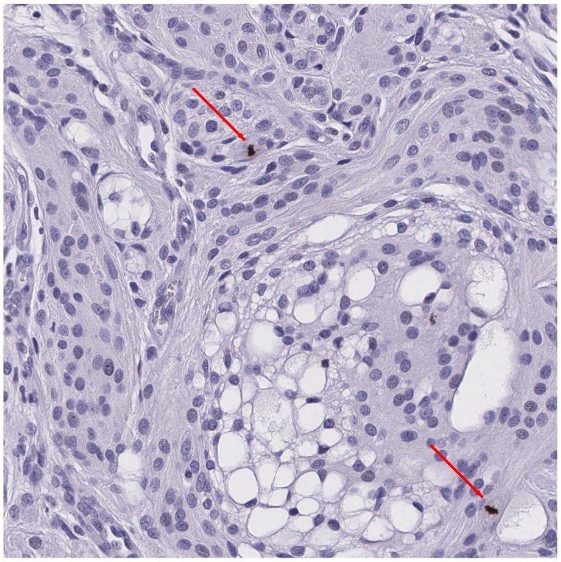

Red arrow: mitosis

H&E (1HPF, 0.16mm^2)

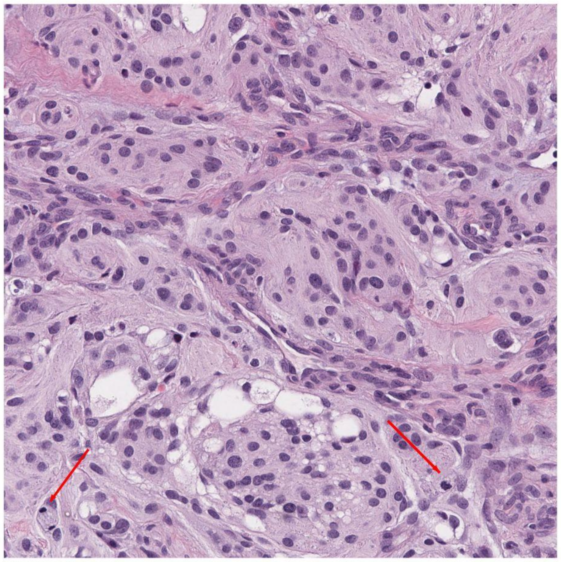

Phosphorylated Histone H3 (0.16mm^2)

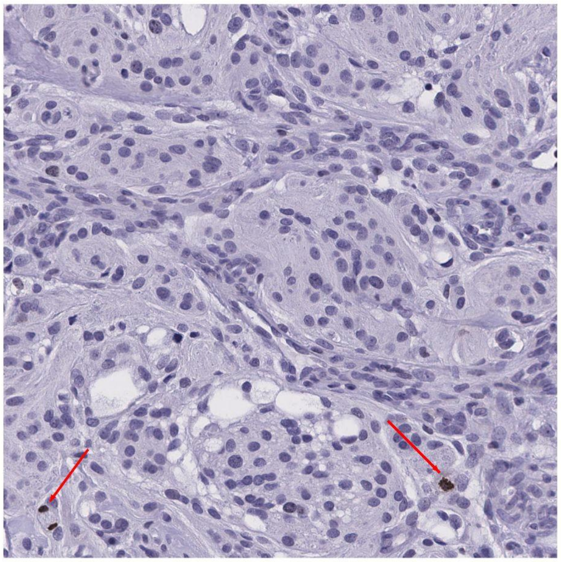

Red arrow: mitosis

H&E (1HPF, 0.16mm^2)

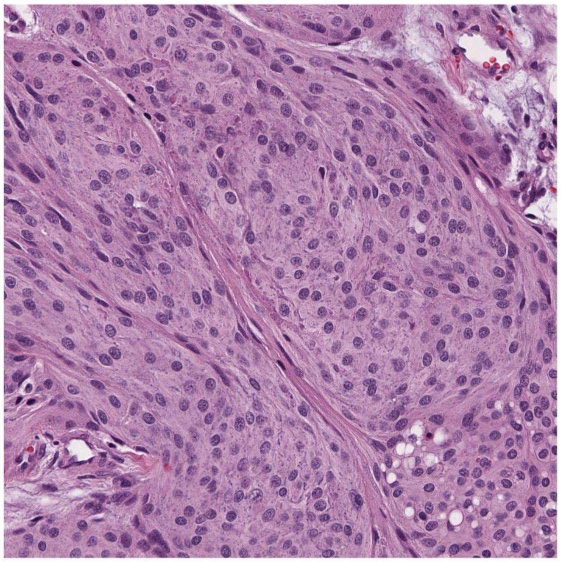

Phosphorylated Histone H3 (0.16mm^2)

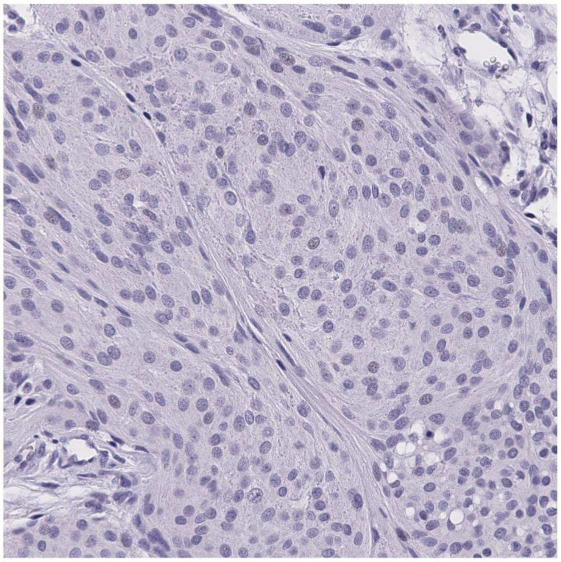

Red arrow: mitosis

H&E (1HPF, 0.16mm^2)

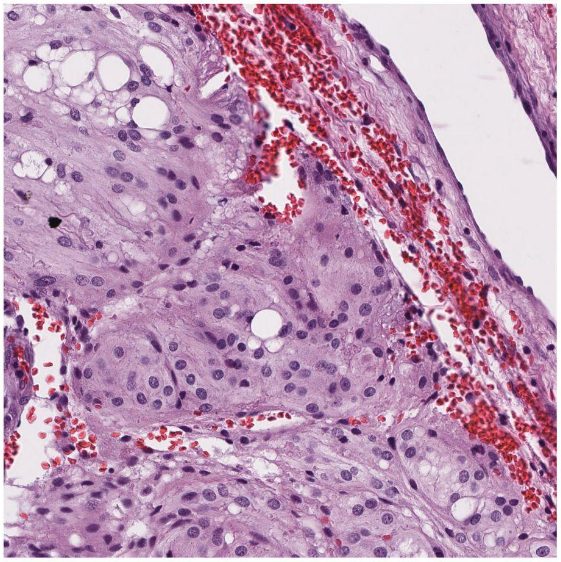

Phosphorylated Histone H3 (0.16mm^2)

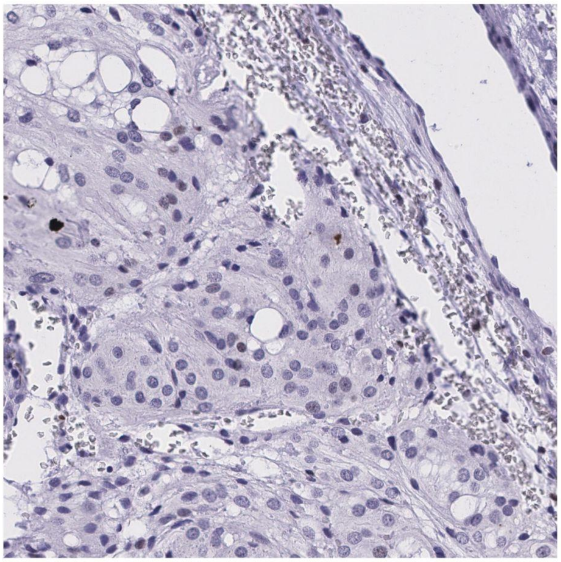

Red arrow: mitosis

H&E (1HPF, 0.16mm^2)

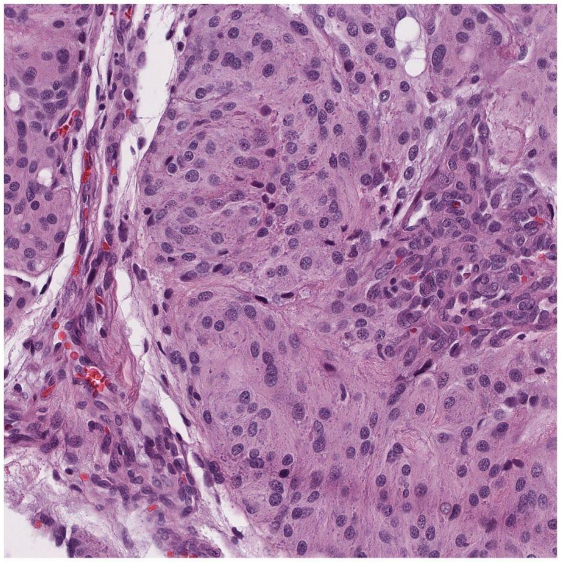

Phosphorylated Histone H3 (0.16mm^2)

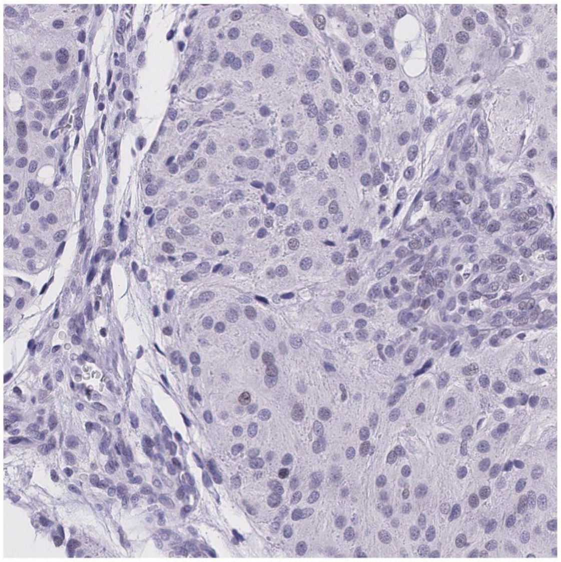

Red arrow: mitosis

H&E (1HPF, 0.16mm^2)

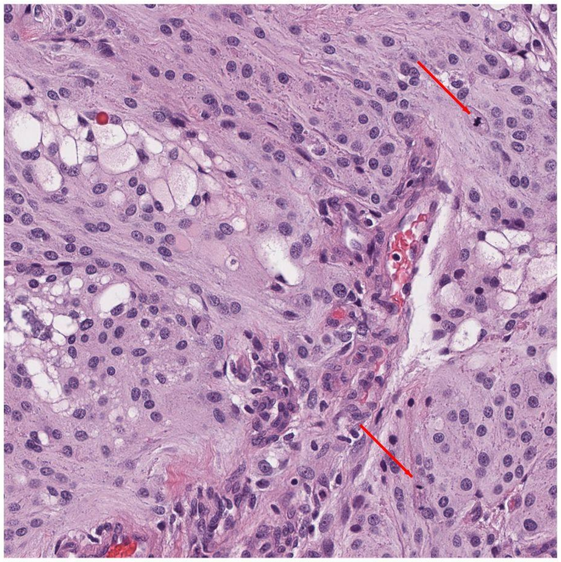

Phosphorylated Histone H3 (0.16mm^2)

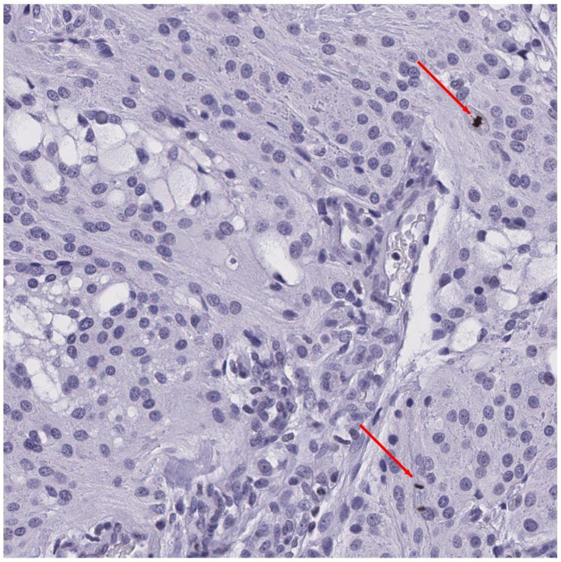

Red arrow: mitosis

H&E (1HPF, 0.16mm^2)

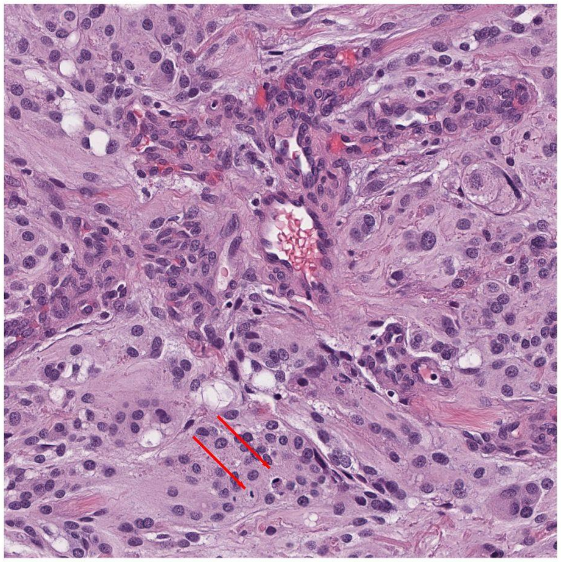

Phosphorylated Histone H3 (0.16mm^2)

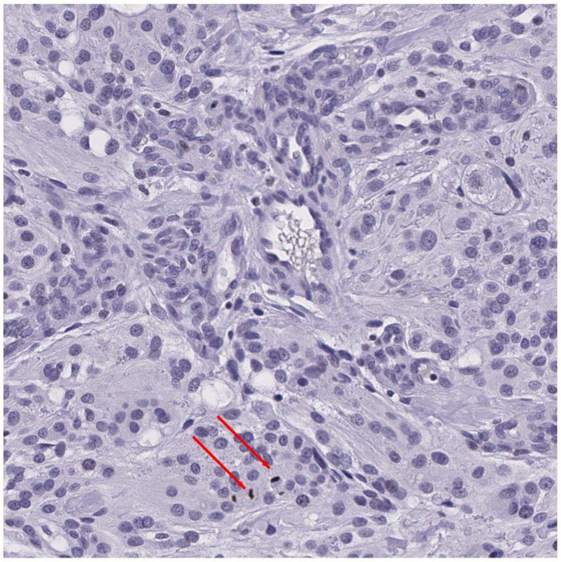

Red arrow: mitosis

H&E (1HPF, 0.16mm^2)

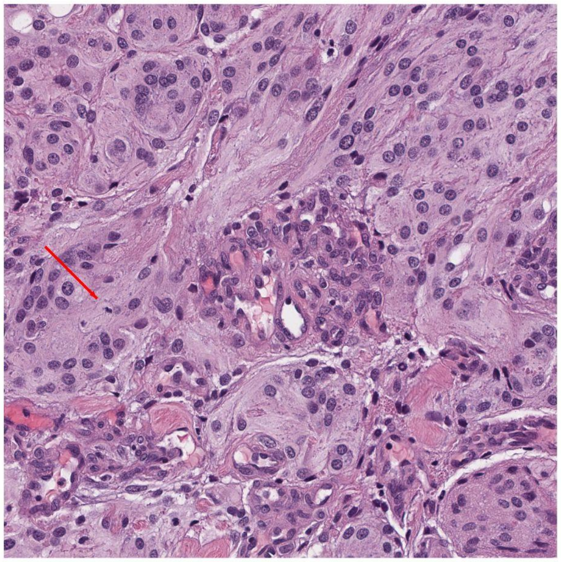

Phosphorylated Histone H3 (0.16mm^2)

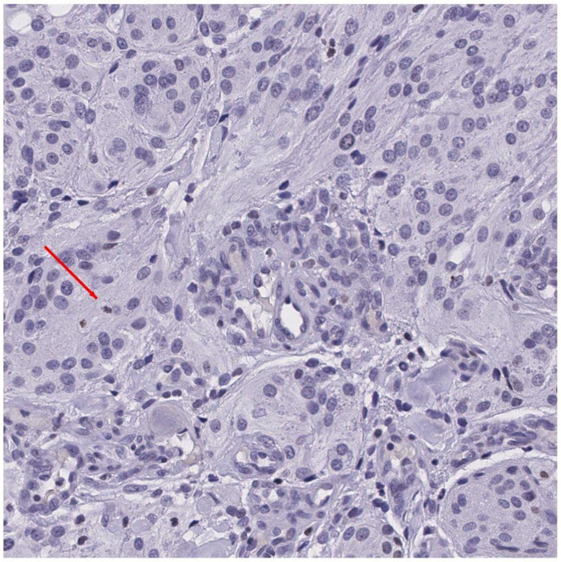

Supplement: Supplementary file 4 — Additional file 4: Mitosis annotations (ground truth) of the 48 HPF images in the user study. [file 40478_2023_1707_MOESM4_ESM.pdf]
