# Supplementary material for: Enhancing mitosis quantification and detection in meningiomas with computational digital pathology
Source: Acta Neuropathol Commun. 2024 Jan 11;12:7. doi: 10.1186/s40478-023-01707-6 (PMC10782692; doi:10.1186/s40478-023-01707-6)

Participant ID: d275b5c4-be08-4907-a032-62c6adfb8a34

Your Result: TP=67, FP=7, FN=21, Precision=0.905, Recall/Sensitivity=0.761, F1=0.827

Time to Finish: 20 minutes 11 seconds

Nomenclature

- Ground truth: mitotic events on H&E after referencing Phosphorylated Histone H3 (PHH3) IHC staining;
- TP: true positive, you labeled a ground truth mitosis correctly;
- FP: false positive, no ground truth mitosis is within 15μm of your label;
- FN: false negative, you did not label within 15μm of a ground truth mitosis;
- Recall/Sensitivity: TP / (TP + FN)
- Precision: TP / (TP + FP)
- F1: 2 * Precision * Recall / (Precision + Recall)

Green arrow: correct (True Positive)
Red arrow: missed mitosis (False Negative)
Blue arrow: wrong label (False Positive)

H&E (1HPF, 0.16mm^2)

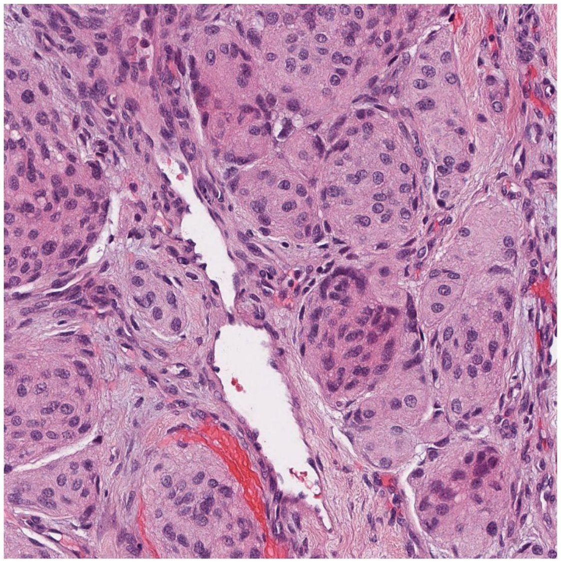

Phosphorylated Histone H3 (0.16mm^2)

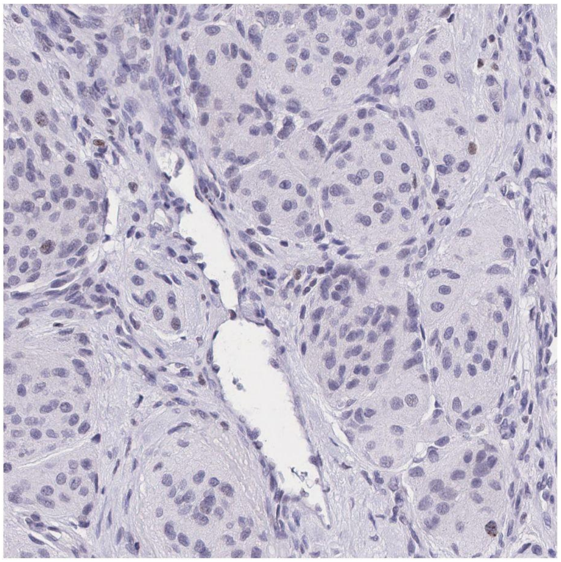

Green arrow: correct (True Positive)
Red arrow: missed mitosis (False Negative)
Blue arrow: wrong label (False Positive)

H&E (1HPF, 0.16mm^2)

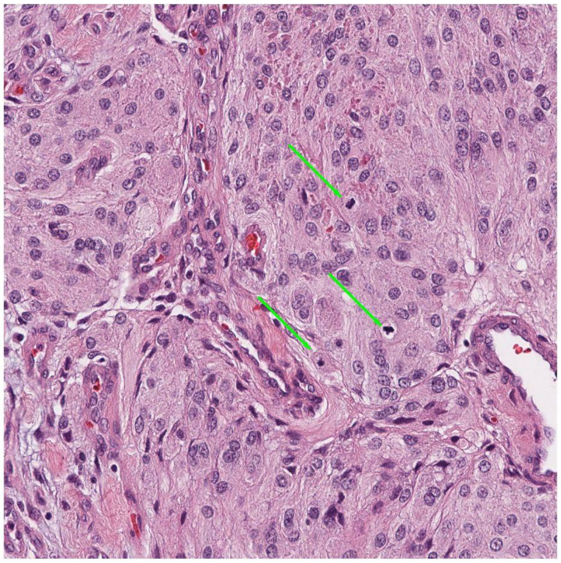

Phosphorylated Histone H3 (0.16mm^2)

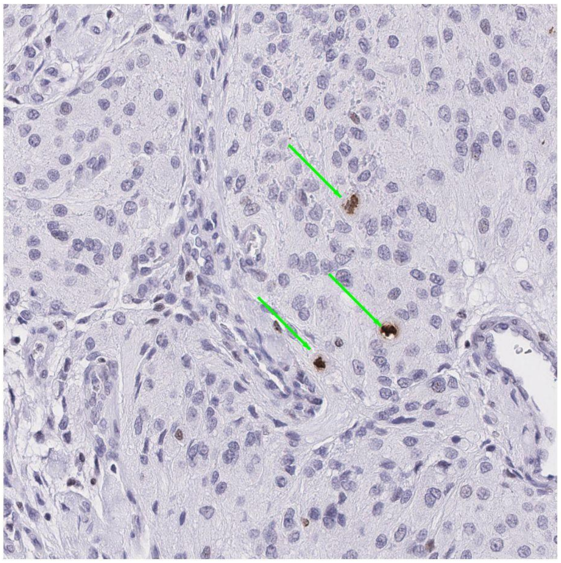

Green arrow: correct (True Positive)
Red arrow: missed mitosis (False Negative)
Blue arrow: wrong label (False Positive)

H&E (1HPF, $0.16mm^2$)

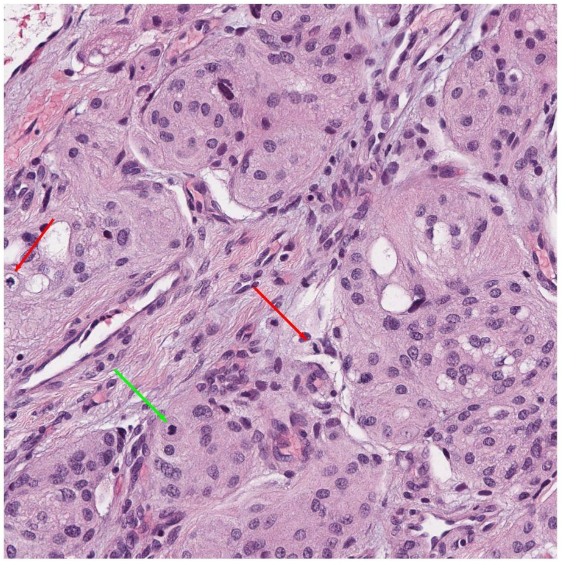

Phosphorylated Histone H3 ($0.16mm^2$)

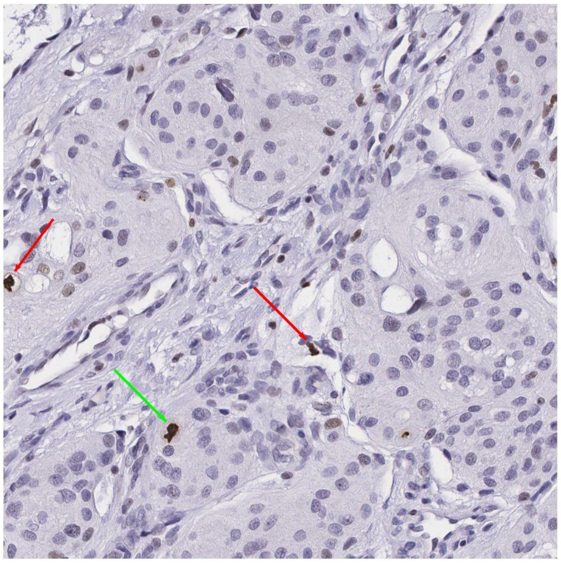

Green arrow: correct (True Positive)
Red arrow: missed mitosis (False Negative)
Blue arrow: wrong label (False Positive)

H&E (1HPF, $0.16mm^2$)

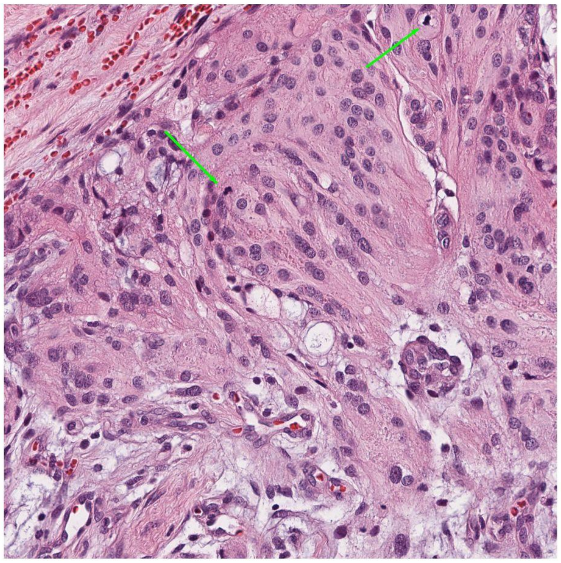

Phosphorylated Histone H3 ($0.16mm^2$)

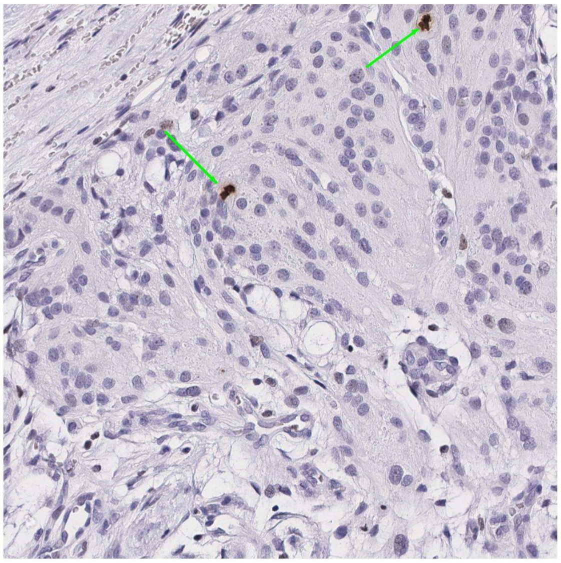

Green arrow: correct (True Positive)
Red arrow: missed mitosis (False Negative)
Blue arrow: wrong label (False Positive)

H&E (1HPF, $0.16mm^2$)

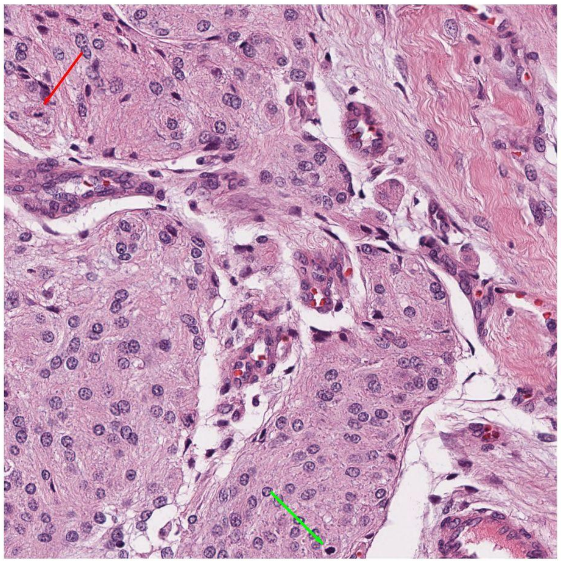

Phosphorylated Histone H3 ($0.16mm^2$)

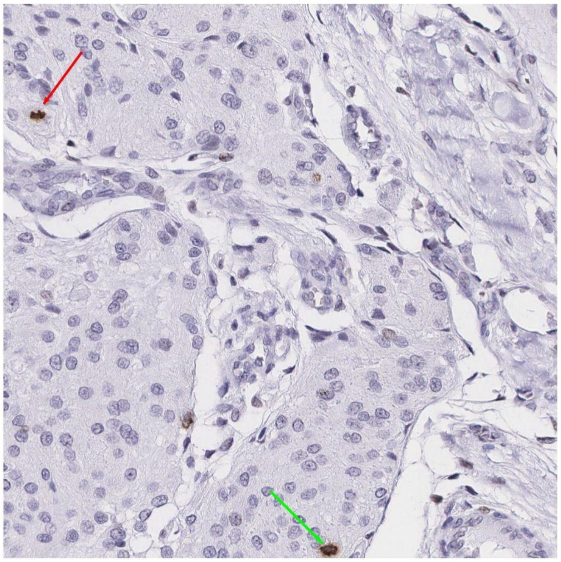

Green arrow: correct (True Positive)
Red arrow: missed mitosis (False Negative)
Blue arrow: wrong label (False Positive)

H&E (1HPF, $0.16mm^2$)

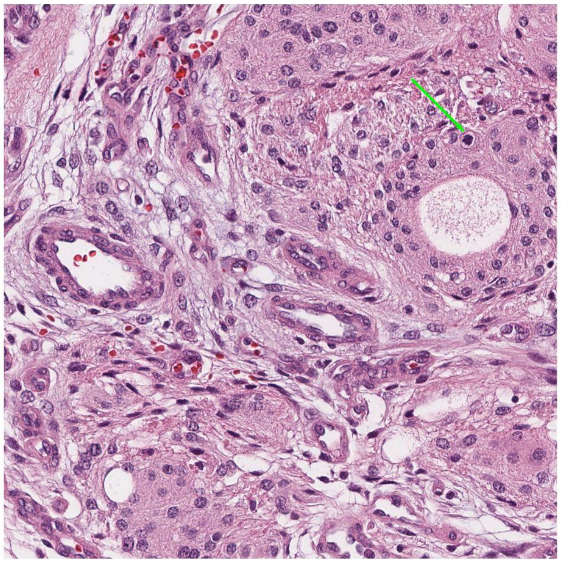

Phosphorylated Histone H3 ($0.16mm^2$)

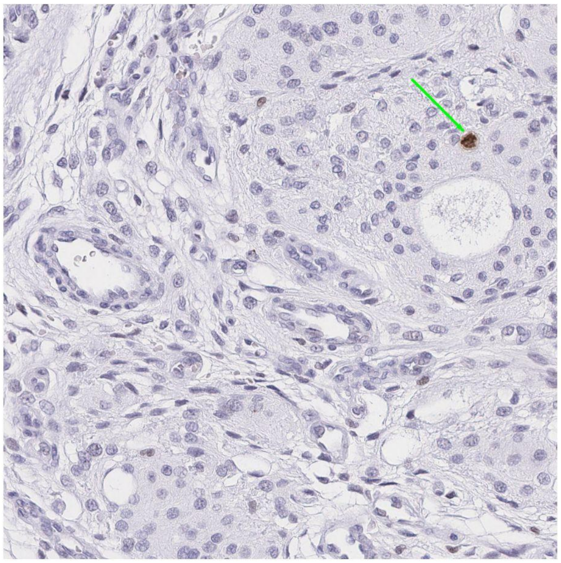

Green arrow: correct (True Positive)
Red arrow: missed mitosis (False Negative)
Blue arrow: wrong label (False Positive)

H&E (1HPF, $0.16mm^2$)

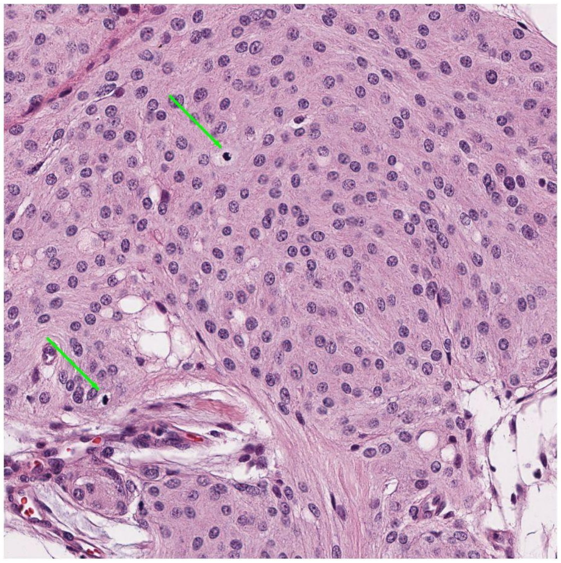

Phosphorylated Histone H3 ($0.16mm^2$)

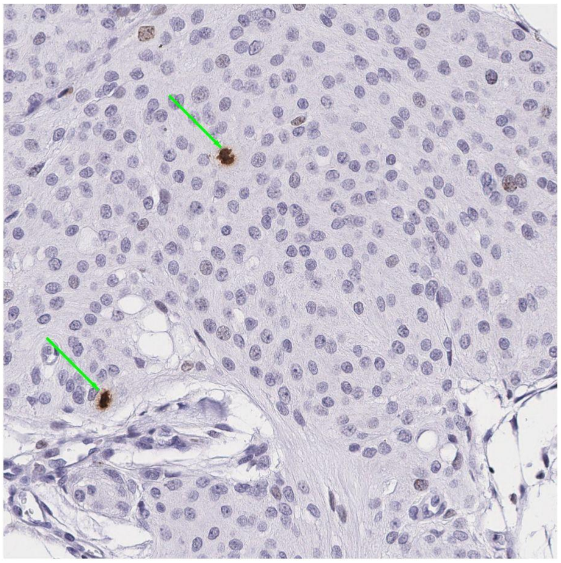

Green arrow: correct (True Positive)
Red arrow: missed mitosis (False Negative)
Blue arrow: wrong label (False Positive)

H&E (1HPF, $0.16mm^2$)

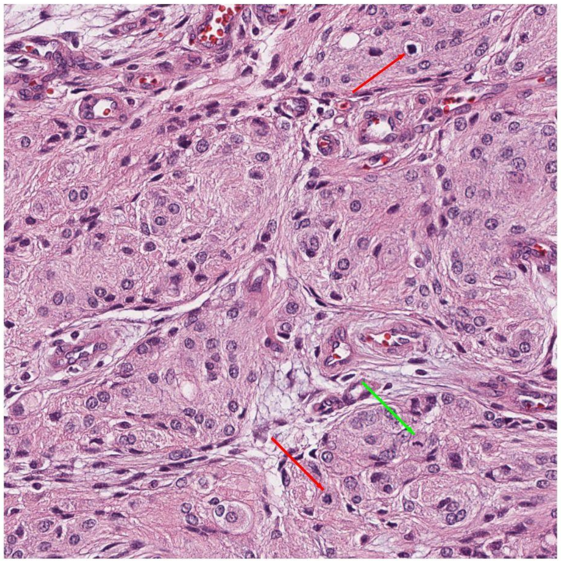

Phosphorylated Histone H3 ($0.16mm^2$)

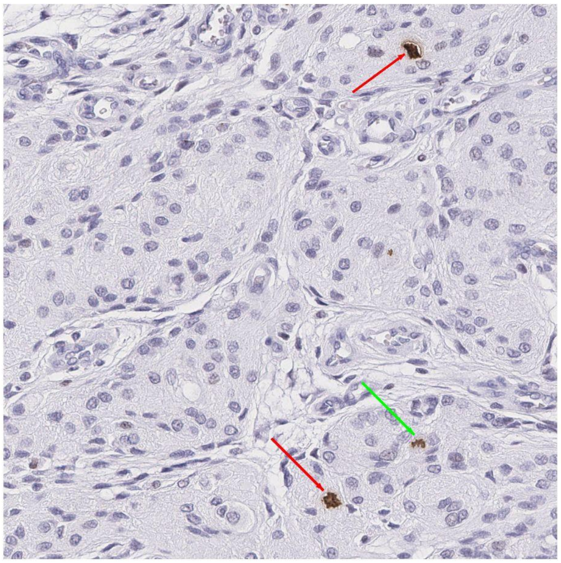

Green arrow: correct (True Positive)
Red arrow: missed mitosis (False Negative)
Blue arrow: wrong label (False Positive)

H&E (1HPF, $0.16mm^2$)

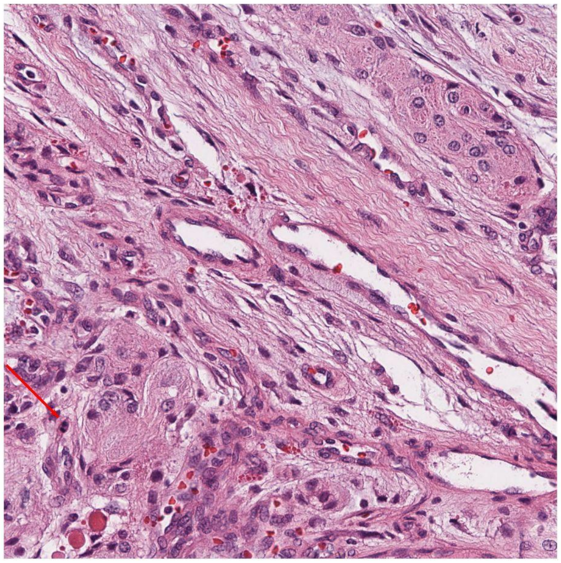

Phosphorylated Histone H3 ($0.16mm^2$)

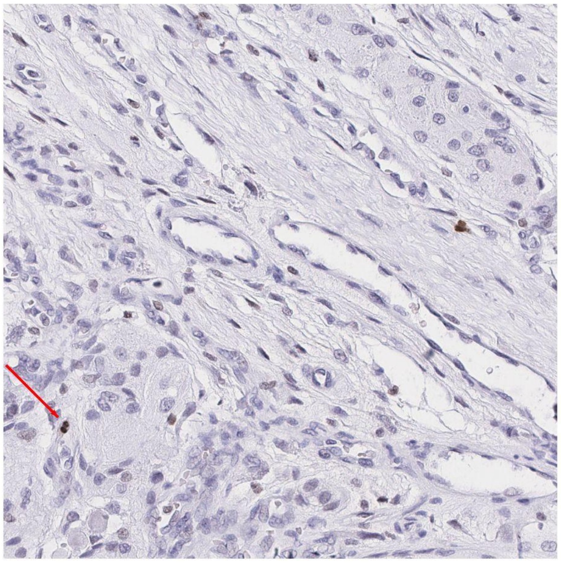

Green arrow: correct (True Positive)
Red arrow: missed mitosis (False Negative)
Blue arrow: wrong label (False Positive)

H&E (1HPF, $0.16mm^2$)

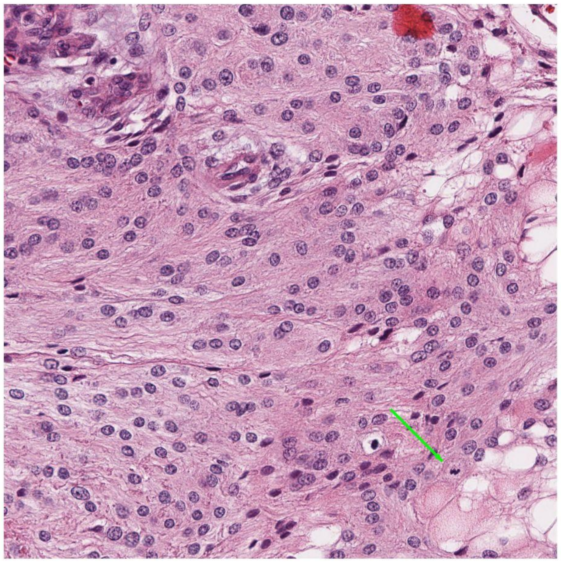

Phosphorylated Histone H3 ($0.16mm^2$)

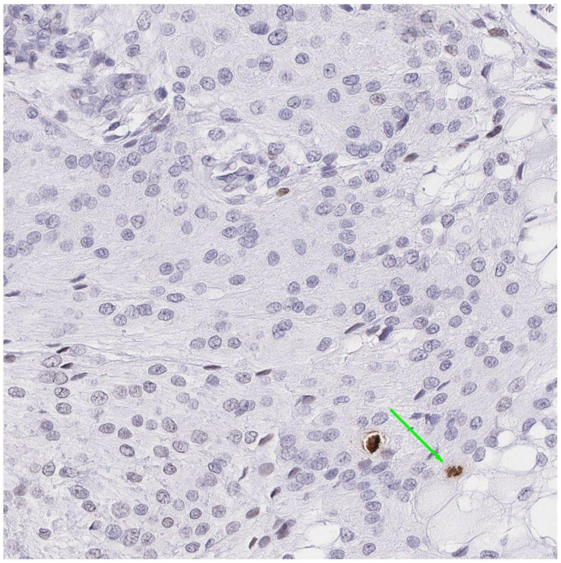

Green arrow: correct (True Positive)
Red arrow: missed mitosis (False Negative)
Blue arrow: wrong label (False Positive)

H&E (1HPF, 0.16mm^2)

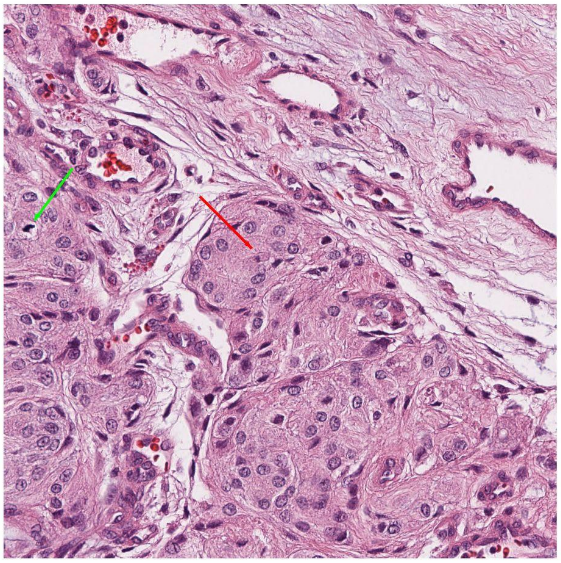

Phosphorylated Histone H3 (0.16mm^2)

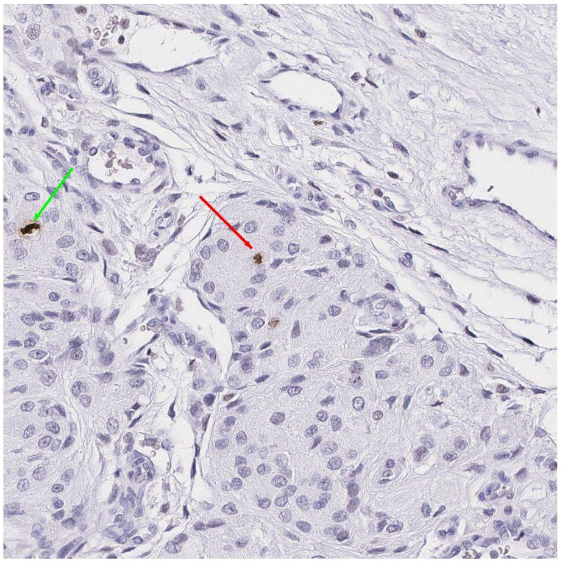

Green arrow: correct (True Positive)
Red arrow: missed mitosis (False Negative)
Blue arrow: wrong label (False Positive)

H&E (1HPF, 0.16mm^2)

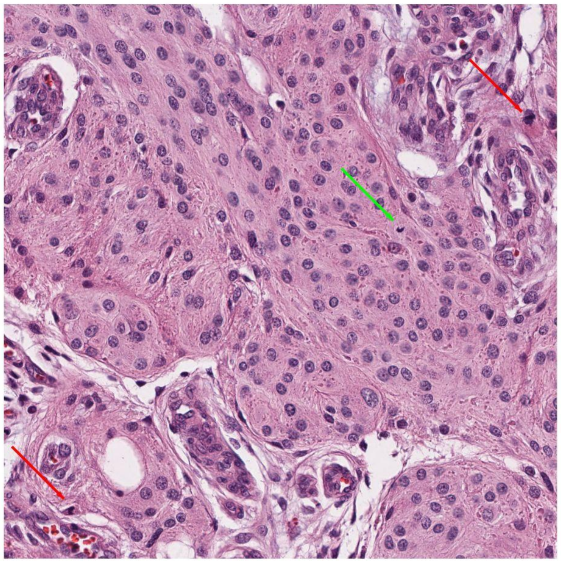

Phosphorylated Histone H3 (0.16mm^2)

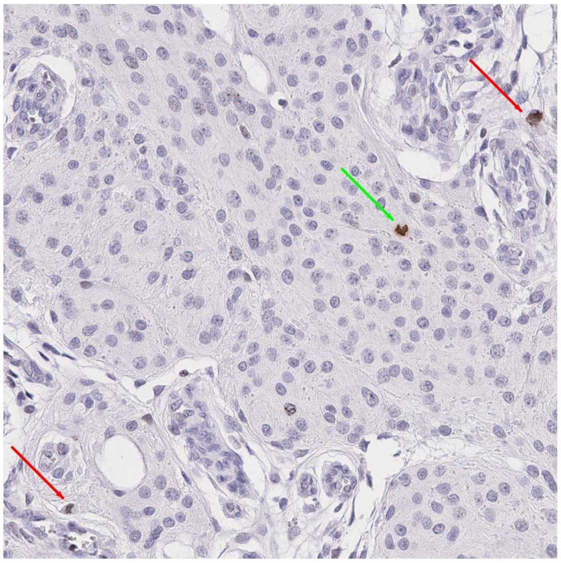

Green arrow: correct (True Positive)
Red arrow: missed mitosis (False Negative)
Blue arrow: wrong label (False Positive)

H&E (1HPF, 0.16mm^2)

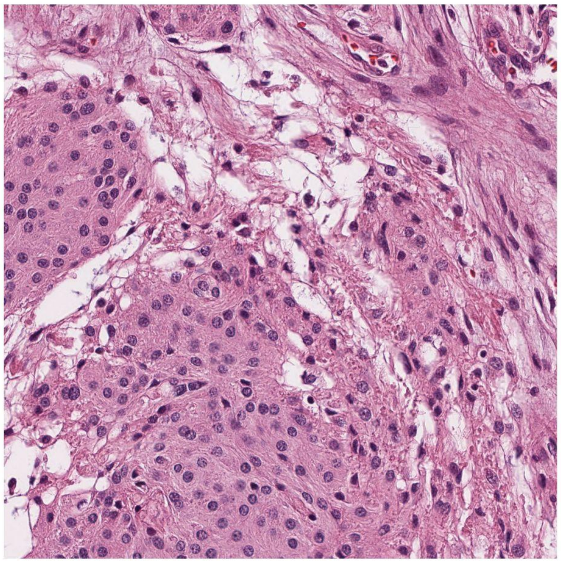

Phosphorylated Histone H3 (0.16mm^2)

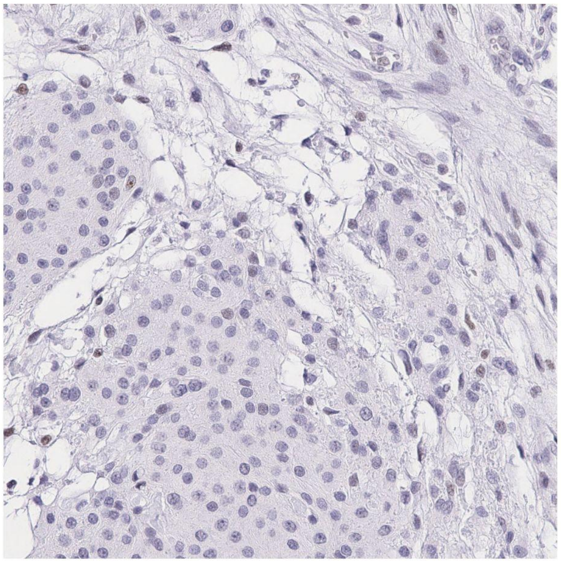

Green arrow: correct (True Positive)
Red arrow: missed mitosis (False Negative)
Blue arrow: wrong label (False Positive)

H&E (1HPF, 0.16mm^2)

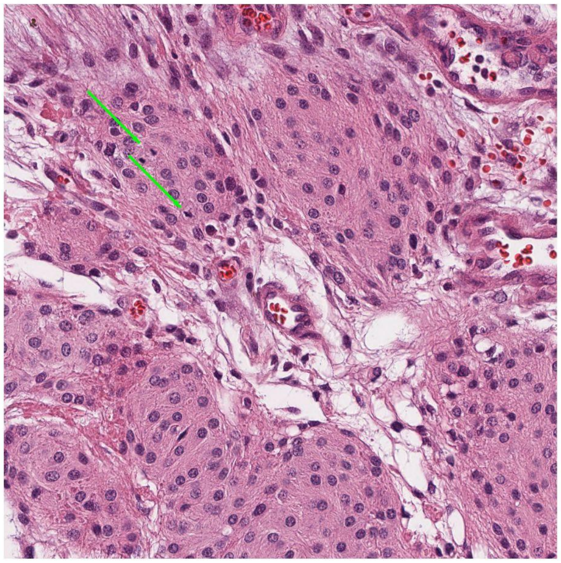

Phosphorylated Histone H3 (0.16mm^2)

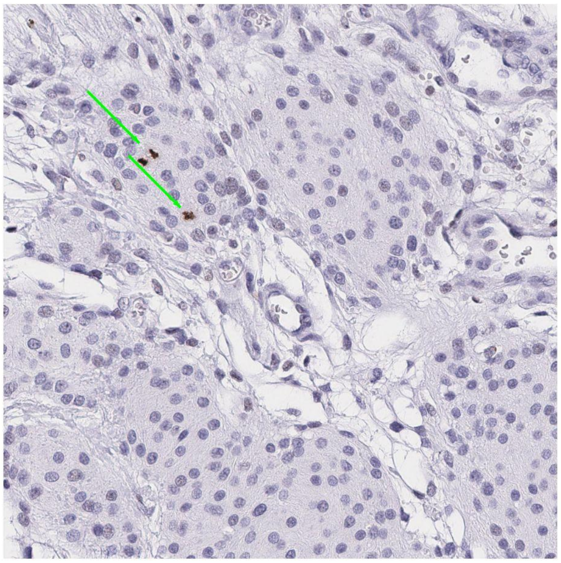

Green arrow: correct (True Positive)
Red arrow: missed mitosis (False Negative)
Blue arrow: wrong label (False Positive)

H&E (1HPF, 0.16mm^2)

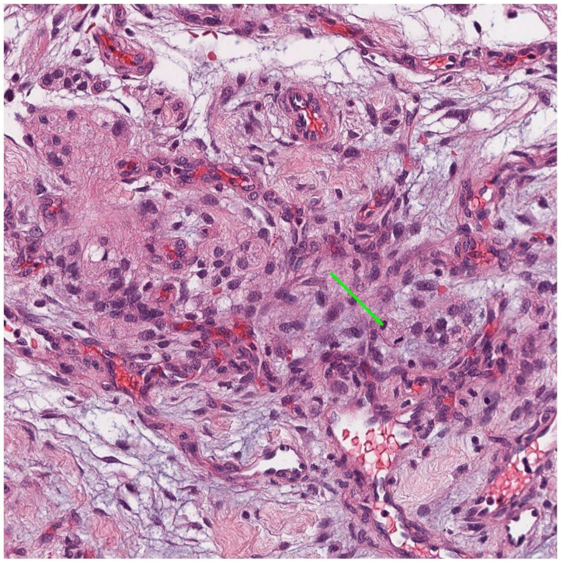

Phosphorylated Histone H3 (0.16mm^2)

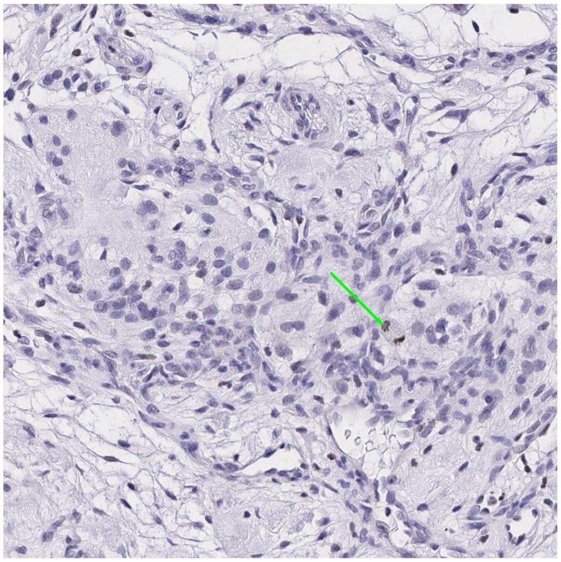

Green arrow: correct (True Positive)
Red arrow: missed mitosis (False Negative)
Blue arrow: wrong label (False Positive)

H&E (1HPF, 0.16mm^2)

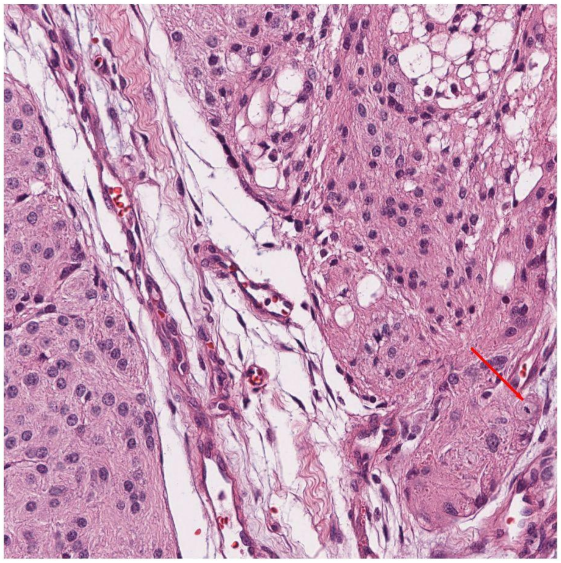

Phosphorylated Histone H3 (0.16mm^2)

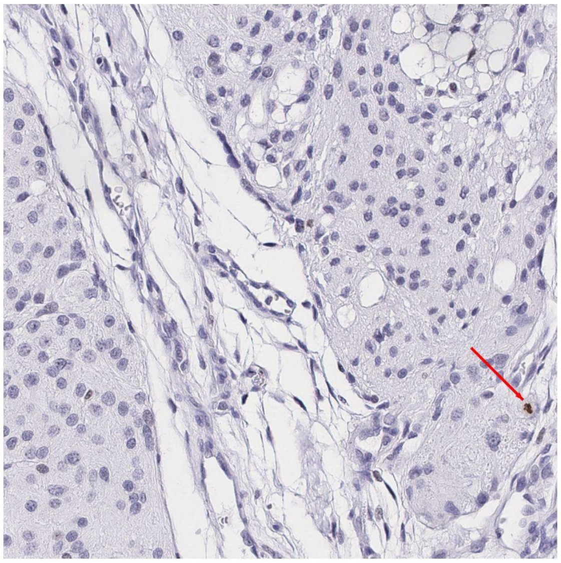

Green arrow: correct (True Positive)
Red arrow: missed mitosis (False Negative)
Blue arrow: wrong label (False Positive)

H&E (1HPF, 0.16mm^2)

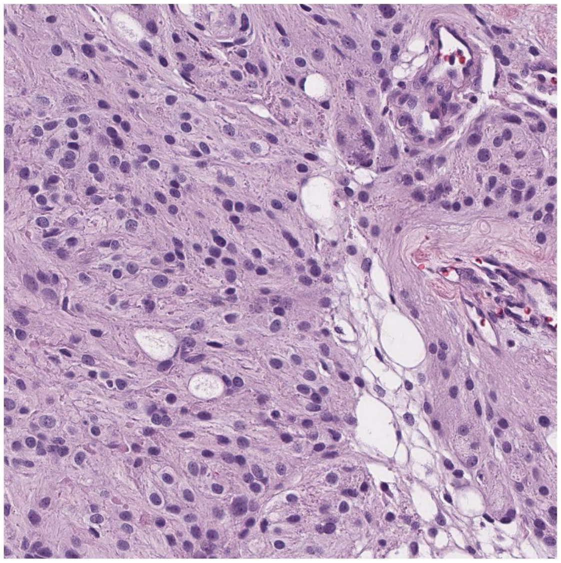

Phosphorylated Histone H3 (0.16mm^2)

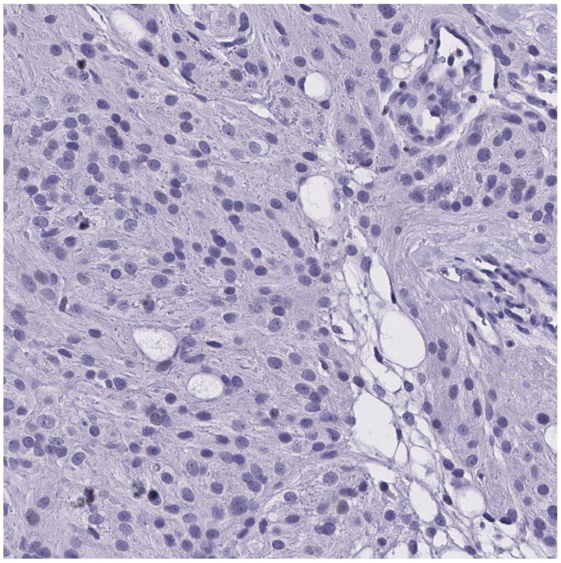

Green arrow: correct (True Positive)
Red arrow: missed mitosis (False Negative)
Blue arrow: wrong label (False Positive)

H&E (1HPF, 0.16mm^2)

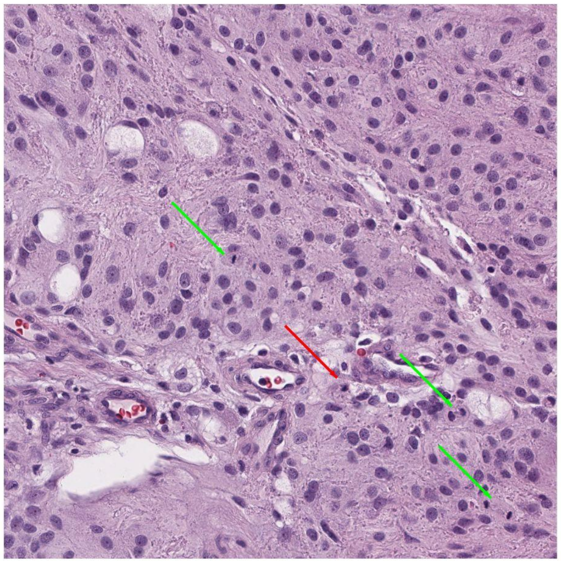

Phosphorylated Histone H3 (0.16mm^2)

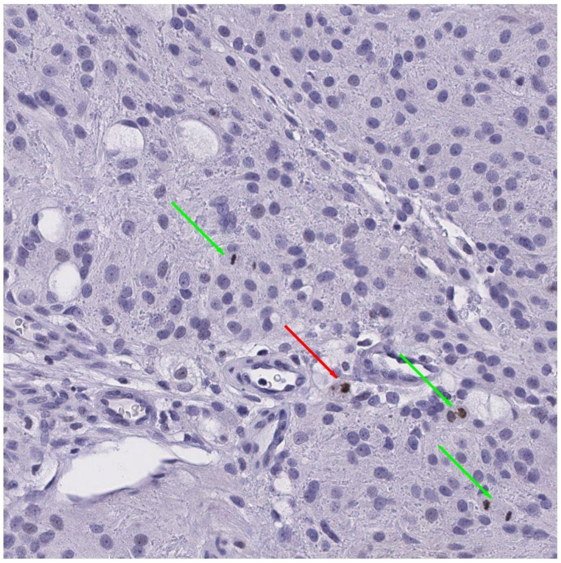

Green arrow: correct (True Positive)
Red arrow: missed mitosis (False Negative)
Blue arrow: wrong label (False Positive)

H&E (1HPF, $0.16mm^2$)

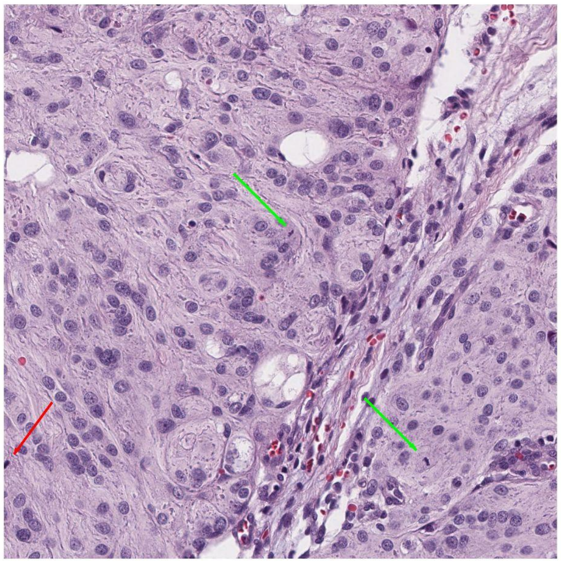

Phosphorylated Histone H3 ($0.16mm^2$)

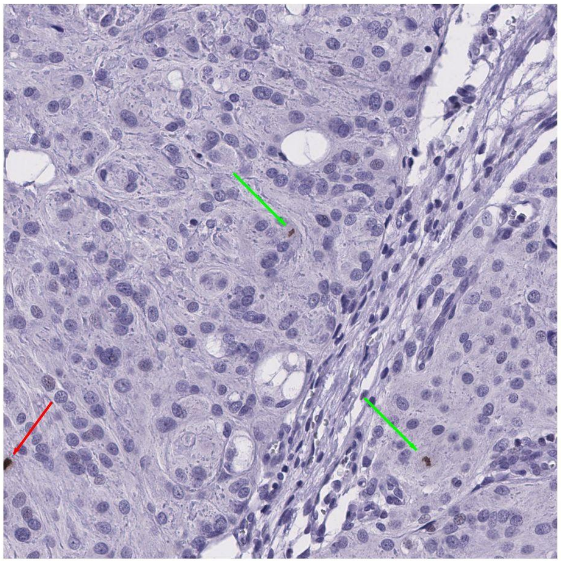

Green arrow: correct (True Positive)
Red arrow: missed mitosis (False Negative)
Blue arrow: wrong label (False Positive)

H&E (1HPF, $0.16mm^2$)

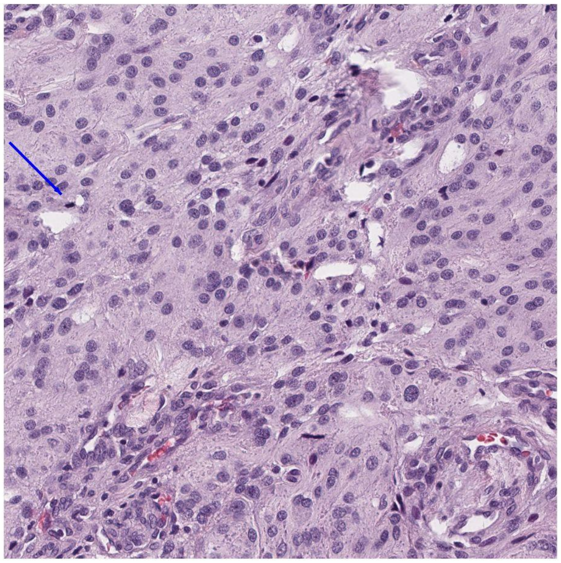

Phosphorylated Histone H3 ($0.16mm^2$)

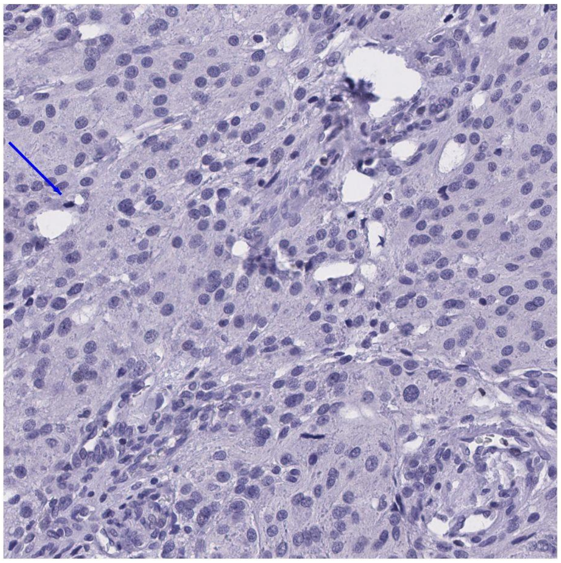

Green arrow: correct (True Positive)
Red arrow: missed mitosis (False Negative)
Blue arrow: wrong label (False Positive)

H&E (1HPF, $0.16mm^2$)

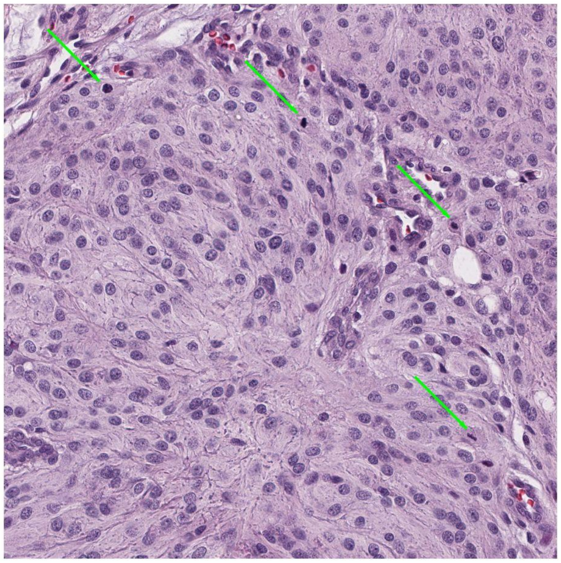

Phosphorylated Histone H3 ($0.16mm^2$)

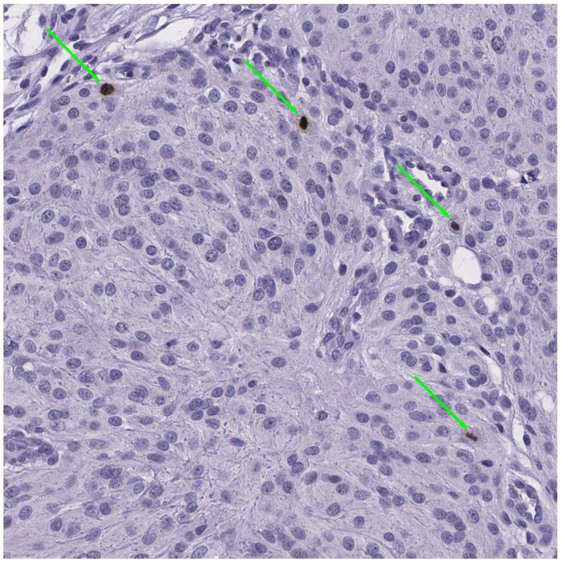

Green arrow: correct (True Positive)
Red arrow: missed mitosis (False Negative)
Blue arrow: wrong label (False Positive)

H&E (1HPF, $0.16mm^2$)

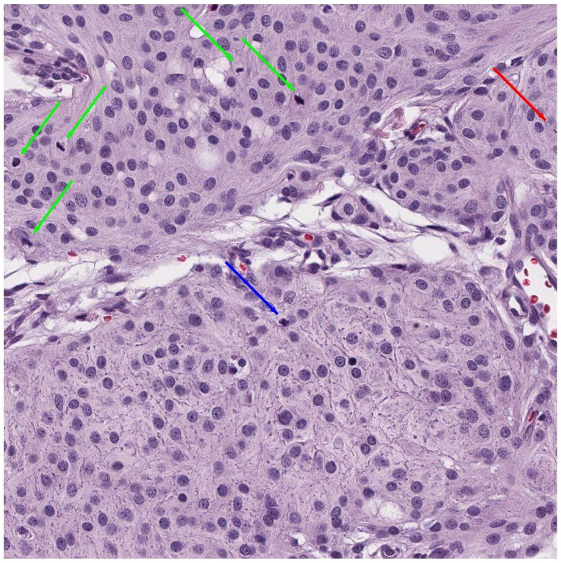

Phosphorylated Histone H3 ($0.16mm^2$)

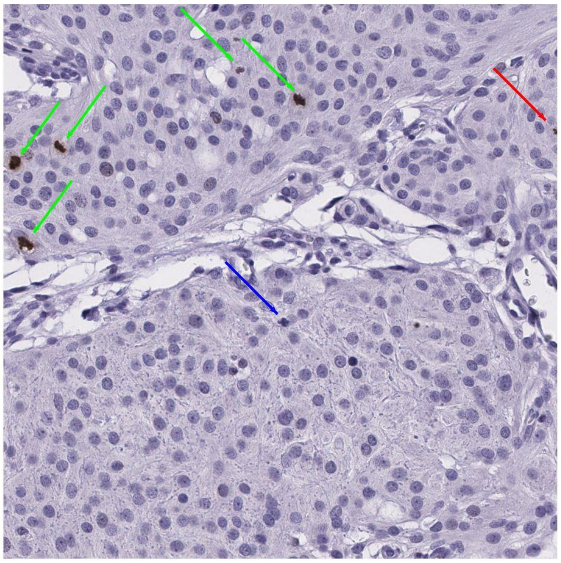

Green arrow: correct (True Positive)
Red arrow: missed mitosis (False Negative)
Blue arrow: wrong label (False Positive)

H&E (1HPF, $0.16mm^2$)

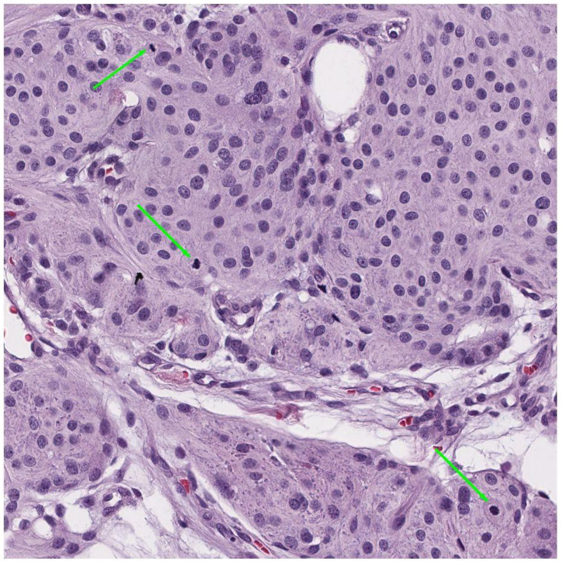

Phosphorylated Histone H3 ($0.16mm^2$)

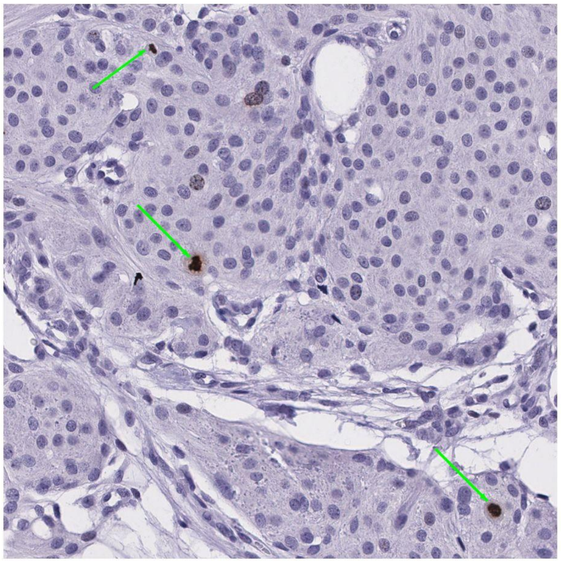

Green arrow: correct (True Positive)
Red arrow: missed mitosis (False Negative)
Blue arrow: wrong label (False Positive)

H&E (1HPF, $0.16mm^2$)

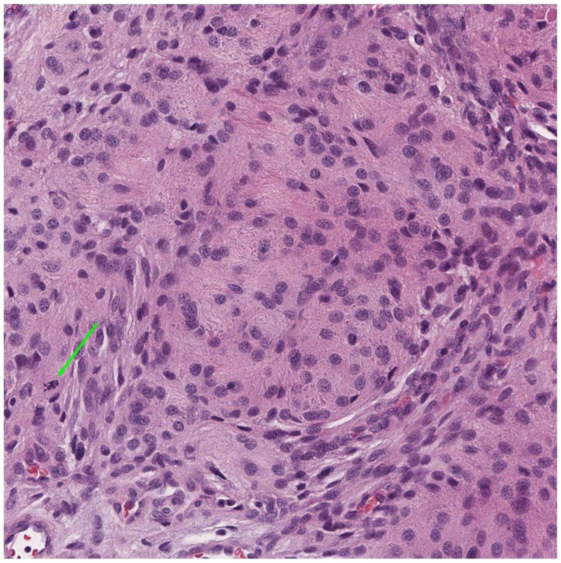

Phosphorylated Histone H3 ($0.16mm^2$)

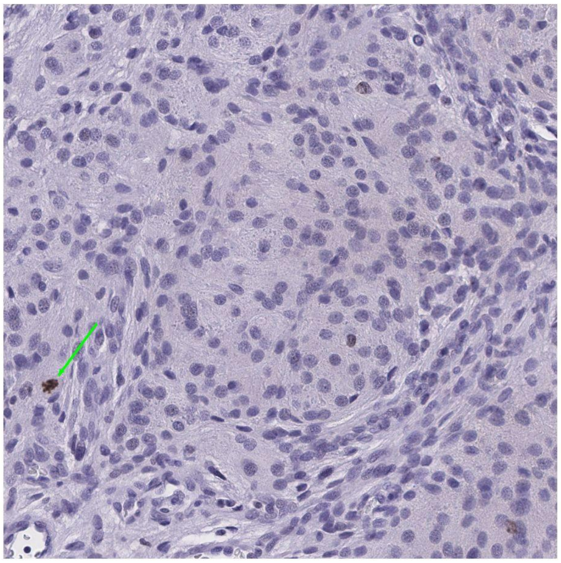

Green arrow: correct (True Positive)
Red arrow: missed mitosis (False Negative)
Blue arrow: wrong label (False Positive)

H&E (1HPF, 0.16mm^2)

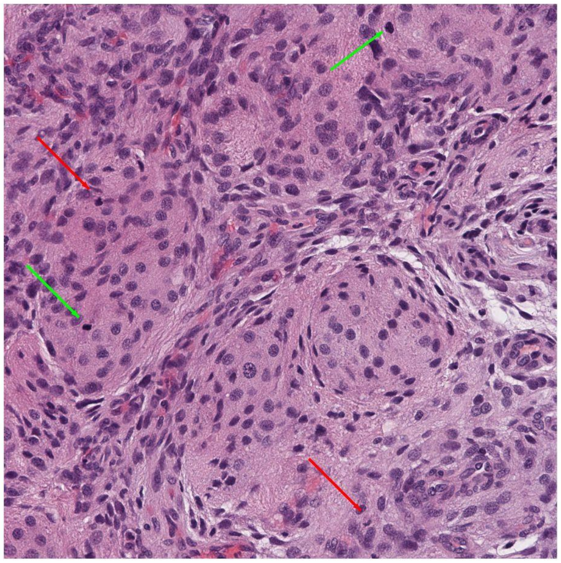

Phosphorylated Histone H3 (0.16mm^2)

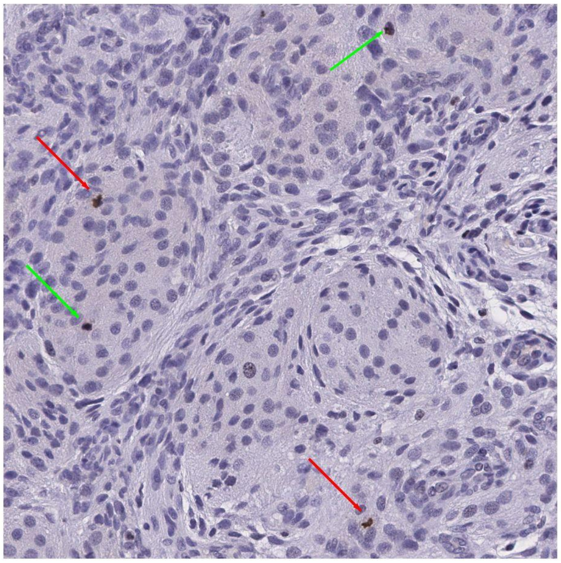

Green arrow: correct (True Positive)
Red arrow: missed mitosis (False Negative)
Blue arrow: wrong label (False Positive)

H&E (1HPF, 0.16mm^2)

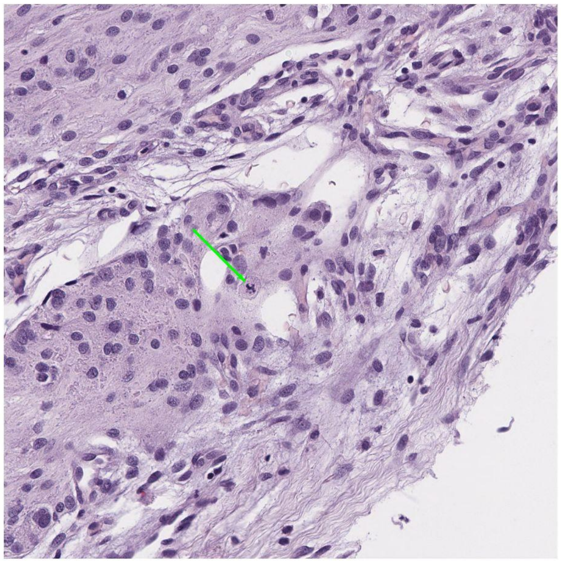

Phosphorylated Histone H3 (0.16mm^2)

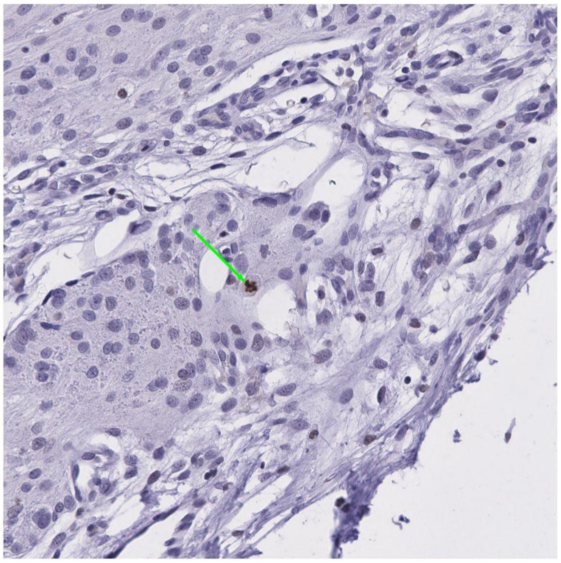

Green arrow: correct (True Positive)
Red arrow: missed mitosis (False Negative)
Blue arrow: wrong label (False Positive)

H&E (1HPF, 0.16mm^2)

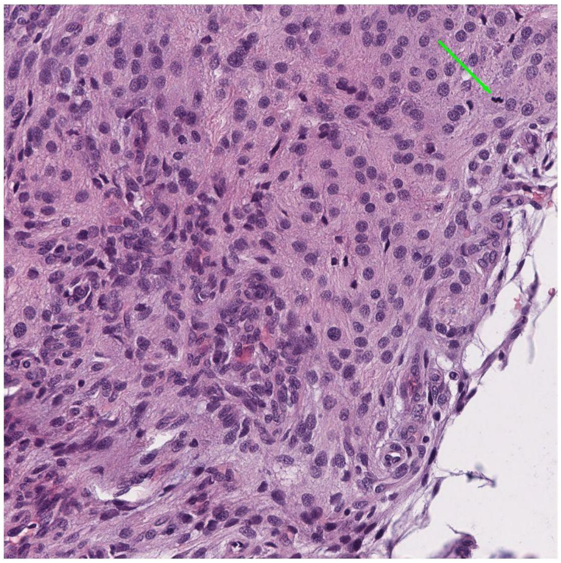

Phosphorylated Histone H3 (0.16mm^2)

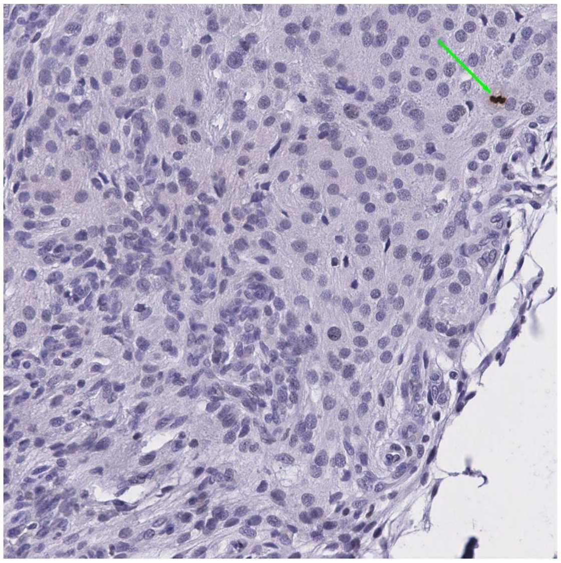

Green arrow: correct (True Positive)
Red arrow: missed mitosis (False Negative)
Blue arrow: wrong label (False Positive)

H&E (1HPF, 0.16mm^2)

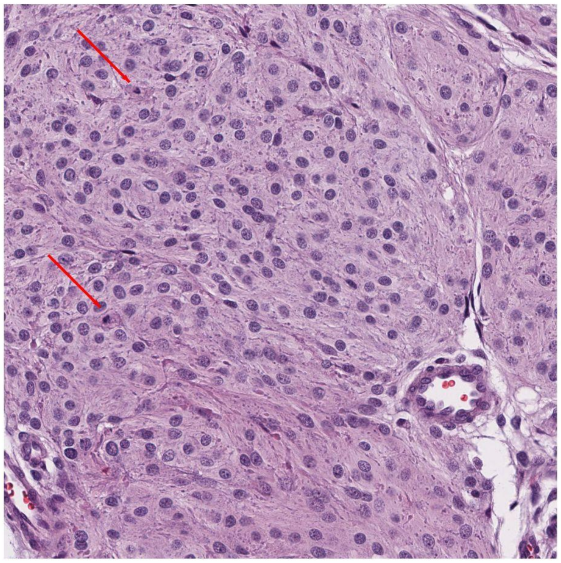

Phosphorylated Histone H3 (0.16mm^2)

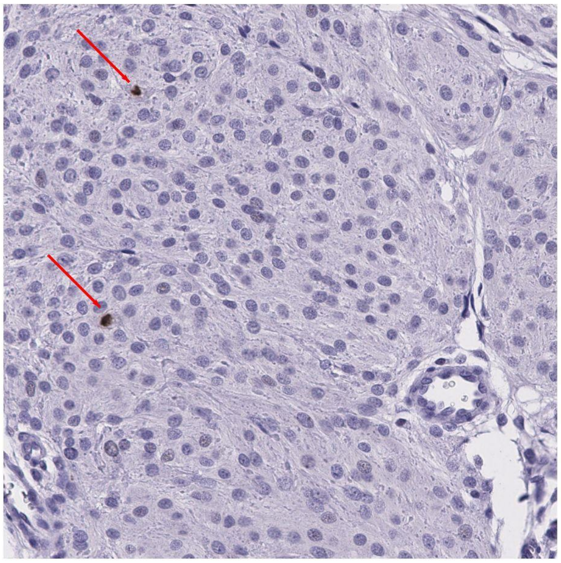

Green arrow: correct (True Positive)
Red arrow: missed mitosis (False Negative)
Blue arrow: wrong label (False Positive)

H&E (1HPF, $0.16mm^2$)

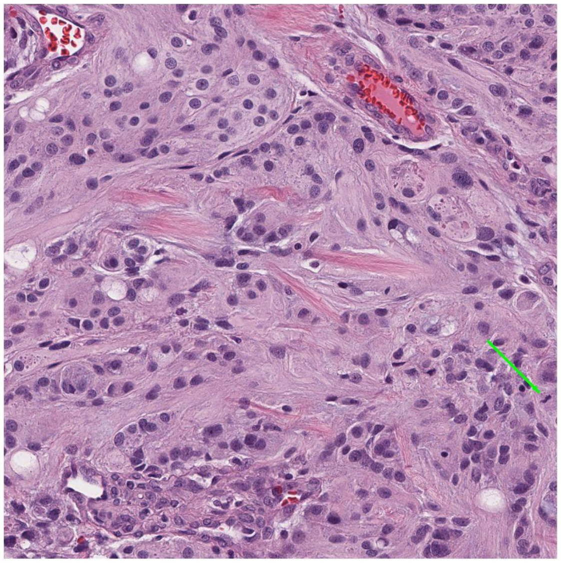

Phosphorylated Histone H3 ($0.16mm^2$)

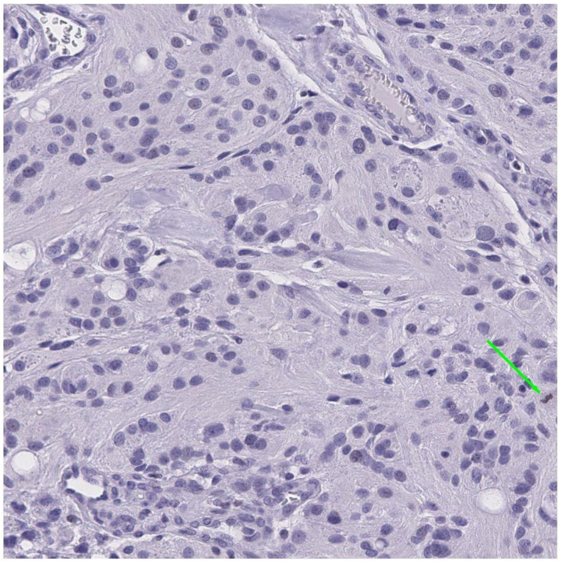

Green arrow: correct (True Positive)
Red arrow: missed mitosis (False Negative)
Blue arrow: wrong label (False Positive)

H&E (1HPF, $0.16mm^2$)

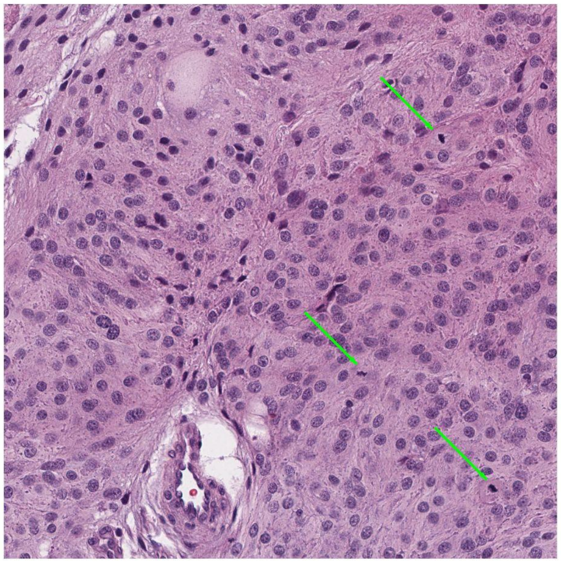

Phosphorylated Histone H3 ($0.16mm^2$)

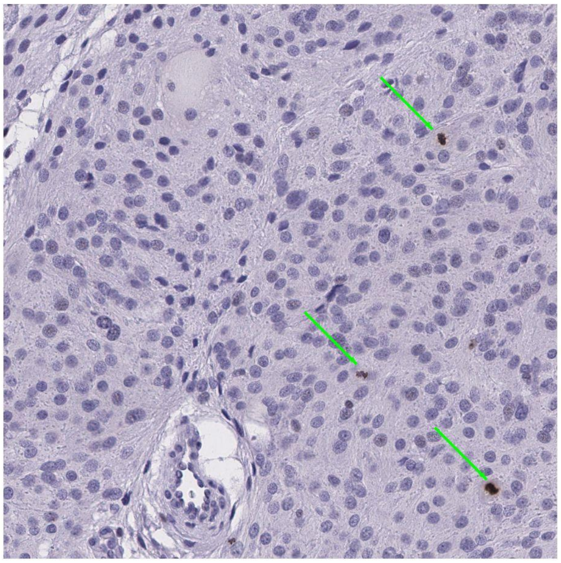

Green arrow: correct (True Positive)
Red arrow: missed mitosis (False Negative)
Blue arrow: wrong label (False Positive)

H&E (1HPF, 0.16mm^2)

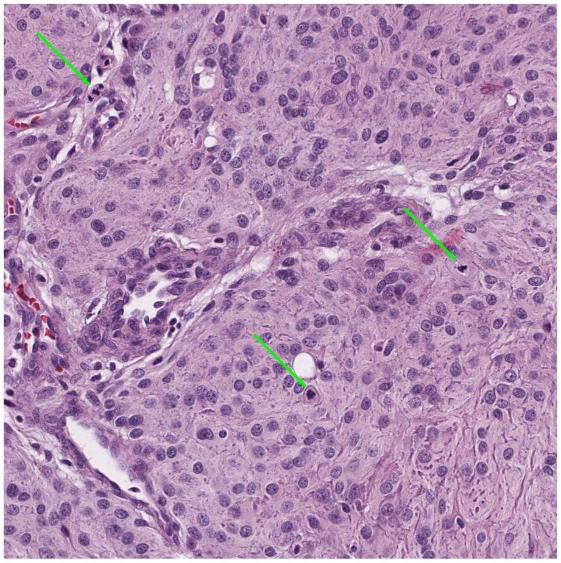

Phosphorylated Histone H3 (0.16mm^2)

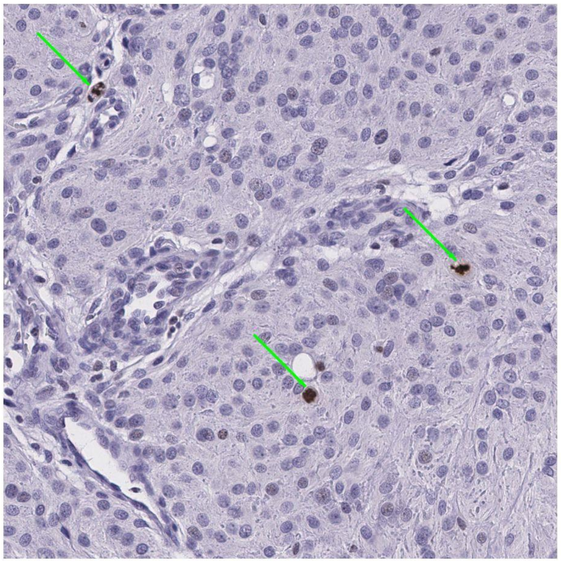

Green arrow: correct (True Positive)
Red arrow: missed mitosis (False Negative)
Blue arrow: wrong label (False Positive)

H&E (1HPF, 0.16mm^2)

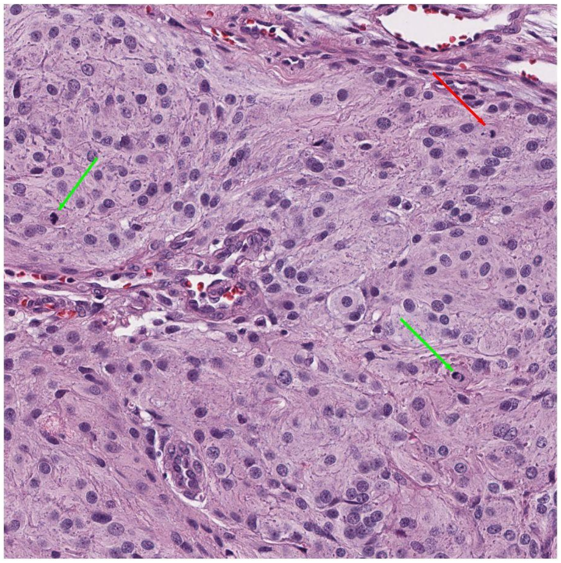

Phosphorylated Histone H3 (0.16mm^2)

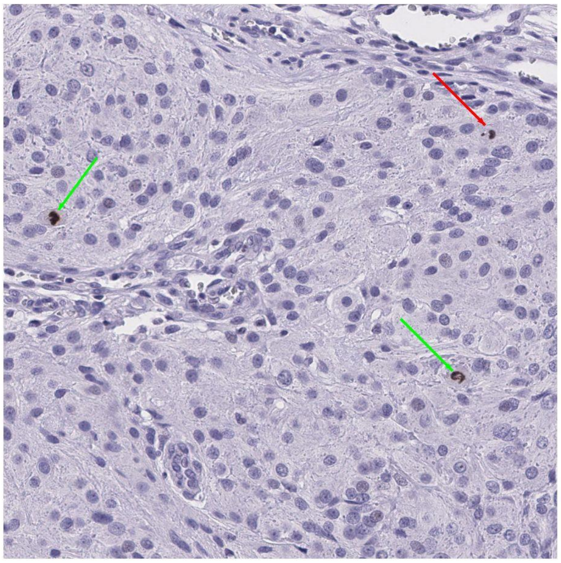

Green arrow: correct (True Positive)
Red arrow: missed mitosis (False Negative)
Blue arrow: wrong label (False Positive)

H&E (1HPF, 0.16mm^2)

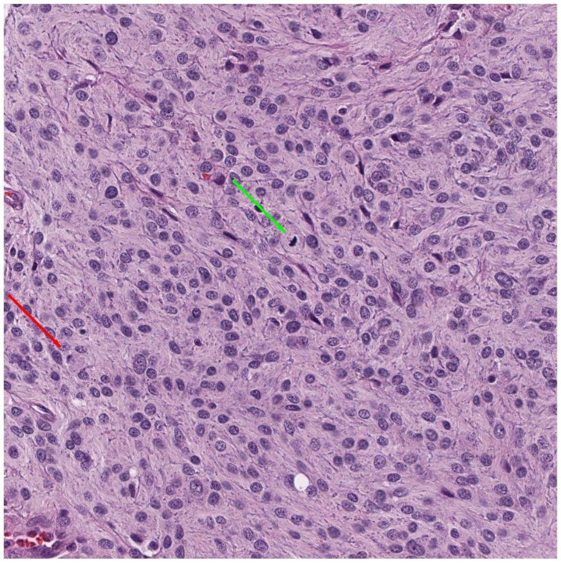

Phosphorylated Histone H3 (0.16mm^2)

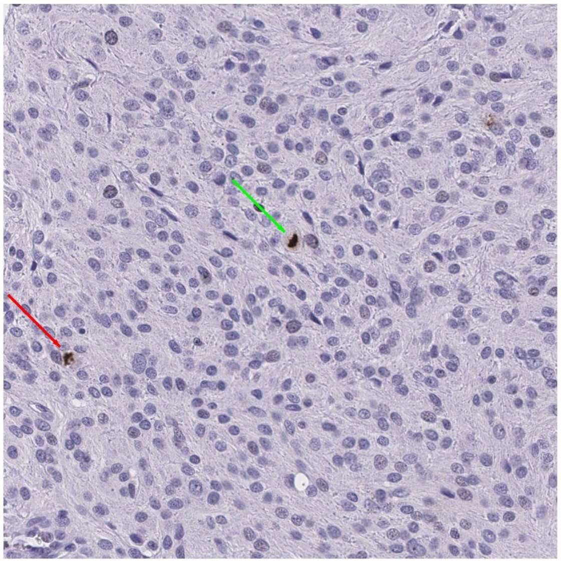

Green arrow: correct (True Positive)
Red arrow: missed mitosis (False Negative)
Blue arrow: wrong label (False Positive)

H&E (1HPF, 0.16mm^2)

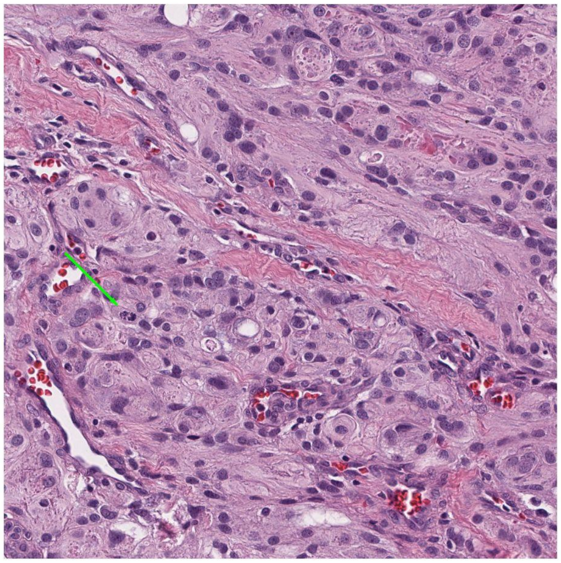

Phosphorylated Histone H3 (0.16mm^2)

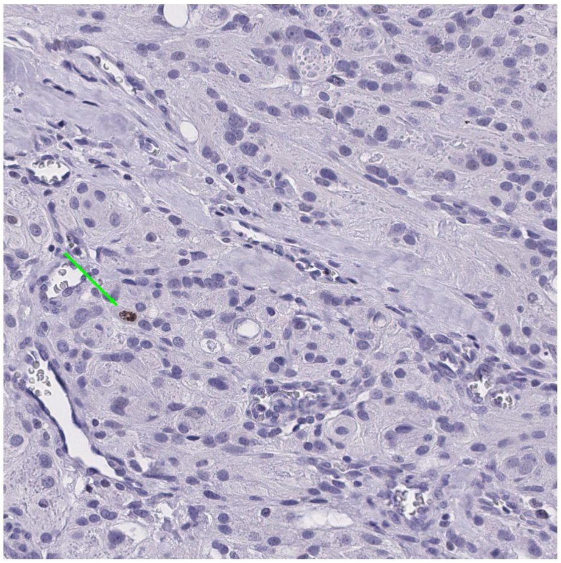

Green arrow: correct (True Positive)
Red arrow: missed mitosis (False Negative)
Blue arrow: wrong label (False Positive)

H&E (1HPF, 0.16mm^2)

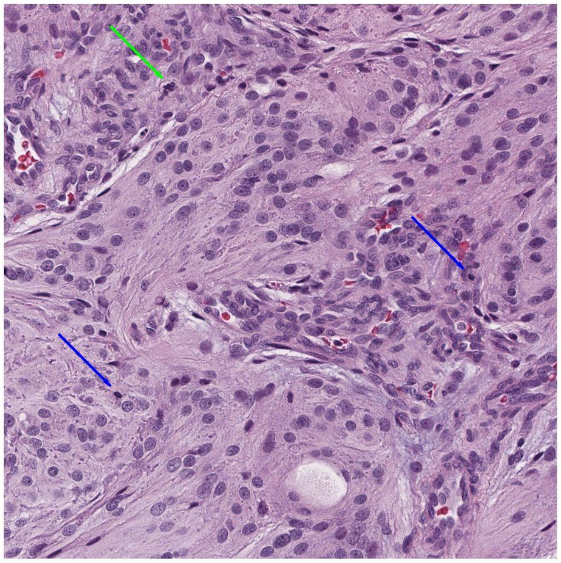

Phosphorylated Histone H3 (0.16mm^2)

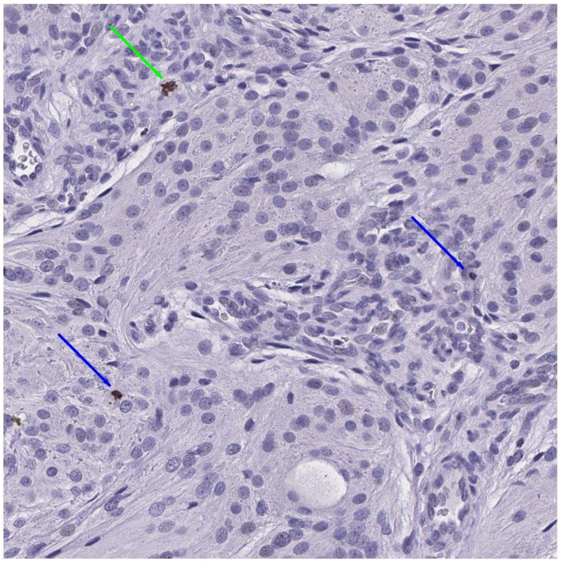

Green arrow: correct (True Positive)
Red arrow: missed mitosis (False Negative)
Blue arrow: wrong label (False Positive)

H&E (1HPF, 0.16mm^2)

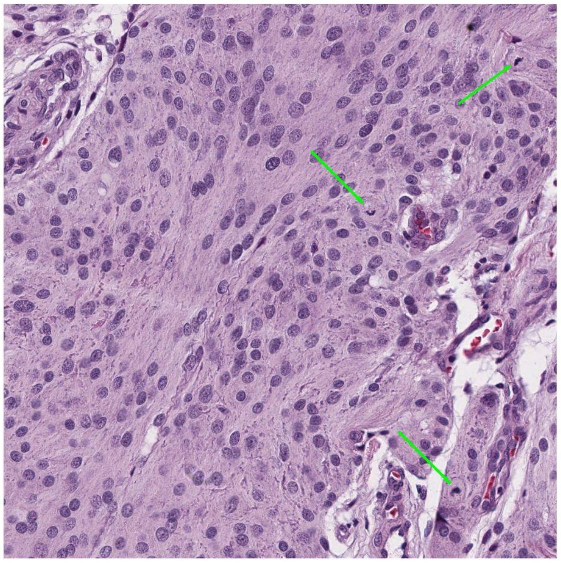

Phosphorylated Histone H3 (0.16mm^2)

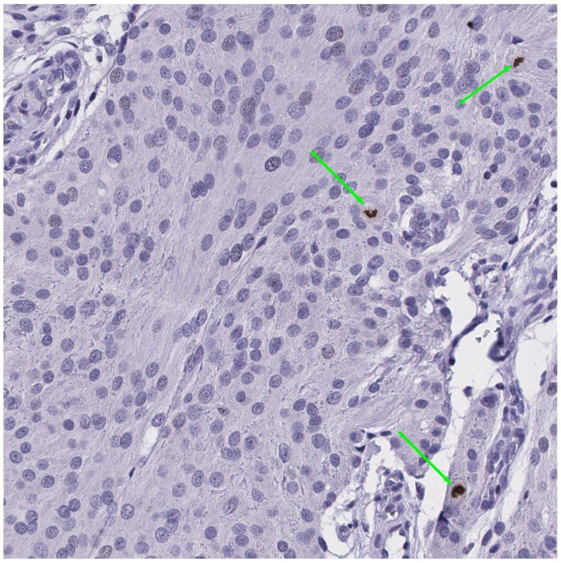

Green arrow: correct (True Positive)
Red arrow: missed mitosis (False Negative)
Blue arrow: wrong label (False Positive)

H&E (1HPF, 0.16mm^2) Phosphorylated Histone H3 (0.16mm^2)

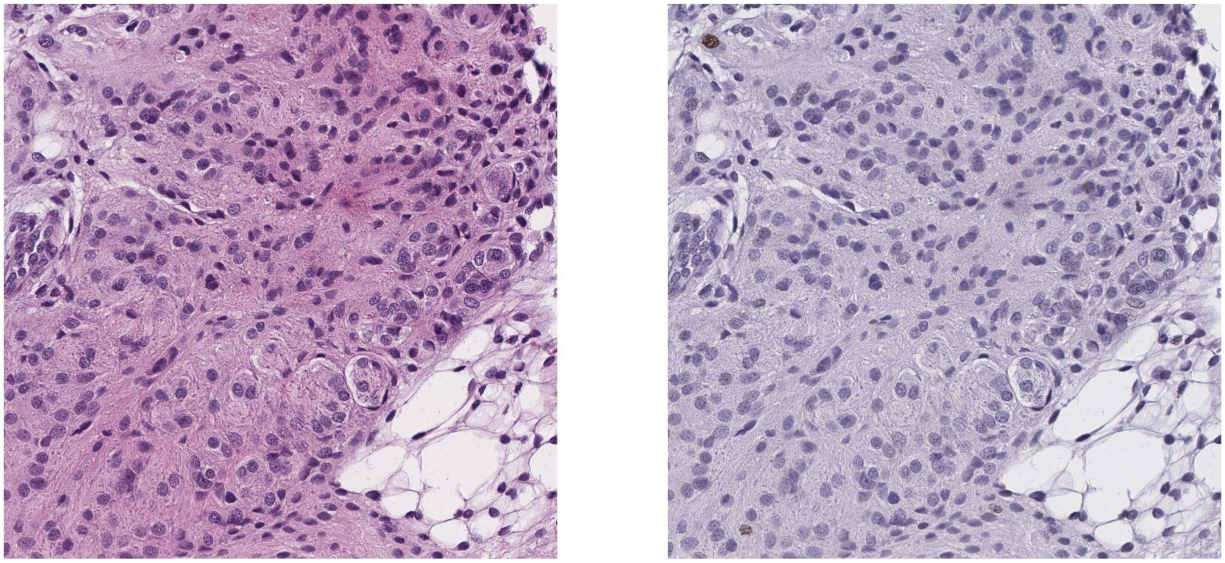

Green arrow: correct (True Positive)
Red arrow: missed mitosis (False Negative)
Blue arrow: wrong label (False Positive)

H&E (1HPF, 0.16mm^2) Phosphorylated Histone H3 (0.16mm^2)

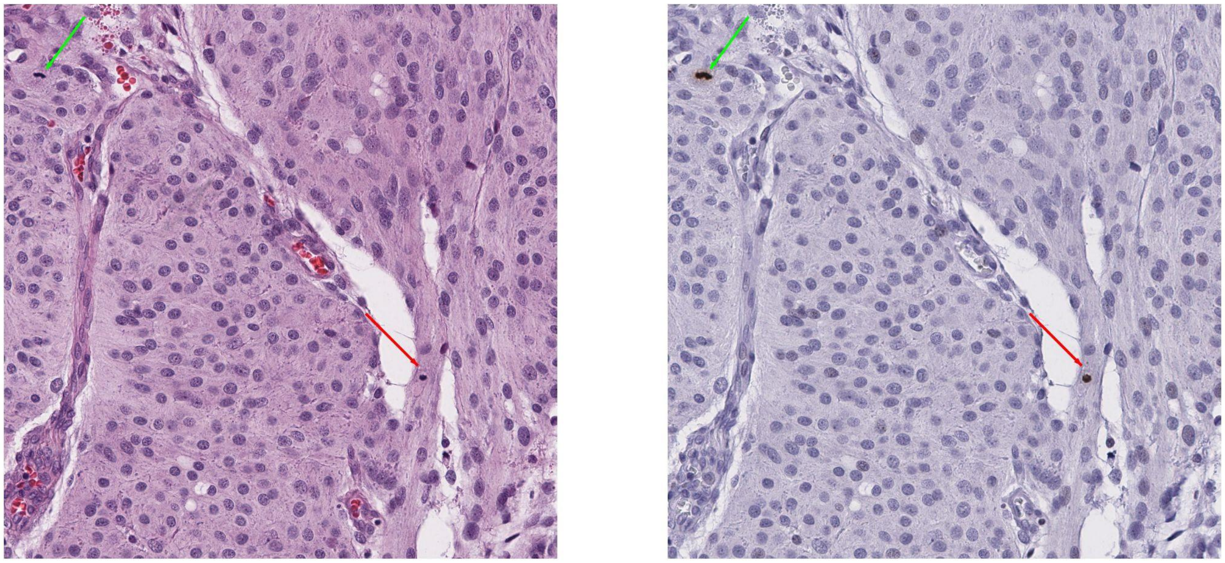

Green arrow: correct (True Positive)
Red arrow: missed mitosis (False Negative)
Blue arrow: wrong label (False Positive)

H&E (1HPF, 0.16mm^2)

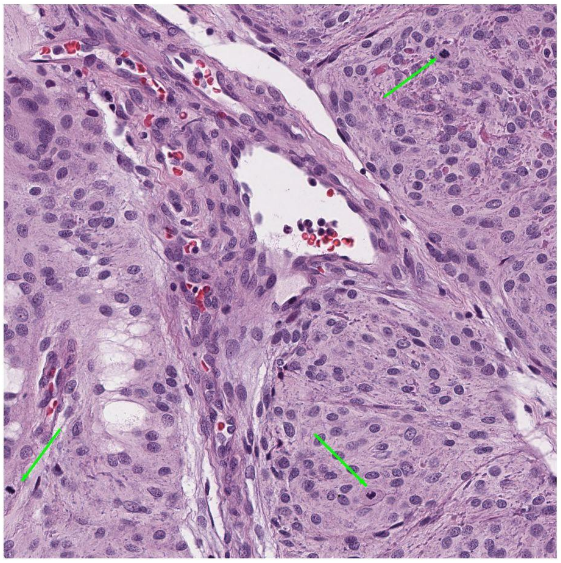

Phosphorylated Histone H3 (0.16mm^2)

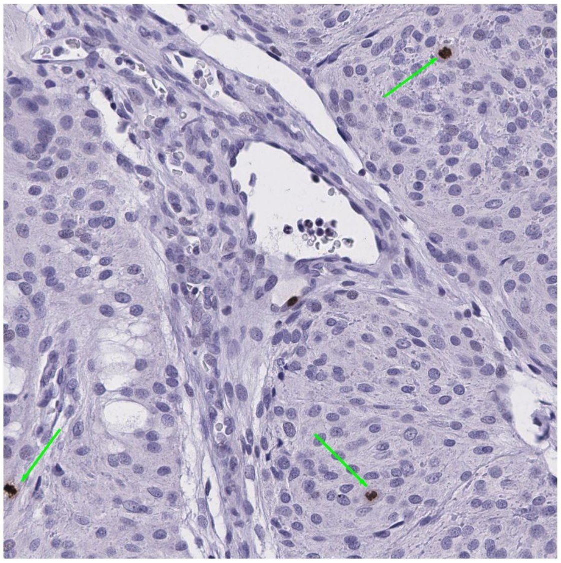

Green arrow: correct (True Positive)
Red arrow: missed mitosis (False Negative)
Blue arrow: wrong label (False Positive)

H&E (1HPF, 0.16mm^2)

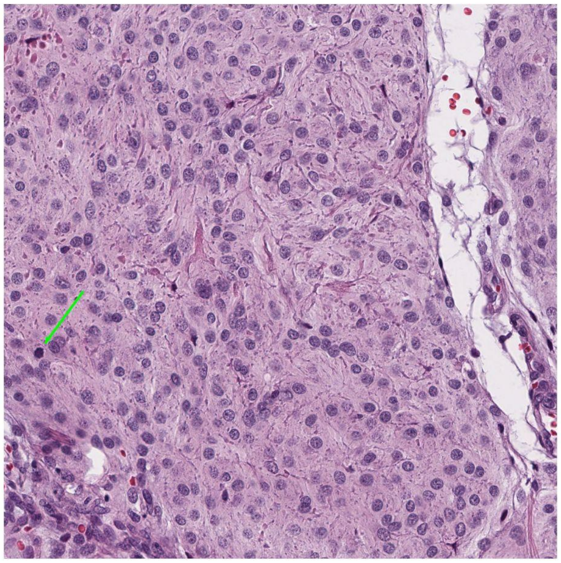

Phosphorylated Histone H3 (0.16mm^2)

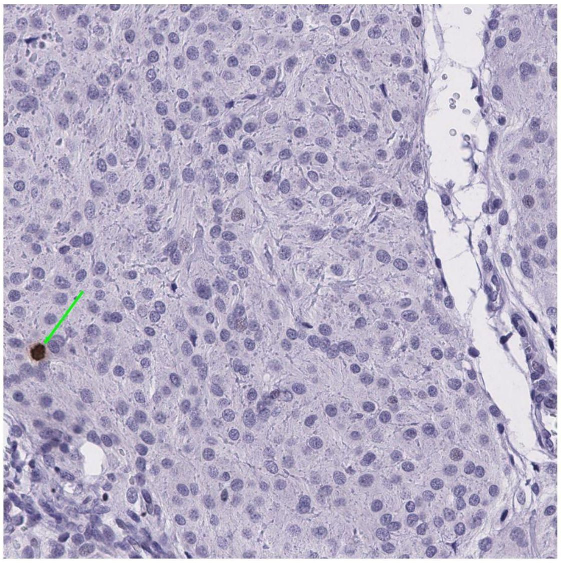

Green arrow: correct (True Positive)
Red arrow: missed mitosis (False Negative)
Blue arrow: wrong label (False Positive)

H&E (1HPF, 0.16mm^2)

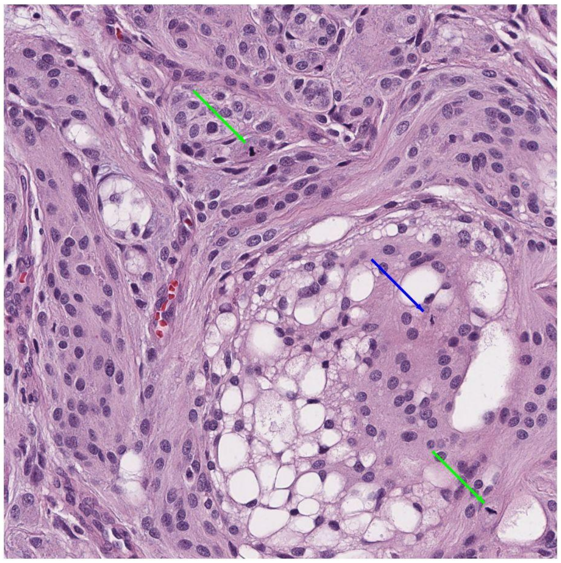

Phosphorylated Histone H3 (0.16mm^2)

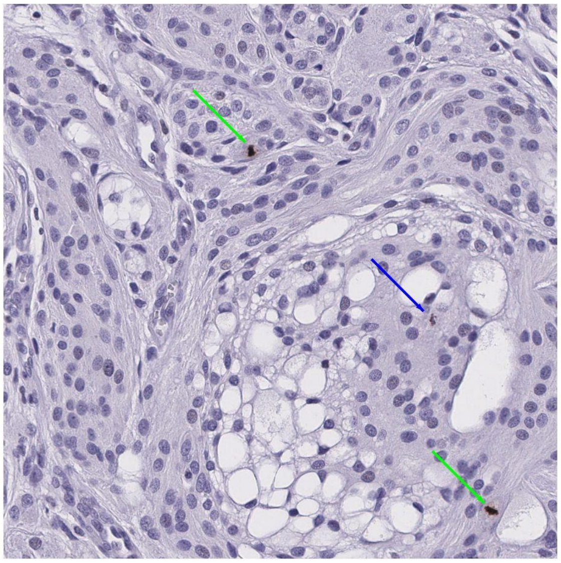

Green arrow: correct (True Positive)
Red arrow: missed mitosis (False Negative)
Blue arrow: wrong label (False Positive)

H&E (1HPF, 0.16mm^2)

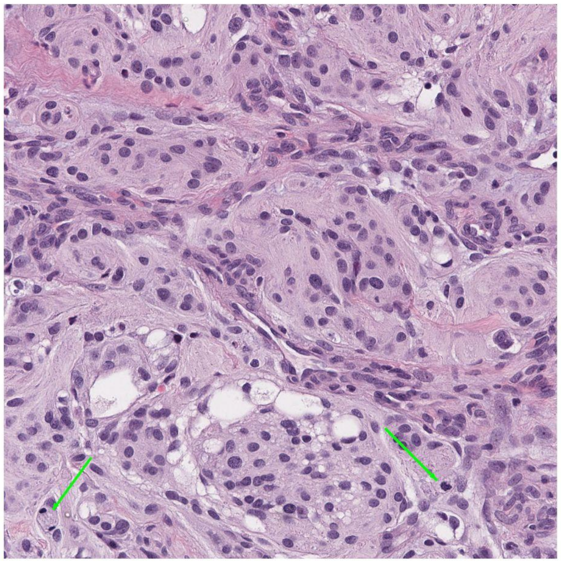

Phosphorylated Histone H3 (0.16mm^2)

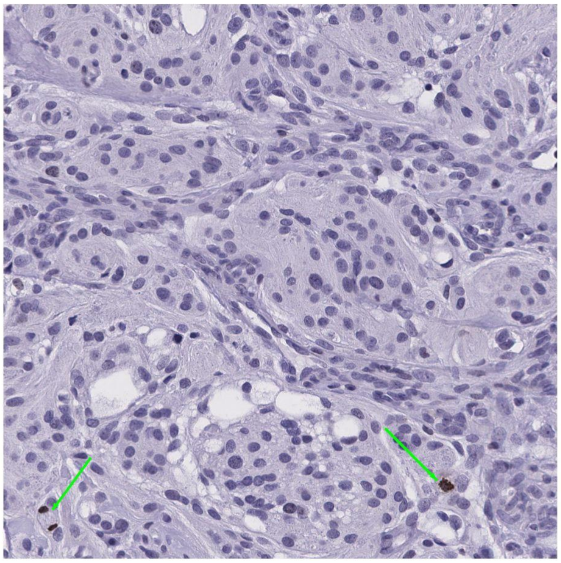

Green arrow: correct (True Positive)
Red arrow: missed mitosis (False Negative)
Blue arrow: wrong label (False Positive)

H&E (1HPF, 0.16mm^2)

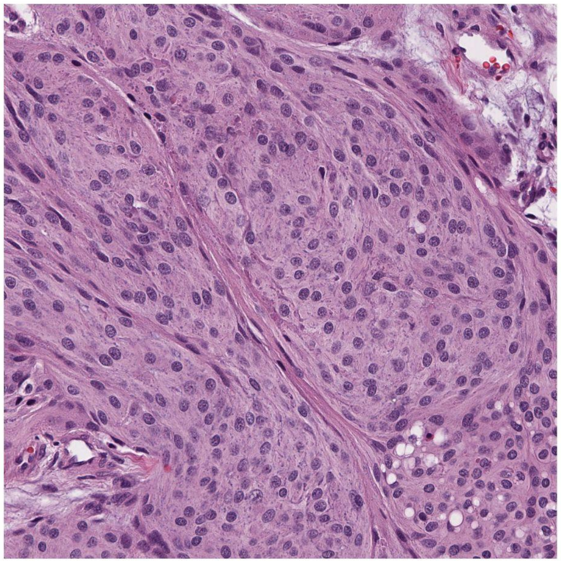

Phosphorylated Histone H3 (0.16mm^2)

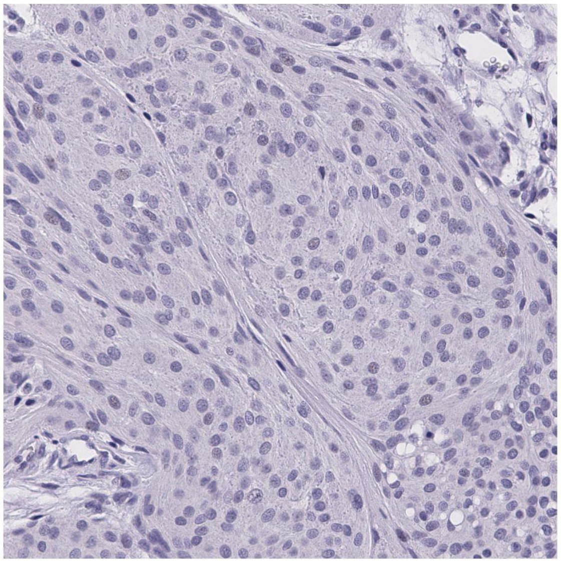

Green arrow: correct (True Positive)
Red arrow: missed mitosis (False Negative)
Blue arrow: wrong label (False Positive)

H&E (1HPF, 0.16mm^2)

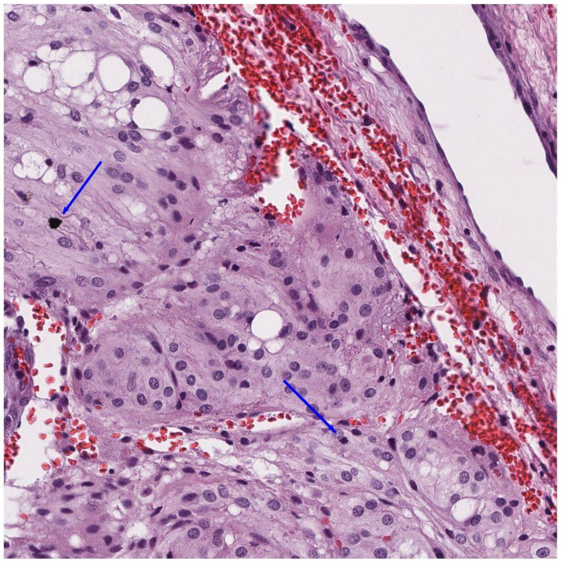

Phosphorylated Histone H3 (0.16mm^2)

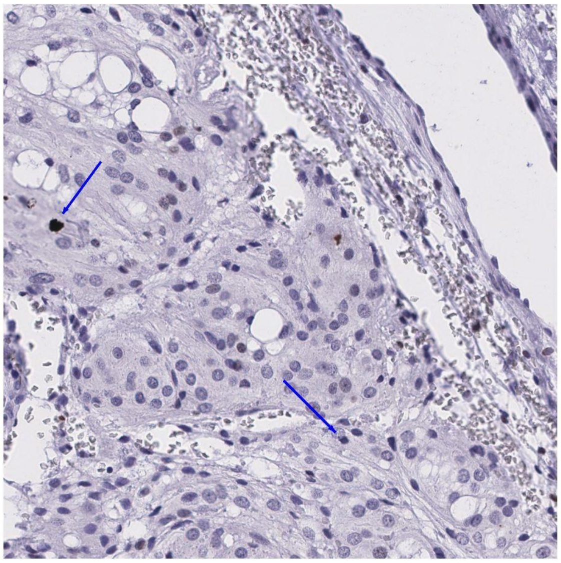

Green arrow: correct (True Positive)
Red arrow: missed mitosis (False Negative)
Blue arrow: wrong label (False Positive)

H&E (1HPF, $0.16mm^2$)

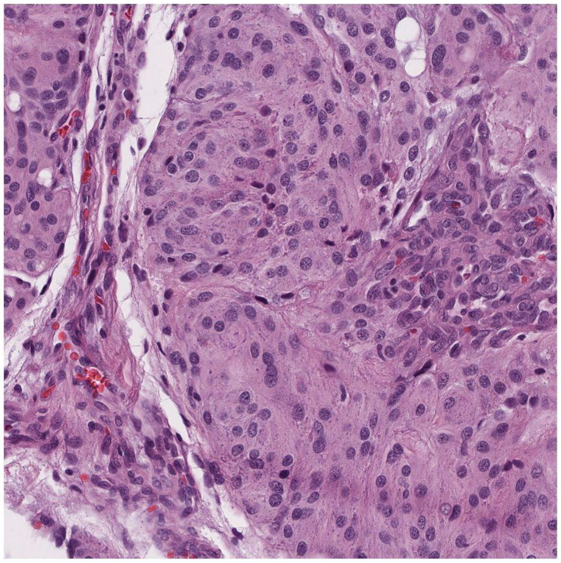

Phosphorylated Histone H3 ($0.16mm^2$)

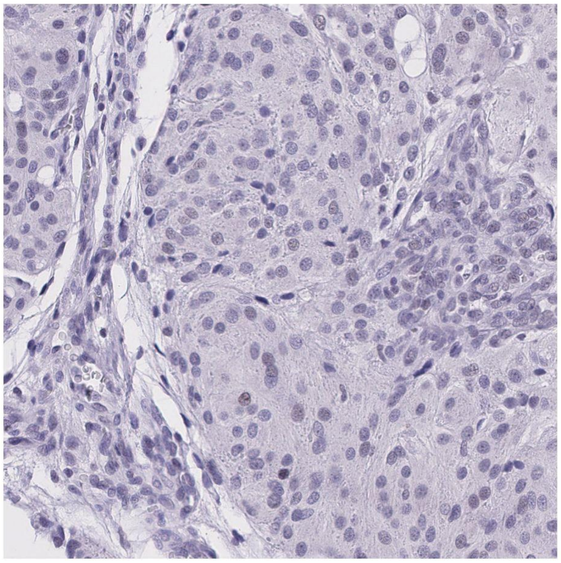

Green arrow: correct (True Positive)
Red arrow: missed mitosis (False Negative)
Blue arrow: wrong label (False Positive)

H&E (1HPF, $0.16mm^2$)

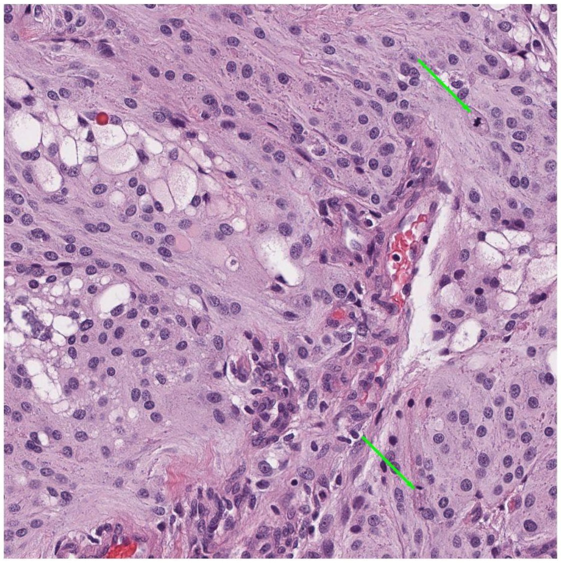

Phosphorylated Histone H3 ($0.16mm^2$)

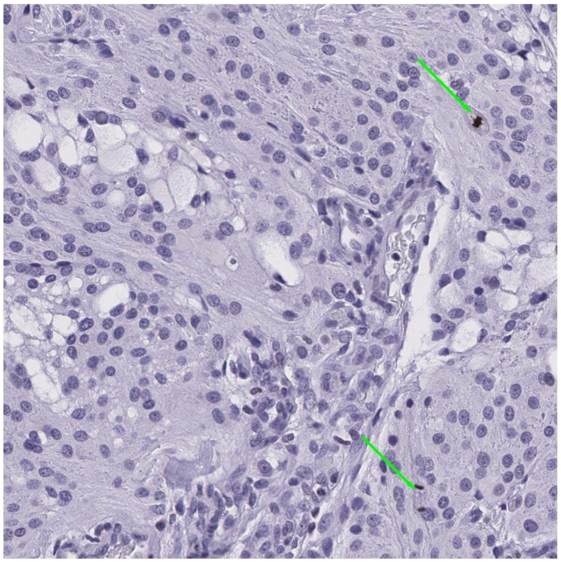

Green arrow: correct (True Positive)
Red arrow: missed mitosis (False Negative)
Blue arrow: wrong label (False Positive)

H&E (1HPF, 0.16mm^2)

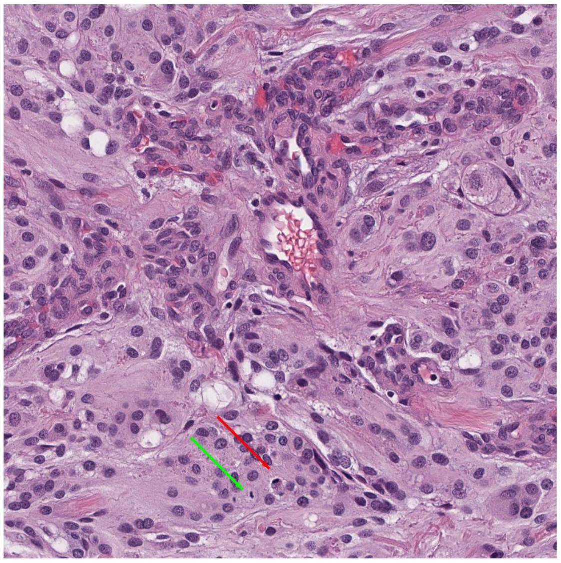

Phosphorylated Histone H3 (0.16mm^2)

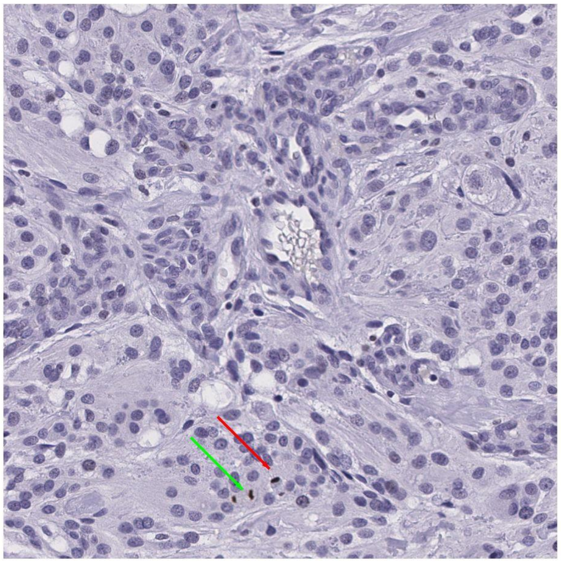

Green arrow: correct (True Positive)
Red arrow: missed mitosis (False Negative)
Blue arrow: wrong label (False Positive)

H&E (1HPF, 0.16mm^2)

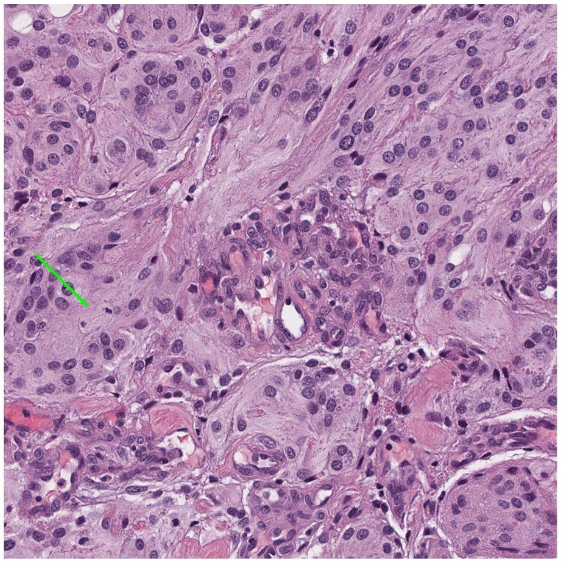

Phosphorylated Histone H3 (0.16mm^2)

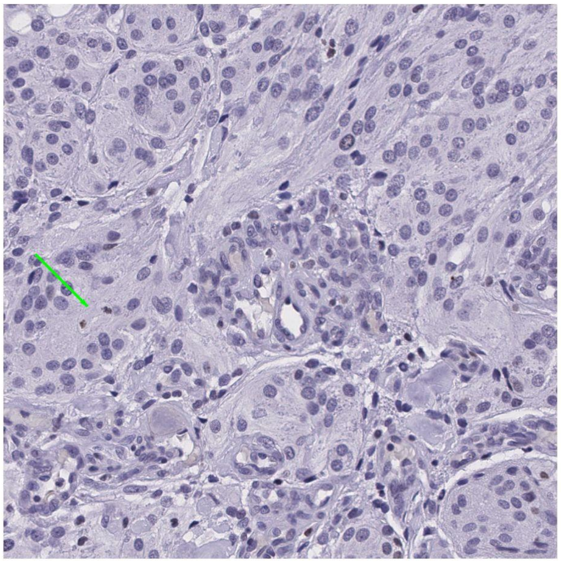

Supplement: Supplementary file 7 — Additional file 7: Performance report of the top-2 participants that achieved the best performance in the user study (2/2). [file 40478_2023_1707_MOESM7_ESM.pdf]
